# Hemoglobin decrease predicts untoward outcomes better than severity of anemia

**DOI:** 10.1038/s41598-024-82237-6

**Published:** 2024-12-28

**Authors:** Brigitta Teutsch, Zsolt Abonyi Tóth, Orsolya Ferencz, Nóra Vörhendi, Orsolya Anna Simon, Eszter Boros, Dániel Pálinkás, Levente Frim, Edina Tari, Patrícia Kalló, Endre Botond Gagyi, Tamás Hussein, Szilárd Váncsa, Vivien Vass, Andrea Szentesi, Áron Vincze, Ferenc Izbéki, Péter Hegyi, Roland Hágendorn, Imre Szabó, Bálint Erőss

**Affiliations:** 1 Institute for Translational Medicine, Medical School, University of Pécs, Pécs, Hungary; 2 Centre for Translational Medicine, Semmelweis University, Budapest, Hungary; 3 Department of Radiology, Medical Imaging Centre, Semmelweis University, Budapest, Hungary; 4 Department of Biostatistics, University of Veterinary Medicine, Budapest, Hungary; 5 First Department of Medicine, Medical School, University of Pécs, Pécs, Hungary; 6 First Department of Internal Medicine, Fejér County Szent György University Teaching Hospital, Székesfehérvár, Hungary; 7 Department of Gastroenterology, Central Hospital of Nothern Pest – Military Hospital, Budapest, Hungary; 8 Selye János Doctoral College for Advanced Studies, Semmelweis University, Budapest, Hungary; 9 Institute of Pancreatic Diseases, Semmelweis University, Budapest, Hungary

**Keywords:** Hemoglobin decrease, Delta hemoglobin, Restrictive, Transfusion, Gastroenterology, Gastrointestinal diseases, Gastrointestinal system

## Abstract

Patients with gastrointestinal bleeding (GIB) exhibit varying tolerances to acute blood loss. We aimed to investigate the effect of relative Hb decrease (ΔHb%) on GIB outcomes. Participants enrolled in the Hungarian GIB Registry between 2019 and 2022 were analyzed. The primary outcome, defined as a composite endpoint, included in-hospital bleeding-related mortality and the need for urgent intervention. Four groups were created based on the lowest Hb measured during hospitalization (nadirHb), along with four subgroups categorized by ΔHb%. Regardless of the nadirHb, participants with higher ΔHb% had a higher probability of reaching the composite endpoint. A 30–40% ΔHb% decrease to a nadirHb of 80–90 g/L resulted in a similar likelihood of reaching the primary endpoint as a 0–10% ΔHb% to 70–80 g/L or 60–70 g/L, respectively (10% vs. 12%, *p* = 1.00; 10% vs. 10%, *p* = 1.00). Our results showed that a higher Hb decrease in GIB is associated with an increased untoward outcome rate even when the lowest hemoglobin exceeds the recommended transfusion thresholds. New randomized controlled trials investigating transfusion thresholds should consider ΔHb% as a potential key variable and risk factor.

**Supplementary Information:**

The online version contains supplementary material available at 10.1038/s41598-024-82237-6.

## Introduction

### Background and rationale

Gastrointestinal bleeding (GIB) is a common medical emergency. Despite its decreasing incidence and significant advances in its management, the associated mortality rate remains high^1,2^. Treatment of acute blood loss can be lifesaving in moderate and severely anemic patients. Therefore, administration of red blood cells (RBC) is essential to improve tissue microcirculation and oxygenization^3^.

Multiple publications have already confirmed the efficacy and safety of restrictive RBC transfusion strategy (initiated at a hemoglobin level (Hb) of 70–80 g/L) compared to a more liberal approach (90–100 g/L) in upper GIB. However, randomized controlled trials (RCTs) have emplied varying thresholds to define the start of the intervention^4–7^. A recent meta-analysis based on RCTs, mostly excluding participants with major cardiovascular comorbidities, showed that initiating transfusion at a Hb level > 70 g/L compared to a threshold > 80 g/L resulted in a slightly reduced 30-day rebleeding rate (5% vs. 7%), and incidence of acute kidney injury (8% vs. 9%)^8^.

Current GIB guidelines recommend a transfusion threshold of 70 g/L for most patients and 80 g/L for those with cardiovascular comorbidities^9,10^. However, individual tolerance to anemia varies considerably. Lack of adaptation to blood loss from a normal baseline Hb reaching transfusion trigger can lead to a higher rate of unwanted outcomes^11–13^. Extensive cohort studies in surgical populations have shown that the relative decrease of Hb (ΔHb%) from the baseline warrants more attention than the lowest hemoglobin (nadirHb). Their results highlighted that when ΔHb% exceeds 50%, surgical patients faced a higher mortality risk, even if the nadirHb, as a transfusion trigger, remained above 70 g/L^14,15.^

Baseline Hb and its change as a guide for transfusion, as well as its predictive value for in-hospital bleeding-related mortality and need for urgent intervention have not yet been investigated in GIB. However, it would be essential to consider the individual variabilities of nadirHb and ΔHb%, as these factors significantly influence bleeding tolerance.

### Objectives

Our primary objectives were to (1) assess the association between RBC transfusion and different ranges of nadirHb, (2) determine the absolute and relative cut-off point of significant Hb change, and (3) investigate how the nadirHb and the related ΔHb% can influence the outcomes of GIB.

## Methods

Our cohort analysis is reported based on the recommendations of the STROBE guideline (Table S1)^16^.

### **Data sources** and study setting

The study includes data from the Hungarian Gastrointestinal Bleeding Registry (Hungarian Medical Research Council ethical approval number: 24433-5/2019/EÜIG)^17.^ Data were collected pro- and retrospectively in two Hungarian tertiary hospitals (University of Pécs Medical School, Pécs; and Fejér County Szent György University Teaching Hospital, Székesfehérvár) between November 1, 2019 and August 31, 2022. All participants provided written informed consent, the data collection was performed in accordance with the Declaration of Helsinki.

### Participants

All adult patients (> 18 years) who presented with manifest GIB regardless of the source (melena, hematochezia, hematemesis, blood in the nasogastric tube, or active bleeding identified during endoscopy) were eligible for enrollment in the Registry. Data were collected daily during hospitalization and recorded in electronic case report forms^17^.

### Outcomes

Following recent recommendation for relevant outcome assessment in GIB-related clinical studies, we designated a composite endpoint as the primary outcome, consisting of in-hospital bleeding-related mortality and the need for urgent intervention (including repeat endoscopy due to clinical signs of rebleeding, need for surgery or trans-arterial embolization)^18^. In addition, in-hospital bleeding-related mortality and the need for urgent intervention were assessed separately as secondary outcomes.

### Variables

#### NadirHb

As a first step, we selected participants with documented nadirHb recorded during their hospital stay. As this variable was included in additional models to predict the outcomes of interest, only measurements taken prior to the specific endpoint were included in the analyses. Therefore, when both urgent intervention and bleeding-related mortality occured, two different Hb levels were used to predict outcomes. For the composite endpoint, we included the Hb measurement taken before the need for urgent intervention, as this represented the first outcome reached by the participant.

#### Association between transfusion and nadirHb (analysis 1)

In medical practice, RBC transfusion is recommended to be initiated at a lower Hb level, preferably < 70 g/L. Given that the recommended trigger for RBC replacement varies in the medical literature, our first analysis focused on exploring the association between nadir Hb and transfusion. Participants who received RBC transfusion were divided into four groups based on their nadirHb levels: ≤60 g/L, > 60-≤70 g/L, > 70-≤80 g/L, > 80 g/L. Details of the multivariable analysis are included in the Supplementary Material.

Following GIB, patients may receive platelets, fresh frozen plasma, or coagulation factor concentrates. These cases were included in our analyses to avoid excluding more severe cases, which would have resulted in findings that did not accurately represent the general GIB population.

#### Hemoglobin change (analysis 2)

As the change in the nadirHb level may also be associated with the outcome rate, we calculated the delta Hb (ΔHb) to evaluate how a decrease in Hb predicts the endpoint. Only cases with at least two Hb measurements - one on the day of admission and one during hospitalization – were eligible for analysis. ΔHb was determined as the difference between the first admission Hb and the nadirHb. As some participants received transfusion even after their source of bleeding was treated, this resulted in a Hb increase after admission. Those cases where the Hb showed only an increase, were excluded. To calculate the relative decrease in the ΔHb, we used the following formula: ΔHb% = [(firstHb – nadirHb)/firstHb]*100. In addition, decrease in ΔHb and ΔHb% per hour was also assessed.

#### ΔHb and nadirHb (analysis 3)

Participants might tolerate greater blood loss differently even if the nadirHb is above the recommended transfusion trigger level. Therefore, as a third analysis, we evaluated Hb decrease by considering the achieved nadirHb. We categorized participants into four groups based on nadirHb levels (> 50-≤60, > 60-≤70, > 70-≤80, > 80-≤90 g/L) and into four subgroups by the ΔHb% decrease (0-≤10, > 10-≤20, > 20-≤30, and > 30-≤40%). RBC transfused participants were analyzed separately.

### Statistical analysis

In the baseline table, continuous variables are reported with means and standard deviations (SD), and categorical variables are shown as percentages.

For Analysis 1, we calculated unadjusted odds ratios (OR) and adjusted ORs (aOR) with 95% confidence intervals (CI). A group with > 60-≤70 g/L Hb was chosen as a reference, in line with current guideline recommendations for Hb thresholds. A multivariable analysis was conducted using logistic regression, following model selection based on the Akaike information criterion (AIC), and included the specified variables and their two-way interactions, excluding any that exhibited multicollinearity. Dunnett’s post-hoc test was applied to compare the Hb value groups with the reference group. A p-value < 0.05 was considered significant.

Using the prediction model for Analysis 2, we calculated cut-off values and their associated sensitivity and specificity using the pROC R 1.18.5 package. The area under the curve (AUC) with 95% CIs was interpreted as follows: ≥0.9 as excellent, 0.8–0.9 as considerable, 0.7–0.8 as fair, 0.6–0.7 as poor, and 0.5–0.6 as failure^19^.

For Analysis 3, we employed binomial logistic regression to predict event probabilities. The explanatory variables were the nadirHb and ΔHb% as continuous variables and their interaction. Probability estimated were generated at 16 points, positioned at the midpoint within each of the four each nadir and delta groups. Tukey’s post-hoc test was used to compare subgroup, with statistical significance set at p-value < 0.05. In addition, ORs with 95% CIs, standard errors, and Z ratios were also calculated.

We excluded records with missing data. For Analysis 2, all observations missing any explanatory variables were removed. Data quality for each included variable in the analysis is shown in Table S2.

All analyses were conducted with R v. 4.3.1 with pROC 1.18.5 and emmeans 1.10.2 packages.

## Results

### Participants

A total of 1021 cases were enrolled in the Registry during data collection.

**Fig. 1:**
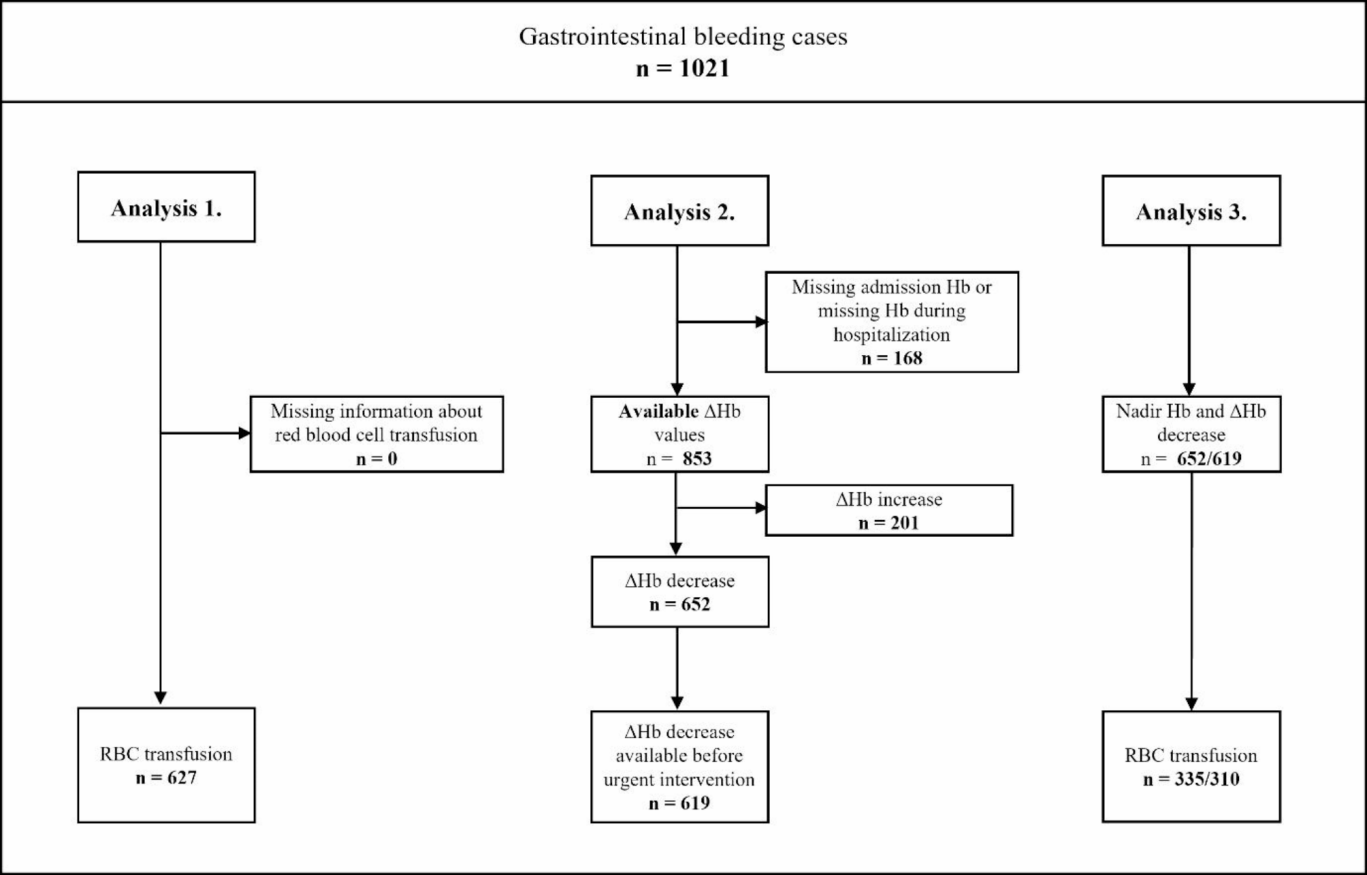
Flow diagram of selection. RBC: red blood cell, Hb: hemoglobin.

Three different analyses were conducted, and the number of cases included in each is shown in Fig. 1.

### Baseline characteristics

The baseline characteristics of the included population can be found in Table 1.

#### Lower nadirHb does not result in significantly increased odds of developing the outcome (Analysis 1)

When assessing the lowest Hb measured during hospitalization in patients receiving RBC transfusion, we observed that the outcome rate was slightly lower in the 70–80 g/L group compared to a 60–70 g/L nadirHb group. However, none of the groups showed significantly different odds of developing the composite endpoint, nor were there significant differences in bleeding-related mortality or the need for urgent intervention separately (Table 2).

#### ΔHb alone cannot predict the outcomes of GIB (Analysis 2)

**Fig. 2:**
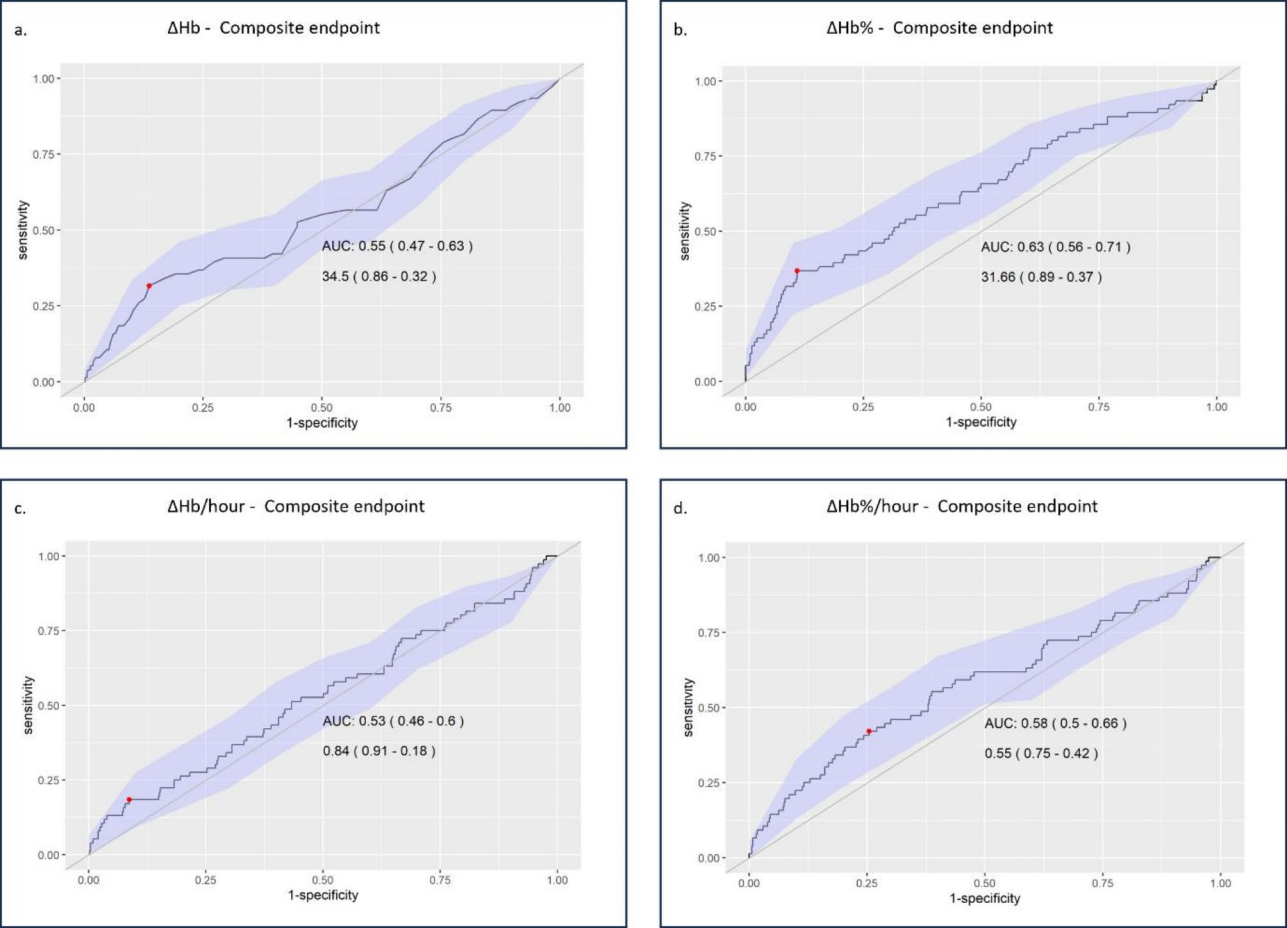
Prediction of the composite endpoint based on the ΔHb (a.), ΔHb% (b.), ΔHb/hour (c.), and ΔHb%/hour (d.). Data in each figure represent the area under the curve (AUC) with 95% confidence intervals (first row) and the cut-off hemoglobin values with related specificity and sensitivity (second row).

Next, we assessed whether the ΔHb and the ΔHb% could predict the outcome of interest across all patients included. We found that both absolute and relative Hb decreases, as well as their hourly detection rates, could distinguish patients who did not develop the composite endpoint with high specificity; however, the diagnostic performance of the test was poor or failed across all cases (Fig. 2).

Prediction models with ΔHb and ΔHb% decrease yielded similar results when assessed separately for bleeding-related mortality (Fig. S1) and need for urgent intervention (Fig. S2).

#### Higher ΔHb% leads to increased outcome probability (Analysis 3)

##### Composite endpoint

**Fig. 3:**
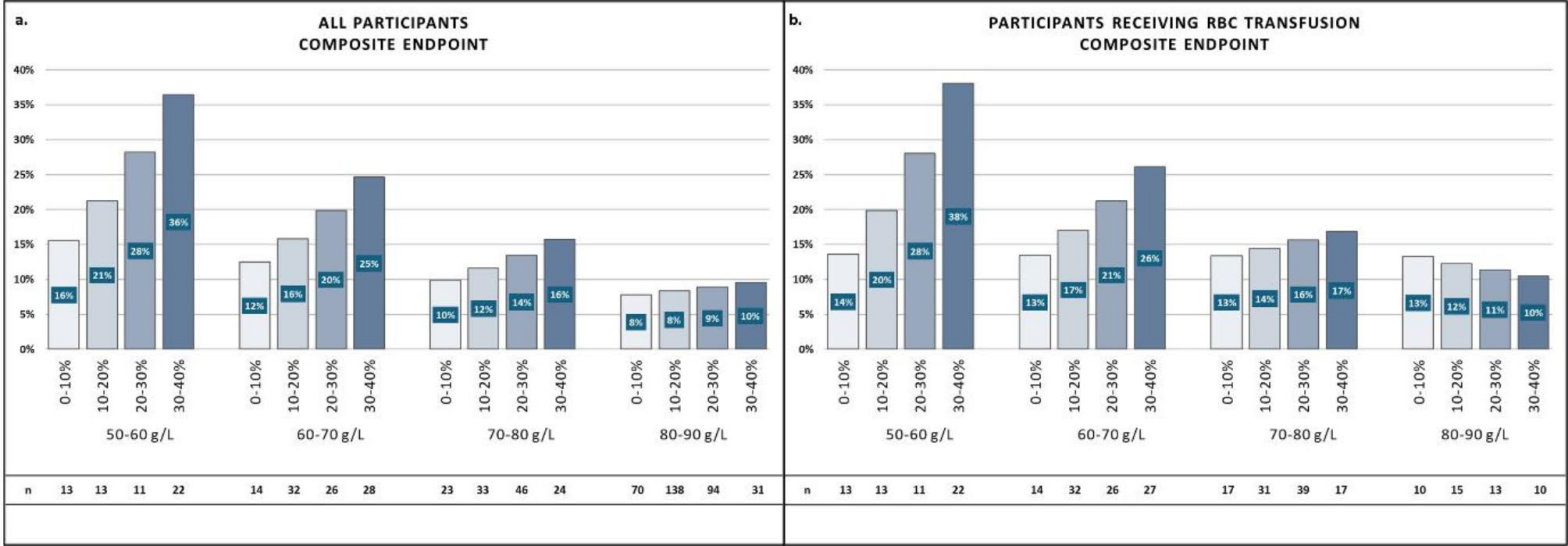
Rate of composite endpoint (bar height in %) in the four groups of nadirHb (50–60, 60–70, 70–80, and 80–90 g/L) and the four subgroups of ΔHb% (0–10, 10–20, 20–30, and 30–40%) for all participants (a) and participants receiving red blood cell (RBC) transfusion (b). The sample size of the subgroups is shown below each bar.

When dividing participants by the ΔHb% and nadirHb, we observed that as the ΔHb% increased, the probability of reaching the composite endpoint rose across all participants. Although the probability of outcome rate doubled, the increase was not statistically significantly when comparing the 0–10% ΔHb% subgroups in the nadirHb groups of 80–90 g/L and 50–60 g/L (8% vs. 16%; OR: 2.18, CI: 0.70–6.75; *p* = 0.581). However, the 30–40% subgroup with the same nadir levels had almost 5.5-times higher odds of reaching the composite endpoint (10% vs. 36%; OR: 5.40, CI: 1.52–19.15; *p* < 0.001). In addition, a 30–40% Hb decrease of in the 80–90 g/L group resulted in a similar probability of outcome rate as a 0–10% decrease in the 60–70 g/L or 70–80 g/L groups, respectively (10% vs. 12%; *p* = 1.00; 10% vs. 10%; *p* = 1.00) (Fig. 3a, Table S3).

Moreover, in the RBC-transfused population withing the subgroup experiencing a 0–10% decrease, the probability of reaching the composite outcome was consistent regardless of how low the nadirHb was (13% vs. 13% vs. 13% vs. 14%). A 30–40% Hb decrease resulted in larger, though statistically not significant, differences between the nadir groups compared to a nadirHb of 80–90 g/L (10% vs. 17%; OR: 1.74, CI: 0.97–3.13; *p* = 0.090; 10% vs. 26%; OR: 3.03, CI: 0.94–9.77; *p* = 0.090; 10% vs. 38%; OR: 5.26, CI: 0.91–30.56; *p* = 0.090). Although with a nadirHb between 70 and 80 g/L in all ΔHb% subgroups, the probability of reaching the composite endpoint was between 13 and 17%, in the 60–70 g/L group when comparing the 0–10% and 30–40% decrease subgroups, the probability doubled, though this difference was not statistically significant (13% vs. 26%; OR: 0.44, CI: 0.12–1.59; *p* = 0.710) (Fig. 3b, Table S4).

##### Bleeding-related mortality

**Fig. 4:**
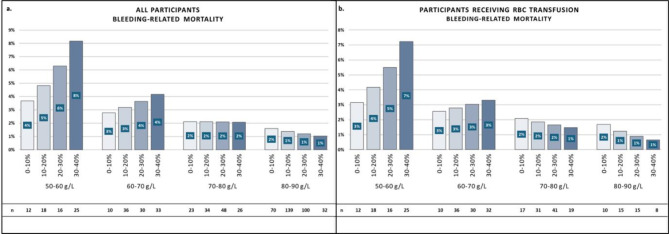
Rate of bleeding-related mortality (bar height in %) in the four groups of nadirHb (50–60, 60–70, 70–80, and 80–90 g/L) and the four subgroups of ΔHb% (0–10, 10–20, 20–30, and 30–40%) for all participants (a) and participants receiving red blood cell (RBC) transfusion (b). The sample size of the subgroups is shown below each bar.

The probability of bleeding-related mortality ranged from 1 to 4% in all subgroups of nadirHb between 60 and 90 g/L. A ΔHb% decrease of 30–40% to a nadirHb of 60–70 g/L predicted a similar outcome rate as a 0–10% decrease to a Hb level of 50–60 g/L (4% vs. 4%; *p* = 1.00). In addition, in the latter group, a 30–40% decrease was associated with a twofold higher odds of the outcome compared to the 0–10% decrease group (8% vs. 4%; OR: 2.05, CI: 1.036–4.05; *p* = 0.028) (Fig. 4a, Table S5). The transfused population exhibited similar outcome rates (Fig. 4b, Table S6).

##### Need for urgent intervention

**Fig. 5:**
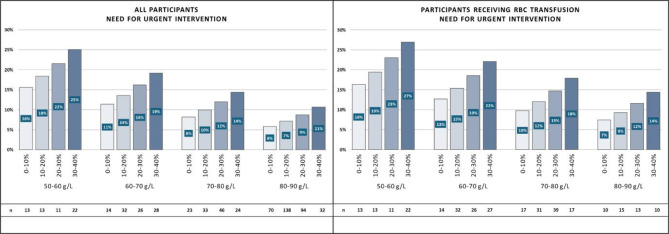
Rates of need for urgent intervention (bar height in %) in the four groups of nadirHb (50–60, 60–70, 70–80, and 80–90 g/L) and the four subgroups of ΔHb% (0–10, 10–20, 20–30, and 30–40%) for all participants (a) and participants receiving red blood cell (RBC) transfusion (b). The sample size of the subgroups is shown below each bar.

Participants with a Hb decrease of 30–40% reaching a nadirHb of 80–90 g/L were predicted to achieve a similar rate of outcome as those with a 0–10% in the nadirHb group of 60–70 g/L (11% vs. 11%; *p* = 1.00) (Fig. 5.a, Table S7). Although the transfused population had a 30–40% Hb decrease to a nadir of 80–90 g/L, 14% of the patients had the probability of receiving urgent intervention. Similar findings were observed for a 0–10% decrease to a nadirHb of 50–60 g/L and 60–70 g/L, respectively (14% vs. 16%; *p* = 1.00; 14% vs. 13%; *p* = 1.00) (Fig. 5.b, Table S8).

## Discussion

Multiple surgical studies have assessed changes in Hb levels and ther association with hard clinical endpoints^14,15,20–22^. However, to our knowledge, this is the first study to investigate this relationship in the context of acute GIB. In our analysis, we initially assessed the odds of developing unwanted outcomes in patients who received RBC transfusion. We found no significant differences between the nadirHb groups when compared to a Hb level between 60 and 70 g/L, though it is noteworthy that the lowest outcome rate occurred in the group where the nadirHb was between 70 and 80 g/L. Secondly, our findings demonstarted that ΔHb% exhibited high specificity; however, neither the ΔHb nor the ΔHb% could predict the outcome of interest with high accuracy. Our third analysis, however, confirmed our hypothesis that a higher amount of blood loss could result in an elevated probability and odds of bleeding-related in-hospital mortality or need for urgent intervention during hospital stay even when the nadirHb exceeds 70 g/L.

Individual tolerance to blood loss can vary significantly. Transfusion at a lower Hb threshold does not appear to produce notable differences in in-hospital and 30–42 day mortality, nor in rated of rebleeding^8^. Our first analysis supports these findings. Administering RBCs at a lower threshold is generally considered safe and also cost-effective. Especially now, in times of a global shortage of blood products, it is crucial to provide this treatment only when needed^23^. Moreover, transfusion also carries a risk of multiple unfavorable outcomes, including transfusion-associated circulatory overload, transfusion-related acute lung injury, infections and coagulopathy, which can increase with the dose of RBC units^24,25^. However, it remains uncertain whether the rate of the outcomes, and the resulting in-hospital mortality or rebleeding, could be further minimized if changes in Hb levels were also taken into account.

In studies involving surgical patients, a 50% Hb decrease has been identified as threshold associated with higher rates of adverse outcomes^14,15,20,21^. Nonetheless, it is essential to note that anemic participants were excluded from these analyses. Our prediction models were unable to determine accurate Hb decrease cut-offs. In the context of GIB, patients often present with moderate to severe anemia upon admission due to acute blood loss. Only a small subset of patients, primarily those who developed iatrogenic bleeding or were already receiving inpatient care, might have laboratory measurements and full blood count before the onset of the bleeding. Consequently, in our prediction model, admission Hb was not likely to represent baseline levels of patients in most cases, which resulted in underestimating Hb change used for prediction. Given that the bleeding source can affect bleeding severity, future models should consider evaluating these patients separately, as this may enhance predictive accuracy.

Our results are consistent with those of surgical studies: greater blood loss increases the incidence of an undesired endpoint. Spolverato et al. employed a prediction model to evaluate perioperative complications, categorizing participants into groups and subgroups based on the ΔHb% and nadirHb. Among patients undergoing liver and pancreatic surgery, those with a 10% decrease to a nadir Hb of 60 g/L had a similar probability (18%) of reaching the endpoint as those with a 30% decrease to a nadir Hb of 100 g/L.^21^ Another study by the same author showed that patients receiving transfusion during major gastrointestinal surgery were nearly two and a half times more likely to experience perioperative mortality if they had a ΔHb% ≥50% and a nadir Hb ≥ 70 g/L, compared to those with a < 50% decrease at the same nadir Hb level^14^.

Changes in Hb levels might be significant not only for guiding transfusion but also for predicting GIB outcomes. Among currently used risk assessment scores—Rockall, ABC, AIMS65, Oakland, and Glasgow-Blatchford—only the Glasgow-Blatchford score incorporates Hb levels, using a threshold of 100 g/L to predict disease progression. Our findings suggest that even when the nadirHb is below 90 g/L, substantial differences exist between groups when ΔHb% is taken into account. We propose that patients with a higher ΔHb% should be considered at high risk for adverse outcomes. Among these scoring systems, ABC score currently offers the greatest accuracy for predicting adverse endpoints in both upper and lower GIB, with an AUC between 0.8 and 0.9. In contrast, the diagnostic accuracy of AIMS65, Oakland, and Glasgow-Blatchford scores has been shown to be fair to poor in large cohort analyses^26^. A systematic review of 30 studies found that artificial neural networks outperformed other machine learning models and clinical risk scores, including Glasgow-Blatchford, Rockall, Child-Pugh, and MELD, in predicting GIB outcomes^27^. It is essential that future prediction models also consider Hb decreases as a key factor.

Our analysis included a large cohort of participants with all bleeding sources. However, follow-up data were not collected in the Registry, so we could not investigate the recommended 30-day follow-up for non-variceal and 42-day follow-up for variceal upper GIB^18^. Due to the limited number of participants reaching the endpoints, it was not possible to form subgroups when investigating ΔHb cut-off values. However, future analyses should separately examine cut-offs for major comorbidities (e.g., ischemic heart disease) and different sources of bleeding (e.g., variceal bleeding). During the COVID-19 pandemic, prospective data collection was not feasible, which resulted in the unavailability of transfusion initiation times for all cases. Nevertheless, by using the nadir Hb measured prior to the outcome of interest, we avoided including Hb levels that were further lowered by secondary blood loss (e.g., rebleeding or surgery).

The results of our registry analysis highlight the importance of more individualized risk assessment. Patients with a greater blood loss may benefit from earlier interventions; therefore, clinicians should monitor more closely those with a higher ΔHb%, even if their nadirHb is above 70 g/L. Future risk scoring systems in GIB, including ΔHb% as an additional parameter for risk stratification, may lead to a more efficient resource allocation. Consideration should be given to relying only on the Hb level if the ΔHb% is available. However, future studies should thoroughly investigate the causal relationship between clinical outcomes ΔHb% and nadirHb in randomized conditions, which may lead to more precise and generalizable transfusion protocols.

In conclusion, our study highlights the importance of ΔHb% as a significant predictor of adverse outcomes. Our findings suggest that ΔHb% may serve as a more informative measure than nadir Hb alone, guiding the timing and necessity of RBC transfusions. Future research should focus on validating these findings first in larger cohorts, followed by randomized studies, to explore the potential of ΔHb% in refining transfusion protocols for GIB management.

**Table 1:** Baseline characteristics of the population investigated.

	Population transfused (Analysis 1)	ΔHb decrease (Analysis 2, 3)	Total
Number of participants	627	619	1021
Mean age (SD)	69.92 (13.22)	68.50 (13.98)	69.46 (13.56)
Male n (%)	368 (58.69)	385 (62.20)	611 (59.84)
Source of bleeding			
Non-variceal upper gastrointestinal bleeding n (%)	372 (59.33)	313 (50.57)	527 (51.62)
Variceal upper gastrointestinal bleeding n (%)	67 (10.69)	60 (9.69)	91 (8.91)
Lower gastrointestinal bleeding n (%)	138 (22.01)	201 (32.47)	303 (29.68)
Small bowel bleeding n (%)	18 (2.87)	13 (2.10)	23 (2.25)
Iatrogenic bleeding n (%)	32 (5.10)	32 (5.17)	77 (7.54)
Comorbidities			
Liver disease n (%)	154 (24.56)	136 (21.97)	217 (21.25)
Vascular disease n (%)	185 (29.51)	180 (29.08)	298 (29.19)
Thromboembolic disease n (%)	66 (10.53)	72 (11.63)	117 (11.46)
Pulmonary disease n (%)	127 (20.26)	116 (18.74)	198 (19.39)
Active malignant disease n (%)	91 (14.51)	66 (10.66)	128 (12.54)
Chronic kidney disease n (%)	220 (35.09)	184 (29.73)	336 (32.91)
Atrial fibrillation or flutter n (%)	152 (24.24)	131 (21.16)	236 (23.11)
Ischemic heart disease n (%)	122 (19.46)	106 (17.12)	187 (18.32)
Regular medication			
Antiplatelet drugs n (%)	174 (27.75)	171 (27.63)	293 (28.70)
Anticoagulant drugs n (%)	204 (32.54)	189 (20.53)	321 (31.44)
Bleeding severity			
Mean pre-endoscopy Rockall Score (SD)	4.32 (1.41)	3.87 (1.44)	4.12 (1.47)
Mean Glasgow Blatchford Score (SD)	12.11 (3.22)	7.89 (4.55)	10.70 (4.01)
Transfusion during hospitalization			
Fresh frozen plasma transfusion n (%)	114 (18.18)	51 (8.24)	118 (11.56)
Thrombocyte transfusion n (%)	30 (4.78)	20 (3.27)	35 (3.43)
Coagulation factor concentrate replacement n (%)	109 (17.38)	72 (11.63)	127 (12.44)
Laboratory parameters			
Mean admission Hb g/L (SD)	78.39 (22.41)	107.15 (26.03)	95.89 (30.95)
Mean ΔHb decrease g/L (SD)	22.20 (15.25)	21.58 (14.18)	21.58 (14.18)
Mean nadirHb g/L (SD)	64.83 (13.27)	84.88 (23.20)	79.42 (24.49)
In-hospital outcomes			
Need for surgery n (%)	24 (3.83)	17 (2.75)	32 (3.13)
Need for trans-arterial embolization n (%)	4 (0.64)	2 (0.32)	4 (0.39)
Need for intensive care unit admission n (%)	78 (12.44)	44 (7.11)	90 (8.81)
Need for repeat endoscopy n (%)	94 (14.99)	49 (7.92)	101 (9.89)
Rebleeding n (%)	51 (8.13)	29 (4.68)	54 (5.29)
Bleeding-related mortality n (%)	26 (4.15)	16 (2.58)	33 (3.23)
All-cause mortality n (%)	87 (13.88)	55 (8.89)	108 (10.58)
Composite endpoint	133 (21.21)	76 (12.28)	154 (15.08)
Mean length of hospital stay (SD)	8.78 (5.57)	7.77 (4.98)	7.83 (5.17)

SD: standard deviation, n: number of participants, Hb: hemoglobin.

**Table 2:** Association between the nadirHb and relevant outcomes in the population transfused.

NadirHb (g/L)	Univariable analysis					Multivariable analysis			
	Cases	Outcome (%)	OR (95% CI)	*p*-value		Cases	Outcome (%)	aOR (95% CI)	*p*-value
Composite endpoint									
> 80	62	11 (**18**)	0.64 (0.22;1.88)	0.668		52	8 (**15**)	0.75 (0.31;1.82)	0.798
> 70-≤80	167	26 (**15**)	0.55 (0.28;1.28)	0.240		121	16 (**13**)	0.64 (0.33;1.23)	0.259
> 60-≤70	192	43 (**23**)	**1**			141	33 (**23**)	**1**	
≤ 60	200	47 (**24**)	0.78 (0.38;1.63)	0.793		146	33 (**23**)	1.06 (0.60;1.88)	0.990
Bleeding-related mortality									
> 80	57	2 (**4**)	3.49 (0.31;39.47)	0.481		50	2 (**4**)	1.38 (0.19;10.23)	0.965
> 70-≤80	164	4 (**2**)	0.79 (0.07;8.68)	0.992		119	2 (**2**)	0.95 (0.19;4.70)	0.999
> 60-≤70	195	5 (**3**)	**1**			144	3 (**2**)	**1**	
≤ 60	210	14 (**7**)	3.30 (0.52;20.88)	0.298		150	9 (**6**)	2.71 (0.78; 10.23)	0.151
Need for urgent intervention									
> 80	62	11 (**18**)	0.73 (0.25;2.14)	0.851		52	8 (**15**)	0.87 (0.36;2.14)	0.975
> 70-≤80	177	22 (**12**)	0.58 (0.23;1.30)	0.250		121	14 (**12**)	0.61 (0.31;1.28)	0.242
> 60-≤70	192	38 (**20**)	**1**			141	30 (**21**)	**1**	
≤ 60	200	37 (**19**)	0.65 (0.30;1.41)	0.439		146	25 (**17**)	0.92 (0.50;1.69)	0.980

CI: confidence intervals, Hb: hemoglobin, OR: odds ratio, aOR: adjusted odds ratio.

## Electronic supplementary material

Supplementary Material 1

## Data Availability

We confirm that the data supporting the findings of this study are available within the article and supplementary material. Raw data were generated from the Hungarian Gastrointestinal Bleeding Registry. Data derived supporting the findings of this study are available on request from the corresponding author.
