# Supplementary material for: Hemoglobin decrease predicts untoward outcomes better than severity of anemia
Source: Sci Rep. 2024 Dec 28;14:31056. doi: 10.1038/s41598-024-82237-6 (PMC11681255; doi:10.1038/s41598-024-82237-6)
Supplement: Supplementary file 1 — Supplementary Material 1 [file 41598_2024_82237_MOESM1_ESM.docx]

**Title**

Hemoglobin decrease predicts untoward outcomes better than severity of anemia: a cohort analysis of the Hungarian Gastrointestinal Bleeding Registry

**Short title**

Hemoglobin decrease predicts untoward outcomes better than severity of anemia:

**Authors**

Brigitta Teutsch^a,b,c^, Zsolt Abonyi Tóth^b,d^, Orsolya Ferencz^b^, Nóra Vörhendi^a^, Orsolya Anna Simon^a,e^, Eszter Boros^a,f^, Dániel Pálinkás^b,g^, Levente Frim^a,e^, Edina Tari^b,f^, Patrícia Kalló^a^, Endre Botond Gagyi^b,h^, Tamás Hussein^a,i^, Szilárd Váncsa^a,b,i^, Vivien Vass^a^, Andrea Szentesi^a^, Áron Vincze^a,e^, Ferenc Izbéki^f^, Péter Hegyi^a,b,i^, Roland Hágendorn^a,e^, Imre Szabó^a,e^ Bálint Erőss^a,b,i^

**Affiliations:**

1. Institute for Translational Medicine, Medical School, University of Pécs, Pécs, Hungary
2. Centre for Translational Medicine, Semmelweis University, Budapest, Hungary
3. Department of Radiology, Medical Imaging Centre, Semmelweis University, Budapest, Hungary
4. Department of Biostatistics, University of Veterinary Medicine, Budapest, Hungary
5. First Department of Medicine, Medical School, University of Pécs, Pécs, Hungary
6. First Department of Internal Medicine, Fejér County Szent György University Teaching Hospital, Székesfehérvár, Hungary
7. Department of Gastroenterology, Central Hospital of Nothern Pest – Military Hospital, Budapest, Hungary
8. Selye János Doctoral College for Advanced Studies, Semmelweis University, Budapest, Hungary
9. Institute of Pancreatic Diseases, Semmelweis University, Budapest, Hungary

**Correspondent author:**

Brigitta Teutsch, MD

Institute for Translational Medicine, Medical School, University of Pécs, Pécs, 7624, Hungary

E-mail address: teutschbrigitta@gmail.com

Telephone number: +36 70 743 6984

# **Table S1.** The STROBE checklist for cohort analysis reporting

|  | Item No | Recommendation | Page No |
| --- | --- | --- | --- |
| **Title and abstract** | 1 | (*a*) Indicate the study’s design with a commonly used term in the title or the abstract | 1 |
|  |  | (*b*) Provide in the abstract an informative and balanced summary of what was done and what was found | 3 |
| Introduction | | |  |
| Background/rationale | 2 | Explain the scientific background and rationale for the investigation being reported | 4 |
| Objectives | 3 | State specific objectives, including any prespecified hypotheses | 4 |
| Methods | | |  |
| Study design | 4 | Present key elements of study design early in the paper | 5 |
| Setting | 5 | Describe the setting, locations, and relevant dates, including periods of recruitment, exposure, follow-up, and data collection | 5 |
| Participants | 6 | (*a*) Give the eligibility criteria, and the sources and methods of selection of participants. Describe methods of follow-up | 5 |
|  |  | (*b*) For matched studies, give matching criteria and number of exposed and unexposed |  |
| Variables | 7 | Clearly define all outcomes, exposures, predictors, potential confounders, and effect modifiers. Give diagnostic criteria, if applicable | 5-6 |
| Data sources/ measurement | 8* | For each variable of interest, give sources of data and details of methods of assessment (measurement). Describe comparability of assessment methods if there is more than one group | 5-6 |
| Bias | 9 | Describe any efforts to address potential sources of bias | 5-6 |
| Study size | 10 | Explain how the study size was arrived at | NA |
| Quantitative variables | 11 | Explain how quantitative variables were handled in the analyses. If applicable, describe which groupings were chosen and why | 5-6 |
| Statistical methods | 12 | (*a*) Describe all statistical methods, including those used to control for confounding | 6-7 |
|  |  | (*b*) Describe any methods used to examine subgroups and interactions | 6-7 |
|  |  | (*c*) Explain how missing data were addressed | 7 |
|  |  | (*d*) If applicable, explain how loss to follow-up was addressed | NA |
|  |  | (*e*) Describe any sensitivity analyses | 7 |
| Results | | |  |
| Participants | 13* | (a) Report numbers of individuals at each stage of study—eg numbers potentially eligible, examined for eligibility, confirmed eligible, included in the study, completing follow-up, and analysed | 7 |
|  |  | (b) Give reasons for non-participation at each stage | 7 |
|  |  | (c) Consider use of a flow diagram | 7 |
| Descriptive data | 14* | (a) Give characteristics of study participants (eg demographic, clinical, social) and information on exposures and potential confounders | 7-9 |
|  |  | (b) Indicate number of participants with missing data for each variable of interest | Table S2 |
|  |  | (c) Summarise follow-up time (eg, average and total amount) | NA |
| Outcome data | 15* | Report numbers of outcome events or summary measures over time | 9-13 |
| Main results | 16 | (*a*) Give unadjusted estimates and, if applicable, confounder-adjusted estimates and their precision (eg, 95% confidence interval). Make clear which confounders were adjusted for and why they were included | 9-13 |
|  |  | (*b*) Report category boundaries when continuous variables were categorized | 9-13 |
|  |  | (*c*) If relevant, consider translating estimates of relative risk into absolute risk for a meaningful time period | NA |
| Other analyses | 17 | Report other analyses done—eg analyses of subgroups and interactions, and sensitivity analyses | 9-13 |
| Discussion | | |  |
| Key results | 18 | Summarise key results with reference to study objectives |  |
| Limitations | 19 | Discuss limitations of the study, taking into account sources of potential bias or imprecision. Discuss both direction and magnitude of any potential bias |  |
| Interpretation | 20 | Give a cautious overall interpretation of results considering objectives, limitations, multiplicity of analyses, results from similar studies, and other relevant evidence |  |
| Generalisability | 21 | Discuss the generalisability (external validity) of the study results |  |
| Other information | | |  |
| Funding | 22 | Give the source of funding and the role of the funders for the present study and, if applicable, for the original study on which the present article is based | 2 |

# **Statistical analysis**

**Analysis 1.**

As in a cohort setting, multiple variables can influence the occurrence of an endpoint, we conducted a multivariable analysis to adjust for the confounders, including the following covariates: age >65 years, sex, chronic obstructive pulmonary disease, vascular disease, ischemic heart disease, heart failure, antithrombotic treatment, anticoagulant treatment, active malignant disease, fresh frozen plasma transfusion, platelet transfusion, and transfusion of coagulation factor concentrates.

# **Table S2.** Data quality table

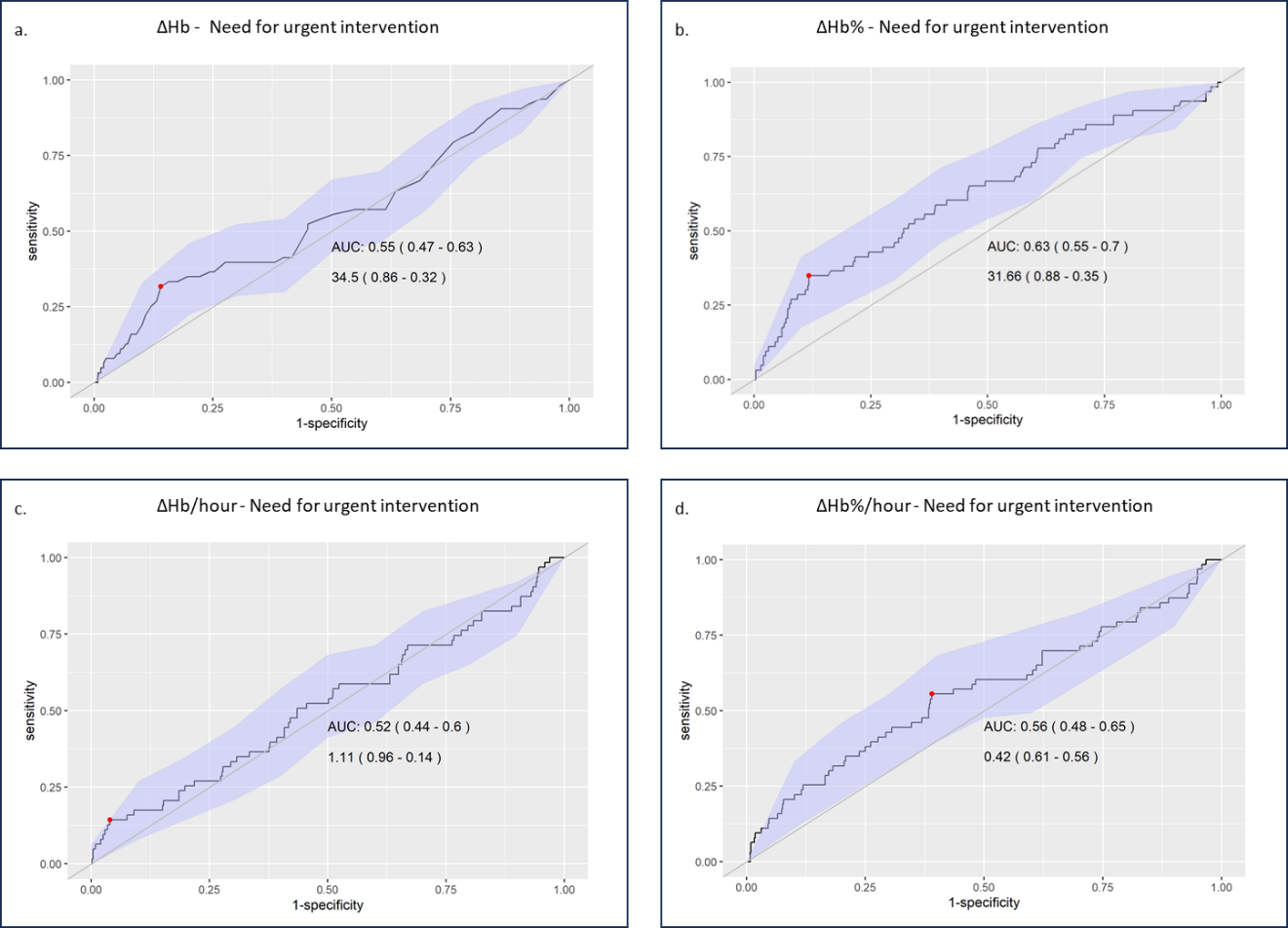


# **Figure S1**. Prediction of the bleeding-related mortality based on the ΔHb (a.), ΔHb% (b.), ΔHb/hour (c.), ΔHb%/hour (d.). Data on each figure represents the area under the curve (AUC) with 95% confidence intervals (first row) and the cut-off haemoglobin values with the related specificity and sensitivity (second row).


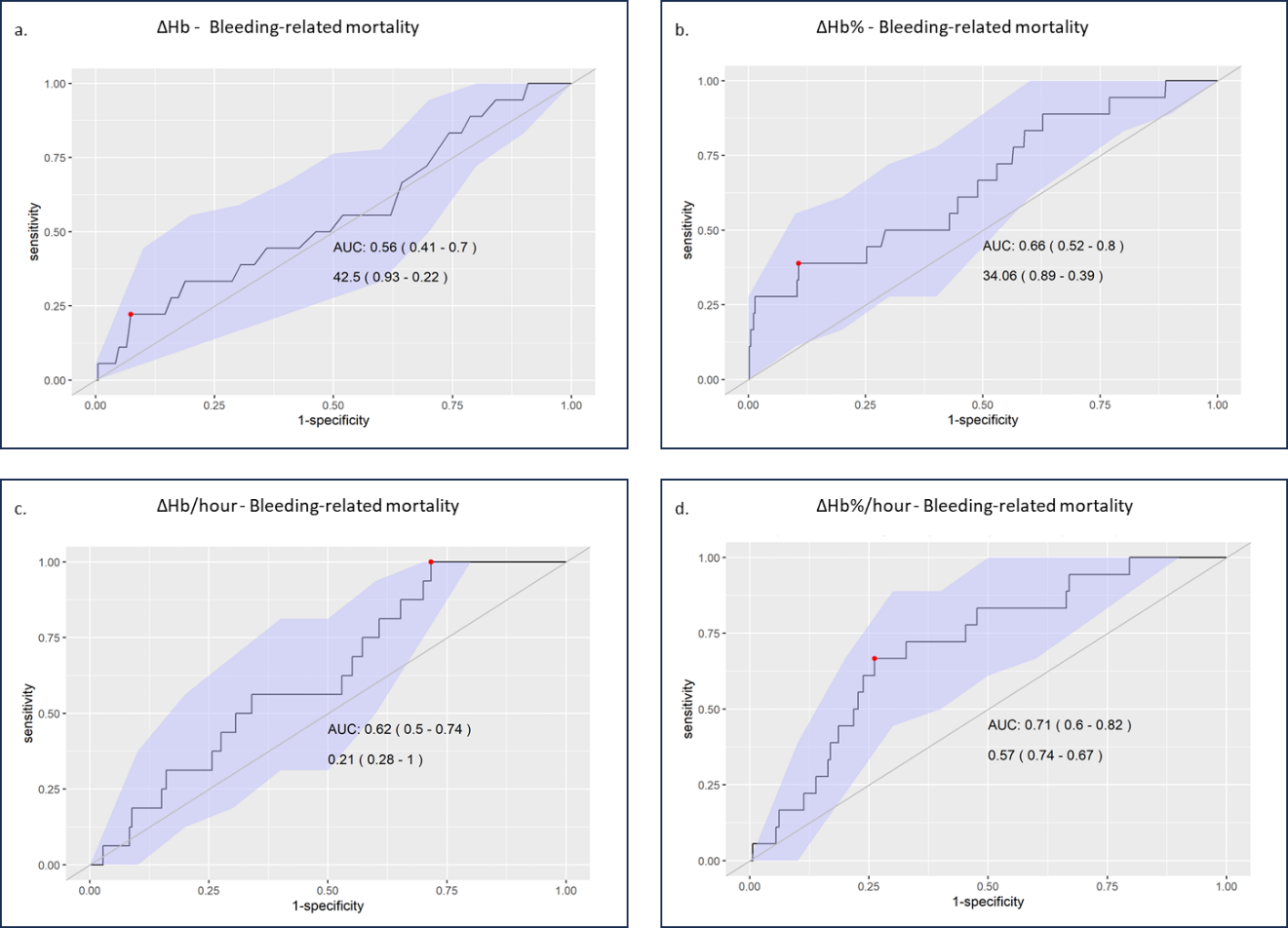


# **Figure S2**. Prediction of the need for urgent intervention based on the ΔHb (a.), ΔHb% (b.), ΔHb/hour (c.), ΔHb%/hour (d.). Data on each figure represents the area under the curve (AUC) with 95% confidence intervals (first row) and the cut-off haemoglobin values with the related specificity and sensitivity (second row).

# **Table S3.** Prediction of the composite endpoint in all participants – subgroup comparisons.

| **Comparison** | **Odds ratio** | **Standard error** | **Lower Confidence Interval** | **Upper Confidence Interval** | **Z ratio** | **P-value** |
| --- | --- | --- | --- | --- | --- | --- |
| nadirHb 50-60 deltaHb 0-10% / nadirHb 60-70 deltaHb 0-10% | 1.296145 | 0.142708 | 0.888856 | 1.890061 | 2.355946 | 0.5813 |
| nadirHb 50-60 deltaHb 0-10% / nadirHb 70-80 deltaHb 0-10% | 1.679993 | 0.369941 | 0.790065 | 3.572331 | 2.355946 | 0.5813 |
| nadirHb 50-60 deltaHb 0-10% / nadirHb 80-90 deltaHb 0-10% | 2.177514 | 0.719247 | 0.702255 | 6.751923 | 2.355946 | 0.5813 |
| nadirHb 50-60 deltaHb 0-10% / nadirHb 50-60 deltaHb 10-20% | 0.685363 | 0.089692 | 0.437725 | 1.073101 | -2.88693 | 0.2214 |
| nadirHb 50-60 deltaHb 0-10% / nadirHb 60-70 deltaHb 10-20% | 0.982685 | 0.173999 | 0.535744 | 1.802483 | -0.09865 | 1.0000 |
| nadirHb 50-60 deltaHb 0-10% / nadirHb 70-80 deltaHb 10-20% | 1.408988 | 0.339398 | 0.617303 | 3.216004 | 1.42341 | 0.9897 |
| nadirHb 50-60 deltaHb 0-10% / nadirHb 80-90 deltaHb 10-20% | 2.020229 | 0.629654 | 0.694476 | 5.876846 | 2.256237 | 0.6565 |
| nadirHb 50-60 deltaHb 0-10% / nadirHb 50-60 deltaHb 20-30% | 0.469723 | 0.122943 | 0.191603 | 1.151545 | -2.88693 | 0.2214 |
| nadirHb 50-60 deltaHb 0-10% / nadirHb 60-70 deltaHb 20-30% | 0.745032 | 0.205633 | 0.289403 | 1.91799 | -1.06639 | 0.9996 |
| nadirHb 50-60 deltaHb 0-10% / nadirHb 70-80 deltaHb 20-30% | 1.181701 | 0.370546 | 0.403593 | 3.459966 | 0.532432 | 1.0000 |
| nadirHb 50-60 deltaHb 0-10% / nadirHb 80-90 deltaHb 20-30% | 1.874305 | 0.688528 | 0.53242 | 6.598213 | 1.710183 | 0.9447 |
| nadirHb 50-60 deltaHb 0-10% / nadirHb 50-60 deltaHb 30-40% | 0.321931 | 0.126391 | 0.08387 | 1.235724 | -2.88693 | 0.2214 |
| nadirHb 50-60 deltaHb 0-10% / nadirHb 60-70 deltaHb 30-40% | 0.564853 | 0.216276 | 0.152136 | 2.097196 | -1.49179 | 0.9836 |
| nadirHb 50-60 deltaHb 0-10% / nadirHb 70-80 deltaHb 30-40% | 0.991077 | 0.407907 | 0.241943 | 4.059771 | -0.02178 | 1.0000 |
| nadirHb 50-60 deltaHb 0-10% / nadirHb 80-90 deltaHb 30-40% | 1.738921 | 0.820286 | 0.345463 | 8.753033 | 1.172865 | 0.9987 |
| nadirHb 60-70 deltaHb 0-10% / nadirHb 70-80 deltaHb 0-10% | 1.296145 | 0.142708 | 0.888856 | 1.890061 | 2.355946 | 0.5813 |
| nadirHb 60-70 deltaHb 0-10% / nadirHb 80-90 deltaHb 0-10% | 1.679993 | 0.369941 | 0.790065 | 3.572331 | 2.355946 | 0.5813 |
| nadirHb 60-70 deltaHb 0-10% / nadirHb 50-60 deltaHb 10-20% | 0.528771 | 0.06135 | 0.355331 | 0.786868 | -5.49194 | 0.0000 |
| nadirHb 60-70 deltaHb 0-10% / nadirHb 60-70 deltaHb 10-20% | 0.758159 | 0.085772 | 0.514554 | 1.117095 | -2.44726 | 0.5114 |
| nadirHb 60-70 deltaHb 0-10% / nadirHb 70-80 deltaHb 10-20% | 1.087061 | 0.170435 | 0.635289 | 1.860098 | 0.532432 | 1.0000 |
| nadirHb 60-70 deltaHb 0-10% / nadirHb 80-90 deltaHb 10-20% | 1.558644 | 0.344352 | 0.731177 | 3.322551 | 2.008852 | 0.8208 |
| nadirHb 60-70 deltaHb 0-10% / nadirHb 50-60 deltaHb 20-30% | 0.3624 | 0.080265 | 0.169685 | 0.773984 | -4.58282 | 0.0005 |
| nadirHb 60-70 deltaHb 0-10% / nadirHb 60-70 deltaHb 20-30% | 0.574806 | 0.130057 | 0.264766 | 1.247901 | -2.44726 | 0.5114 |
| nadirHb 60-70 deltaHb 0-10% / nadirHb 70-80 deltaHb 20-30% | 0.911704 | 0.23741 | 0.373592 | 2.224898 | -0.35499 | 1.0000 |
| nadirHb 60-70 deltaHb 0-10% / nadirHb 80-90 deltaHb 20-30% | 1.446061 | 0.454758 | 0.492343 | 4.247229 | 1.172865 | 0.9987 |
| nadirHb 60-70 deltaHb 0-10% / nadirHb 50-60 deltaHb 30-40% | 0.248376 | 0.085644 | 0.076217 | 0.809404 | -4.03929 | 0.0054 |
| nadirHb 60-70 deltaHb 0-10% / nadirHb 60-70 deltaHb 30-40% | 0.435794 | 0.147906 | 0.136236 | 1.394023 | -2.44726 | 0.5114 |
| nadirHb 60-70 deltaHb 0-10% / nadirHb 70-80 deltaHb 30-40% | 0.764635 | 0.28794 | 0.210449 | 2.778178 | -0.71263 | 1.0000 |
| nadirHb 60-70 deltaHb 0-10% / nadirHb 80-90 deltaHb 30-40% | 1.34161 | 0.598113 | 0.291266 | 6.17964 | 0.659172 | 1.0000 |
| nadirHb 70-80 deltaHb 0-10% / nadirHb 80-90 deltaHb 0-10% | 1.296145 | 0.142708 | 0.888856 | 1.890061 | 2.355946 | 0.5813 |
| nadirHb 70-80 deltaHb 0-10% / nadirHb 50-60 deltaHb 10-20% | 0.407956 | 0.07527 | 0.216814 | 0.767609 | -4.85947 | 0.0001 |
| nadirHb 70-80 deltaHb 0-10% / nadirHb 60-70 deltaHb 10-20% | 0.584934 | 0.079539 | 0.367098 | 0.932034 | -3.94366 | 0.0080 |
| nadirHb 70-80 deltaHb 0-10% / nadirHb 70-80 deltaHb 10-20% | 0.838687 | 0.104026 | 0.548338 | 1.28278 | -1.4183 | 0.9900 |
| nadirHb 70-80 deltaHb 0-10% / nadirHb 80-90 deltaHb 10-20% | 1.202523 | 0.189085 | 0.701671 | 2.060881 | 1.172865 | 0.9987 |
| nadirHb 70-80 deltaHb 0-10% / nadirHb 50-60 deltaHb 20-30% | 0.279598 | 0.064881 | 0.12626 | 0.619161 | -5.49194 | 0.0000 |
| nadirHb 70-80 deltaHb 0-10% / nadirHb 60-70 deltaHb 20-30% | 0.443473 | 0.099614 | 0.205422 | 0.957386 | -3.61992 | 0.0262 |
| nadirHb 70-80 deltaHb 0-10% / nadirHb 70-80 deltaHb 20-30% | 0.703396 | 0.174491 | 0.300674 | 1.645524 | -1.4183 | 0.9900 |
| nadirHb 70-80 deltaHb 0-10% / nadirHb 80-90 deltaHb 20-30% | 1.115663 | 0.329251 | 0.405906 | 3.066484 | 0.370865 | 1.0000 |
| nadirHb 70-80 deltaHb 0-10% / nadirHb 50-60 deltaHb 30-40% | 0.191626 | 0.062947 | 0.062186 | 0.590496 | -5.02973 | 0.0001 |
| nadirHb 70-80 deltaHb 0-10% / nadirHb 60-70 deltaHb 30-40% | 0.336223 | 0.110504 | 0.109046 | 1.036685 | -3.31642 | 0.0703 |
| nadirHb 70-80 deltaHb 0-10% / nadirHb 70-80 deltaHb 30-40% | 0.58993 | 0.219515 | 0.164871 | 2.110844 | -1.4183 | 0.9900 |
| nadirHb 70-80 deltaHb 0-10% / nadirHb 80-90 deltaHb 30-40% | 1.035077 | 0.46201 | 0.224305 | 4.776461 | 0.077238 | 1.0000 |
| nadirHb 80-90 deltaHb 0-10% / nadirHb 50-60 deltaHb 10-20% | 0.314746 | 0.088391 | 0.120256 | 0.823787 | -4.11629 | 0.0040 |
| nadirHb 80-90 deltaHb 0-10% / nadirHb 60-70 deltaHb 10-20% | 0.451287 | 0.099311 | 0.212335 | 0.959146 | -3.61559 | 0.0266 |
| nadirHb 80-90 deltaHb 0-10% / nadirHb 70-80 deltaHb 10-20% | 0.647063 | 0.112879 | 0.355944 | 1.17628 | -2.49536 | 0.4750 |
| nadirHb 80-90 deltaHb 0-10% / nadirHb 80-90 deltaHb 10-20% | 0.927769 | 0.146352 | 0.540416 | 1.592764 | -0.47528 | 1.0000 |
| nadirHb 80-90 deltaHb 0-10% / nadirHb 50-60 deltaHb 20-30% | 0.215715 | 0.062104 | 0.080448 | 0.578425 | -5.32758 | 0.0000 |
| nadirHb 80-90 deltaHb 0-10% / nadirHb 60-70 deltaHb 20-30% | 0.342148 | 0.09305 | 0.134761 | 0.868688 | -3.94366 | 0.0080 |
| nadirHb 80-90 deltaHb 0-10% / nadirHb 70-80 deltaHb 20-30% | 0.542683 | 0.153025 | 0.206533 | 1.425949 | -2.16765 | 0.7202 |
| nadirHb 80-90 deltaHb 0-10% / nadirHb 80-90 deltaHb 20-30% | 0.860754 | 0.271561 | 0.292049 | 2.536897 | -0.47528 | 1.0000 |
| nadirHb 80-90 deltaHb 0-10% / nadirHb 50-60 deltaHb 30-40% | 0.147843 | 0.05146 | 0.044864 | 0.487197 | -5.49194 | 0.0000 |
| nadirHb 80-90 deltaHb 0-10% / nadirHb 60-70 deltaHb 30-40% | 0.259403 | 0.091747 | 0.077219 | 0.871418 | -3.81518 | 0.0130 |
| nadirHb 80-90 deltaHb 0-10% / nadirHb 70-80 deltaHb 30-40% | 0.455142 | 0.181692 | 0.115924 | 1.786974 | -1.97182 | 0.8411 |
| nadirHb 80-90 deltaHb 0-10% / nadirHb 80-90 deltaHb 30-40% | 0.798581 | 0.377918 | 0.157828 | 4.040678 | -0.47528 | 1.0000 |
| nadirHb 50-60 deltaHb 10-20% / nadirHb 60-70 deltaHb 10-20% | 1.433815 | 0.113107 | 1.094255 | 1.878745 | 4.567875 | 0.0005 |
| nadirHb 50-60 deltaHb 10-20% / nadirHb 70-80 deltaHb 10-20% | 2.055827 | 0.32435 | 1.197395 | 3.529682 | 4.567875 | 0.0005 |
| nadirHb 50-60 deltaHb 10-20% / nadirHb 80-90 deltaHb 10-20% | 2.947676 | 0.697586 | 1.310256 | 6.631372 | 4.567875 | 0.0005 |
| nadirHb 50-60 deltaHb 10-20% / nadirHb 50-60 deltaHb 20-30% | 0.685363 | 0.089692 | 0.437725 | 1.073101 | -2.88693 | 0.2214 |
| nadirHb 50-60 deltaHb 10-20% / nadirHb 60-70 deltaHb 20-30% | 1.087061 | 0.170435 | 0.635289 | 1.860098 | 0.532432 | 1.0000 |
| nadirHb 50-60 deltaHb 10-20% / nadirHb 70-80 deltaHb 20-30% | 1.724196 | 0.371844 | 0.823569 | 3.609718 | 2.525986 | 0.4521 |
| nadirHb 50-60 deltaHb 10-20% / nadirHb 80-90 deltaHb 20-30% | 2.734761 | 0.787463 | 1.019722 | 7.334271 | 3.493866 | 0.0401 |
| nadirHb 50-60 deltaHb 10-20% / nadirHb 50-60 deltaHb 30-40% | 0.469723 | 0.122943 | 0.191603 | 1.151545 | -2.88693 | 0.2214 |
| nadirHb 50-60 deltaHb 10-20% / nadirHb 60-70 deltaHb 30-40% | 0.824165 | 0.215764 | 0.336112 | 2.0209 | -0.73868 | 1.0000 |
| nadirHb 50-60 deltaHb 10-20% / nadirHb 70-80 deltaHb 30-40% | 1.446061 | 0.454758 | 0.492343 | 4.247229 | 1.172865 | 0.9987 |
| nadirHb 50-60 deltaHb 10-20% / nadirHb 80-90 deltaHb 30-40% | 2.537225 | 1.013579 | 0.6456 | 9.971357 | 2.330689 | 0.6005 |
| nadirHb 60-70 deltaHb 10-20% / nadirHb 70-80 deltaHb 10-20% | 1.433815 | 0.113107 | 1.094255 | 1.878745 | 4.567875 | 0.0005 |
| nadirHb 60-70 deltaHb 10-20% / nadirHb 80-90 deltaHb 10-20% | 2.055827 | 0.32435 | 1.197395 | 3.529682 | 4.567875 | 0.0005 |
| nadirHb 60-70 deltaHb 10-20% / nadirHb 50-60 deltaHb 20-30% | 0.478 | 0.059215 | 0.312683 | 0.730721 | -5.95849 | 0.0000 |
| nadirHb 60-70 deltaHb 10-20% / nadirHb 60-70 deltaHb 20-30% | 0.758159 | 0.085772 | 0.514554 | 1.117095 | -2.44726 | 0.5114 |
| nadirHb 60-70 deltaHb 10-20% / nadirHb 70-80 deltaHb 20-30% | 1.202523 | 0.189085 | 0.701671 | 2.060881 | 1.172865 | 0.9987 |
| nadirHb 60-70 deltaHb 10-20% / nadirHb 80-90 deltaHb 20-30% | 1.907331 | 0.431259 | 0.879024 | 4.138583 | 2.855762 | 0.2377 |
| nadirHb 60-70 deltaHb 10-20% / nadirHb 50-60 deltaHb 30-40% | 0.327604 | 0.079388 | 0.142819 | 0.751469 | -4.60512 | 0.0005 |
| nadirHb 60-70 deltaHb 10-20% / nadirHb 60-70 deltaHb 30-40% | 0.574806 | 0.130057 | 0.264766 | 1.247901 | -2.44726 | 0.5114 |
| nadirHb 60-70 deltaHb 10-20% / nadirHb 70-80 deltaHb 30-40% | 1.008541 | 0.274344 | 0.397147 | 2.561153 | 0.031264 | 1.0000 |
| nadirHb 60-70 deltaHb 10-20% / nadirHb 80-90 deltaHb 30-40% | 1.769562 | 0.630927 | 0.521627 | 6.003044 | 1.600733 | 0.9686 |
| nadirHb 70-80 deltaHb 10-20% / nadirHb 80-90 deltaHb 10-20% | 1.433815 | 0.113107 | 1.094255 | 1.878745 | 4.567875 | 0.0005 |
| nadirHb 70-80 deltaHb 10-20% / nadirHb 50-60 deltaHb 20-30% | 0.333376 | 0.053768 | 0.191848 | 0.57931 | -6.81086 | 0.0000 |
| nadirHb 70-80 deltaHb 10-20% / nadirHb 60-70 deltaHb 20-30% | 0.528771 | 0.06135 | 0.355331 | 0.786868 | -5.49194 | 0.0000 |
| nadirHb 70-80 deltaHb 10-20% / nadirHb 70-80 deltaHb 20-30% | 0.838687 | 0.104026 | 0.548338 | 1.28278 | -1.4183 | 0.9900 |
| nadirHb 70-80 deltaHb 10-20% / nadirHb 80-90 deltaHb 20-30% | 1.330249 | 0.237146 | 0.722237 | 2.450111 | 1.600733 | 0.9686 |
| nadirHb 70-80 deltaHb 10-20% / nadirHb 50-60 deltaHb 30-40% | 0.228484 | 0.05661 | 0.09777 | 0.533954 | -5.95849 | 0.0000 |
| nadirHb 70-80 deltaHb 10-20% / nadirHb 60-70 deltaHb 30-40% | 0.400892 | 0.08626 | 0.191811 | 0.837878 | -4.2481 | 0.0023 |
| nadirHb 70-80 deltaHb 10-20% / nadirHb 70-80 deltaHb 30-40% | 0.703396 | 0.174491 | 0.300674 | 1.645524 | -1.4183 | 0.9900 |
| nadirHb 70-80 deltaHb 10-20% / nadirHb 80-90 deltaHb 30-40% | 1.234163 | 0.403906 | 0.402182 | 3.78724 | 0.64287 | 1.0000 |
| nadirHb 80-90 deltaHb 10-20% / nadirHb 50-60 deltaHb 20-30% | 0.23251 | 0.051534 | 0.108808 | 0.496846 | -6.58194 | 0.0000 |
| nadirHb 80-90 deltaHb 10-20% / nadirHb 60-70 deltaHb 20-30% | 0.368786 | 0.060114 | 0.210978 | 0.64463 | -6.11969 | 0.0000 |
| nadirHb 80-90 deltaHb 10-20% / nadirHb 70-80 deltaHb 20-30% | 0.584934 | 0.079539 | 0.367098 | 0.932034 | -3.94366 | 0.0080 |
| nadirHb 80-90 deltaHb 10-20% / nadirHb 80-90 deltaHb 20-30% | 0.927769 | 0.146352 | 0.540416 | 1.592764 | -0.47528 | 1.0000 |
| nadirHb 80-90 deltaHb 10-20% / nadirHb 50-60 deltaHb 30-40% | 0.159354 | 0.044074 | 0.061779 | 0.411041 | -6.64055 | 0.0000 |
| nadirHb 80-90 deltaHb 10-20% / nadirHb 60-70 deltaHb 30-40% | 0.279598 | 0.064881 | 0.12626 | 0.619161 | -5.49194 | 0.0000 |
| nadirHb 80-90 deltaHb 10-20% / nadirHb 70-80 deltaHb 30-40% | 0.490577 | 0.121686 | 0.209718 | 1.147565 | -2.87114 | 0.2295 |
| nadirHb 80-90 deltaHb 10-20% / nadirHb 80-90 deltaHb 30-40% | 0.860754 | 0.271561 | 0.292049 | 2.536897 | -0.47528 | 1.0000 |
| nadirHb 50-60 deltaHb 20-30% / nadirHb 60-70 deltaHb 20-30% | 1.586108 | 0.134929 | 1.185106 | 2.122797 | 5.422433 | 0.0000 |
| nadirHb 50-60 deltaHb 20-30% / nadirHb 70-80 deltaHb 20-30% | 2.515739 | 0.428025 | 1.404475 | 4.506269 | 5.422433 | 0.0000 |
| nadirHb 50-60 deltaHb 20-30% / nadirHb 80-90 deltaHb 20-30% | 3.990234 | 1.018341 | 1.664452 | 9.565895 | 5.422433 | 0.0000 |
| nadirHb 50-60 deltaHb 20-30% / nadirHb 50-60 deltaHb 30-40% | 0.685363 | 0.089692 | 0.437725 | 1.073101 | -2.88693 | 0.2214 |
| nadirHb 50-60 deltaHb 20-30% / nadirHb 60-70 deltaHb 30-40% | 1.202523 | 0.189085 | 0.701671 | 2.060881 | 1.172865 | 0.9987 |
| nadirHb 50-60 deltaHb 20-30% / nadirHb 70-80 deltaHb 30-40% | 2.109918 | 0.528116 | 0.895033 | 4.973846 | 2.982997 | 0.1759 |
| nadirHb 50-60 deltaHb 20-30% / nadirHb 80-90 deltaHb 30-40% | 3.702014 | 1.339422 | 1.071763 | 12.78726 | 3.617592 | 0.0264 |
| nadirHb 60-70 deltaHb 20-30% / nadirHb 70-80 deltaHb 20-30% | 1.586108 | 0.134929 | 1.185106 | 2.122797 | 5.422433 | 0.0000 |
| nadirHb 60-70 deltaHb 20-30% / nadirHb 80-90 deltaHb 20-30% | 2.515739 | 0.428025 | 1.404475 | 4.506269 | 5.422433 | 0.0000 |
| nadirHb 60-70 deltaHb 20-30% / nadirHb 50-60 deltaHb 30-40% | 0.432104 | 0.067143 | 0.253739 | 0.735849 | -5.40003 | 0.0000 |
| nadirHb 60-70 deltaHb 20-30% / nadirHb 60-70 deltaHb 30-40% | 0.758159 | 0.085772 | 0.514554 | 1.117095 | -2.44726 | 0.5114 |
| nadirHb 60-70 deltaHb 20-30% / nadirHb 70-80 deltaHb 30-40% | 1.330249 | 0.237146 | 0.722237 | 2.450111 | 1.600733 | 0.9686 |
| nadirHb 60-70 deltaHb 20-30% / nadirHb 80-90 deltaHb 30-40% | 2.334024 | 0.664649 | 0.879831 | 6.191715 | 2.976461 | 0.1788 |
| nadirHb 70-80 deltaHb 20-30% / nadirHb 80-90 deltaHb 20-30% | 1.586108 | 0.134929 | 1.185106 | 2.122797 | 5.422433 | 0.0000 |
| nadirHb 70-80 deltaHb 20-30% / nadirHb 50-60 deltaHb 30-40% | 0.27243 | 0.058199 | 0.131038 | 0.566389 | -6.08709 | 0.0000 |
| nadirHb 70-80 deltaHb 20-30% / nadirHb 60-70 deltaHb 30-40% | 0.478 | 0.059215 | 0.312683 | 0.730721 | -5.95849 | 0.0000 |
| nadirHb 70-80 deltaHb 20-30% / nadirHb 70-80 deltaHb 30-40% | 0.838687 | 0.104026 | 0.548338 | 1.28278 | -1.4183 | 0.9900 |
| nadirHb 70-80 deltaHb 20-30% / nadirHb 80-90 deltaHb 30-40% | 1.471541 | 0.314754 | 0.707158 | 3.062162 | 1.80608 | 0.9148 |
| nadirHb 80-90 deltaHb 20-30% / nadirHb 50-60 deltaHb 30-40% | 0.17176 | 0.049065 | 0.064548 | 0.457048 | -6.16693 | 0.0000 |
| nadirHb 80-90 deltaHb 20-30% / nadirHb 60-70 deltaHb 30-40% | 0.301366 | 0.054219 | 0.162706 | 0.558196 | -6.66678 | 0.0000 |
| nadirHb 80-90 deltaHb 20-30% / nadirHb 70-80 deltaHb 30-40% | 0.528771 | 0.06135 | 0.355331 | 0.786868 | -5.49194 | 0.0000 |
| nadirHb 80-90 deltaHb 20-30% / nadirHb 80-90 deltaHb 30-40% | 0.927769 | 0.146352 | 0.540416 | 1.592764 | -0.47528 | 1.0000 |
| nadirHb 50-60 deltaHb 30-40% / nadirHb 60-70 deltaHb 30-40% | 1.754577 | 0.216063 | 1.15066 | 2.675456 | 4.565656 | 0.0006 |
| nadirHb 50-60 deltaHb 30-40% / nadirHb 70-80 deltaHb 30-40% | 3.078539 | 0.7582 | 1.324017 | 7.158067 | 4.565656 | 0.0006 |
| nadirHb 50-60 deltaHb 30-40% / nadirHb 80-90 deltaHb 30-40% | 5.401533 | 1.99548 | 1.523493 | 19.1511 | 4.565656 | 0.0006 |
| nadirHb 60-70 deltaHb 30-40% / nadirHb 70-80 deltaHb 30-40% | 1.754577 | 0.216063 | 1.15066 | 2.675456 | 4.565656 | 0.0006 |
| nadirHb 60-70 deltaHb 30-40% / nadirHb 80-90 deltaHb 30-40% | 3.078539 | 0.7582 | 1.324017 | 7.158067 | 4.565656 | 0.0006 |
| nadirHb 70-80 deltaHb 30-40% / nadirHb 80-90 deltaHb 30-40% | 1.754577 | 0.216063 | 1.15066 | 2.675456 | 4.565656 | 0.0006 |

# **Table S4.** Prediction of the composite endpoint in the red blood cell transfused population – subgroup comparisons.

| **Comparison** | **Odds ratio** | **Standard error** | **Lower Confidence Interval** | **Upper Confidence Interval** | **Z ratio** | **P-value** |
| --- | --- | --- | --- | --- | --- | --- |
| nadirHb 50-60 deltaHb 0-10% / nadirHb 60-70 deltaHb 0-10% | 1.009369 | 0.241574 | 0.444576 | 2.291682 | 0.038964 | 1.0000 |
| nadirHb 50-60 deltaHb 0-10% / nadirHb 70-80 deltaHb 0-10% | 1.018826 | 0.487675 | 0.197647 | 5.251807 | 0.038964 | 1.0000 |
| nadirHb 50-60 deltaHb 0-10% / nadirHb 80-90 deltaHb 0-10% | 1.028371 | 0.738366 | 0.087869 | 12.03547 | 0.038964 | 1.0000 |
| nadirHb 50-60 deltaHb 0-10% / nadirHb 50-60 deltaHb 10-20% | 0.634885 | 0.093736 | 0.382838 | 1.05287 | -3.0771 | 0.1383 |
| nadirHb 50-60 deltaHb 0-10% / nadirHb 60-70 deltaHb 10-20% | 0.768285 | 0.207225 | 0.304928 | 1.935743 | -0.97728 | 0.9999 |
| nadirHb 50-60 deltaHb 0-10% / nadirHb 70-80 deltaHb 10-20% | 0.929715 | 0.39222 | 0.2191 | 3.945093 | -0.17275 | 1.0000 |
| nadirHb 50-60 deltaHb 0-10% / nadirHb 80-90 deltaHb 10-20% | 1.125064 | 0.653576 | 0.15375 | 8.232661 | 0.202848 | 1.0000 |
| nadirHb 50-60 deltaHb 0-10% / nadirHb 50-60 deltaHb 20-30% | 0.403079 | 0.119024 | 0.146565 | 1.108535 | -3.0771 | 0.1383 |
| nadirHb 50-60 deltaHb 0-10% / nadirHb 60-70 deltaHb 20-30% | 0.584783 | 0.202147 | 0.178918 | 1.911327 | -1.55206 | 0.9763 |
| nadirHb 50-60 deltaHb 0-10% / nadirHb 70-80 deltaHb 20-30% | 0.848398 | 0.367277 | 0.192519 | 3.738745 | -0.37977 | 1.0000 |
| nadirHb 50-60 deltaHb 0-10% / nadirHb 80-90 deltaHb 20-30% | 1.230847 | 0.663899 | 0.193932 | 7.811953 | 0.385074 | 1.0000 |
| nadirHb 50-60 deltaHb 0-10% / nadirHb 50-60 deltaHb 30-40% | 0.255908 | 0.113349 | 0.056111 | 1.167143 | -3.0771 | 0.1383 |
| nadirHb 50-60 deltaHb 0-10% / nadirHb 60-70 deltaHb 30-40% | 0.44511 | 0.197816 | 0.097098 | 2.040446 | -1.82133 | 0.9092 |
| nadirHb 50-60 deltaHb 0-10% / nadirHb 70-80 deltaHb 30-40% | 0.774193 | 0.392796 | 0.136126 | 4.403095 | -0.50444 | 1.0000 |
| nadirHb 50-60 deltaHb 0-10% / nadirHb 80-90 deltaHb 30-40% | 1.346578 | 0.825601 | 0.164808 | 11.00235 | 0.485339 | 1.0000 |
| nadirHb 60-70 deltaHb 0-10% / nadirHb 70-80 deltaHb 0-10% | 1.009369 | 0.241574 | 0.444576 | 2.291682 | 0.038964 | 1.0000 |
| nadirHb 60-70 deltaHb 0-10% / nadirHb 80-90 deltaHb 0-10% | 1.018826 | 0.487675 | 0.197647 | 5.251807 | 0.038964 | 1.0000 |
| nadirHb 60-70 deltaHb 0-10% / nadirHb 50-60 deltaHb 10-20% | 0.628992 | 0.123725 | 0.320603 | 1.234019 | -2.35704 | 0.5804 |
| nadirHb 60-70 deltaHb 0-10% / nadirHb 60-70 deltaHb 10-20% | 0.761154 | 0.095178 | 0.495927 | 1.168225 | -2.18259 | 0.7097 |
| nadirHb 60-70 deltaHb 0-10% / nadirHb 70-80 deltaHb 10-20% | 0.921085 | 0.199372 | 0.43877 | 1.933583 | -0.37977 | 1.0000 |
| nadirHb 60-70 deltaHb 0-10% / nadirHb 80-90 deltaHb 10-20% | 1.114621 | 0.405483 | 0.320513 | 3.876218 | 0.298291 | 1.0000 |
| nadirHb 60-70 deltaHb 0-10% / nadirHb 50-60 deltaHb 20-30% | 0.399337 | 0.100789 | 0.168191 | 0.948148 | -3.637 | 0.0247 |
| nadirHb 60-70 deltaHb 0-10% / nadirHb 60-70 deltaHb 20-30% | 0.579355 | 0.14489 | 0.245944 | 1.36475 | -2.18259 | 0.7097 |
| nadirHb 60-70 deltaHb 0-10% / nadirHb 70-80 deltaHb 20-30% | 0.840523 | 0.261779 | 0.289163 | 2.443183 | -0.55782 | 1.0000 |
| nadirHb 60-70 deltaHb 0-10% / nadirHb 80-90 deltaHb 20-30% | 1.219423 | 0.498427 | 0.300597 | 4.946792 | 0.485339 | 1.0000 |
| nadirHb 60-70 deltaHb 0-10% / nadirHb 50-60 deltaHb 30-40% | 0.253533 | 0.092221 | 0.072915 | 0.881555 | -3.77263 | 0.0152 |
| nadirHb 60-70 deltaHb 0-10% / nadirHb 60-70 deltaHb 30-40% | 0.440978 | 0.165425 | 0.12197 | 1.594336 | -2.18259 | 0.7097 |
| nadirHb 60-70 deltaHb 0-10% / nadirHb 70-80 deltaHb 30-40% | 0.767007 | 0.349576 | 0.160938 | 3.655432 | -0.58201 | 1.0000 |
| nadirHb 60-70 deltaHb 0-10% / nadirHb 80-90 deltaHb 30-40% | 1.334079 | 0.770197 | 0.184575 | 9.642506 | 0.49927 | 1.0000 |
| nadirHb 70-80 deltaHb 0-10% / nadirHb 80-90 deltaHb 0-10% | 1.009369 | 0.241574 | 0.444576 | 2.291682 | 0.038964 | 1.0000 |
| nadirHb 70-80 deltaHb 0-10% / nadirHb 50-60 deltaHb 10-20% | 0.623153 | 0.257042 | 0.151653 | 2.56058 | -1.14661 | 0.9990 |
| nadirHb 70-80 deltaHb 0-10% / nadirHb 60-70 deltaHb 10-20% | 0.754089 | 0.203856 | 0.298669 | 1.903948 | -1.04406 | 0.9997 |
| nadirHb 70-80 deltaHb 0-10% / nadirHb 70-80 deltaHb 10-20% | 0.912535 | 0.158812 | 0.502693 | 1.656518 | -0.52592 | 1.0000 |
| nadirHb 70-80 deltaHb 0-10% / nadirHb 80-90 deltaHb 10-20% | 1.104275 | 0.225681 | 0.548267 | 2.224138 | 0.485339 | 1.0000 |
| nadirHb 70-80 deltaHb 0-10% / nadirHb 50-60 deltaHb 20-30% | 0.395631 | 0.155644 | 0.102787 | 1.522802 | -2.35704 | 0.5804 |
| nadirHb 70-80 deltaHb 0-10% / nadirHb 60-70 deltaHb 20-30% | 0.573977 | 0.198958 | 0.175041 | 1.882135 | -1.6016 | 0.9685 |
| nadirHb 70-80 deltaHb 0-10% / nadirHb 70-80 deltaHb 20-30% | 0.832721 | 0.289843 | 0.252701 | 2.744053 | -0.52592 | 1.0000 |
| nadirHb 70-80 deltaHb 0-10% / nadirHb 80-90 deltaHb 20-30% | 1.208104 | 0.47985 | 0.309826 | 4.710763 | 0.475971 | 1.0000 |
| nadirHb 70-80 deltaHb 0-10% / nadirHb 50-60 deltaHb 30-40% | 0.25118 | 0.107448 | 0.058009 | 1.087605 | -3.22973 | 0.0907 |
| nadirHb 70-80 deltaHb 0-10% / nadirHb 60-70 deltaHb 30-40% | 0.436885 | 0.194646 | 0.094942 | 2.010375 | -1.85865 | 0.8945 |
| nadirHb 70-80 deltaHb 0-10% / nadirHb 70-80 deltaHb 30-40% | 0.759887 | 0.396738 | 0.127031 | 4.545574 | -0.52592 | 1.0000 |
| nadirHb 70-80 deltaHb 0-10% / nadirHb 80-90 deltaHb 30-40% | 1.321696 | 0.841396 | 0.149253 | 11.70416 | 0.438131 | 1.0000 |
| nadirHb 80-90 deltaHb 0-10% / nadirHb 50-60 deltaHb 10-20% | 0.617369 | 0.398267 | 0.067716 | 5.628604 | -0.74761 | 1.0000 |
| nadirHb 80-90 deltaHb 0-10% / nadirHb 60-70 deltaHb 10-20% | 0.747089 | 0.369854 | 0.137015 | 4.07359 | -0.58896 | 1.0000 |
| nadirHb 80-90 deltaHb 0-10% / nadirHb 70-80 deltaHb 10-20% | 0.904065 | 0.323805 | 0.265021 | 3.084031 | -0.28158 | 1.0000 |
| nadirHb 80-90 deltaHb 0-10% / nadirHb 80-90 deltaHb 10-20% | 1.094025 | 0.280535 | 0.454454 | 2.633687 | 0.350448 | 1.0000 |
| nadirHb 80-90 deltaHb 0-10% / nadirHb 50-60 deltaHb 20-30% | 0.391958 | 0.235304 | 0.050119 | 3.065322 | -1.56014 | 0.9751 |
| nadirHb 80-90 deltaHb 0-10% / nadirHb 60-70 deltaHb 20-30% | 0.56865 | 0.30745 | 0.089203 | 3.625017 | -1.04406 | 0.9997 |
| nadirHb 80-90 deltaHb 0-10% / nadirHb 70-80 deltaHb 20-30% | 0.824992 | 0.420553 | 0.143868 | 4.730813 | -0.37739 | 1.0000 |
| nadirHb 80-90 deltaHb 0-10% / nadirHb 80-90 deltaHb 20-30% | 1.19689 | 0.613824 | 0.206529 | 6.936306 | 0.350448 | 1.0000 |
| nadirHb 80-90 deltaHb 0-10% / nadirHb 50-60 deltaHb 30-40% | 0.248848 | 0.146848 | 0.032954 | 1.879166 | -2.35704 | 0.5804 |
| nadirHb 80-90 deltaHb 0-10% / nadirHb 60-70 deltaHb 30-40% | 0.43283 | 0.263575 | 0.053733 | 3.486531 | -1.37515 | 0.9927 |
| nadirHb 80-90 deltaHb 0-10% / nadirHb 70-80 deltaHb 30-40% | 0.752834 | 0.506146 | 0.075222 | 7.534468 | -0.42228 | 1.0000 |
| nadirHb 80-90 deltaHb 0-10% / nadirHb 80-90 deltaHb 30-40% | 1.309428 | 1.007308 | 0.093858 | 18.26806 | 0.350448 | 1.0000 |
| nadirHb 50-60 deltaHb 10-20% / nadirHb 60-70 deltaHb 10-20% | 1.210117 | 0.199343 | 0.688214 | 2.127803 | 1.157753 | 0.9989 |
| nadirHb 50-60 deltaHb 10-20% / nadirHb 70-80 deltaHb 10-20% | 1.464383 | 0.482457 | 0.473638 | 4.527547 | 1.157753 | 0.9989 |
| nadirHb 50-60 deltaHb 10-20% / nadirHb 80-90 deltaHb 10-20% | 1.772075 | 0.875744 | 0.325964 | 9.633729 | 1.157753 | 0.9989 |
| nadirHb 50-60 deltaHb 10-20% / nadirHb 50-60 deltaHb 20-30% | 0.634885 | 0.093736 | 0.382838 | 1.05287 | -3.0771 | 0.1383 |
| nadirHb 50-60 deltaHb 10-20% / nadirHb 60-70 deltaHb 20-30% | 0.921085 | 0.199372 | 0.43877 | 1.933583 | -0.37977 | 1.0000 |
| nadirHb 50-60 deltaHb 10-20% / nadirHb 70-80 deltaHb 20-30% | 1.336302 | 0.438174 | 0.434528 | 4.109524 | 0.884127 | 1.0000 |
| nadirHb 50-60 deltaHb 10-20% / nadirHb 80-90 deltaHb 20-30% | 1.938694 | 0.875206 | 0.412862 | 9.103603 | 1.466448 | 0.9861 |
| nadirHb 50-60 deltaHb 10-20% / nadirHb 50-60 deltaHb 30-40% | 0.403079 | 0.119024 | 0.146565 | 1.108535 | -3.0771 | 0.1383 |
| nadirHb 50-60 deltaHb 10-20% / nadirHb 60-70 deltaHb 30-40% | 0.701087 | 0.219296 | 0.240084 | 2.047299 | -1.13533 | 0.9991 |
| nadirHb 50-60 deltaHb 10-20% / nadirHb 70-80 deltaHb 30-40% | 1.219423 | 0.498427 | 0.300597 | 4.946792 | 0.485339 | 1.0000 |
| nadirHb 50-60 deltaHb 10-20% / nadirHb 80-90 deltaHb 30-40% | 2.12098 | 1.151753 | 0.330034 | 13.6306 | 1.384601 | 0.9922 |
| nadirHb 60-70 deltaHb 10-20% / nadirHb 70-80 deltaHb 10-20% | 1.210117 | 0.199343 | 0.688214 | 2.127803 | 1.157753 | 0.9989 |
| nadirHb 60-70 deltaHb 10-20% / nadirHb 80-90 deltaHb 10-20% | 1.464383 | 0.482457 | 0.473638 | 4.527547 | 1.157753 | 0.9989 |
| nadirHb 60-70 deltaHb 10-20% / nadirHb 50-60 deltaHb 20-30% | 0.524647 | 0.08315 | 0.304827 | 0.902988 | -4.06992 | 0.0048 |
| nadirHb 60-70 deltaHb 10-20% / nadirHb 60-70 deltaHb 20-30% | 0.761154 | 0.095178 | 0.495927 | 1.168225 | -2.18259 | 0.7097 |
| nadirHb 60-70 deltaHb 10-20% / nadirHb 70-80 deltaHb 20-30% | 1.104275 | 0.225681 | 0.548267 | 2.224138 | 0.485339 | 1.0000 |
| nadirHb 60-70 deltaHb 10-20% / nadirHb 80-90 deltaHb 20-30% | 1.602072 | 0.515438 | 0.532075 | 4.823818 | 1.464875 | 0.9863 |
| nadirHb 60-70 deltaHb 10-20% / nadirHb 50-60 deltaHb 30-40% | 0.333091 | 0.086023 | 0.137498 | 0.806913 | -4.25676 | 0.0022 |
| nadirHb 60-70 deltaHb 10-20% / nadirHb 60-70 deltaHb 30-40% | 0.579355 | 0.14489 | 0.245944 | 1.36475 | -2.18259 | 0.7097 |
| nadirHb 60-70 deltaHb 10-20% / nadirHb 70-80 deltaHb 30-40% | 1.00769 | 0.344644 | 0.312205 | 3.252471 | 0.022398 | 1.0000 |
| nadirHb 60-70 deltaHb 10-20% / nadirHb 80-90 deltaHb 30-40% | 1.752706 | 0.840543 | 0.338964 | 9.062846 | 1.170137 | 0.9988 |
| nadirHb 70-80 deltaHb 10-20% / nadirHb 80-90 deltaHb 10-20% | 1.210117 | 0.199343 | 0.688214 | 2.127803 | 1.157753 | 0.9989 |
| nadirHb 70-80 deltaHb 10-20% / nadirHb 50-60 deltaHb 20-30% | 0.433551 | 0.124686 | 0.161855 | 1.161324 | -2.906 | 0.2118 |
| nadirHb 70-80 deltaHb 10-20% / nadirHb 60-70 deltaHb 20-30% | 0.628992 | 0.123725 | 0.320603 | 1.234019 | -2.35704 | 0.5804 |
| nadirHb 70-80 deltaHb 10-20% / nadirHb 70-80 deltaHb 20-30% | 0.912535 | 0.158812 | 0.502693 | 1.656518 | -0.52592 | 1.0000 |
| nadirHb 70-80 deltaHb 10-20% / nadirHb 80-90 deltaHb 20-30% | 1.323898 | 0.31745 | 0.582206 | 3.010456 | 1.170137 | 0.9988 |
| nadirHb 70-80 deltaHb 10-20% / nadirHb 50-60 deltaHb 30-40% | 0.275255 | 0.087249 | 0.092919 | 0.815387 | -4.06992 | 0.0048 |
| nadirHb 70-80 deltaHb 10-20% / nadirHb 60-70 deltaHb 30-40% | 0.478759 | 0.136694 | 0.180008 | 1.273332 | -2.57973 | 0.4128 |
| nadirHb 70-80 deltaHb 10-20% / nadirHb 70-80 deltaHb 30-40% | 0.832721 | 0.289843 | 0.252701 | 2.744053 | -0.52592 | 1.0000 |
| nadirHb 70-80 deltaHb 10-20% / nadirHb 80-90 deltaHb 30-40% | 1.448377 | 0.678367 | 0.291071 | 7.207166 | 0.790933 | 1.0000 |
| nadirHb 80-90 deltaHb 10-20% / nadirHb 50-60 deltaHb 20-30% | 0.358272 | 0.158036 | 0.079047 | 1.623821 | -2.32703 | 0.6033 |
| nadirHb 80-90 deltaHb 10-20% / nadirHb 60-70 deltaHb 20-30% | 0.519778 | 0.177045 | 0.161811 | 1.669654 | -1.92108 | 0.8666 |
| nadirHb 80-90 deltaHb 10-20% / nadirHb 70-80 deltaHb 20-30% | 0.754089 | 0.203856 | 0.298669 | 1.903948 | -1.04406 | 0.9997 |
| nadirHb 80-90 deltaHb 10-20% / nadirHb 80-90 deltaHb 20-30% | 1.094025 | 0.280535 | 0.454454 | 2.633687 | 0.350448 | 1.0000 |
| nadirHb 80-90 deltaHb 10-20% / nadirHb 50-60 deltaHb 30-40% | 0.227461 | 0.098761 | 0.051389 | 1.006797 | -3.41044 | 0.0525 |
| nadirHb 80-90 deltaHb 10-20% / nadirHb 60-70 deltaHb 30-40% | 0.395631 | 0.155644 | 0.102787 | 1.522802 | -2.35704 | 0.5804 |
| nadirHb 80-90 deltaHb 10-20% / nadirHb 70-80 deltaHb 30-40% | 0.688133 | 0.291625 | 0.161104 | 2.939257 | -0.88197 | 1.0000 |
| nadirHb 80-90 deltaHb 10-20% / nadirHb 80-90 deltaHb 30-40% | 1.19689 | 0.613824 | 0.206529 | 6.936306 | 0.350448 | 1.0000 |
| nadirHb 50-60 deltaHb 20-30% / nadirHb 60-70 deltaHb 20-30% | 1.450791 | 0.193596 | 0.91845 | 2.29168 | 2.788553 | 0.2754 |
| nadirHb 50-60 deltaHb 20-30% / nadirHb 70-80 deltaHb 20-30% | 2.104794 | 0.561734 | 0.843551 | 5.251796 | 2.788553 | 0.2754 |
| nadirHb 50-60 deltaHb 20-30% / nadirHb 80-90 deltaHb 20-30% | 3.053616 | 1.222438 | 0.77476 | 12.03543 | 2.788553 | 0.2754 |
| nadirHb 50-60 deltaHb 20-30% / nadirHb 50-60 deltaHb 30-40% | 0.634885 | 0.093736 | 0.382838 | 1.05287 | -3.0771 | 0.1383 |
| nadirHb 50-60 deltaHb 20-30% / nadirHb 60-70 deltaHb 30-40% | 1.104275 | 0.225681 | 0.548267 | 2.224138 | 0.485339 | 1.0000 |
| nadirHb 50-60 deltaHb 20-30% / nadirHb 70-80 deltaHb 30-40% | 1.920699 | 0.666233 | 0.585259 | 6.30334 | 1.881655 | 0.8847 |
| nadirHb 50-60 deltaHb 20-30% / nadirHb 80-90 deltaHb 30-40% | 3.340732 | 1.695081 | 0.587325 | 19.00224 | 2.377206 | 0.5650 |
| nadirHb 60-70 deltaHb 20-30% / nadirHb 70-80 deltaHb 20-30% | 1.450791 | 0.193596 | 0.91845 | 2.29168 | 2.788553 | 0.2754 |
| nadirHb 60-70 deltaHb 20-30% / nadirHb 80-90 deltaHb 20-30% | 2.104794 | 0.561734 | 0.843551 | 5.251796 | 2.788553 | 0.2754 |
| nadirHb 60-70 deltaHb 20-30% / nadirHb 50-60 deltaHb 30-40% | 0.437613 | 0.07872 | 0.236286 | 0.810478 | -4.59419 | 0.0005 |
| nadirHb 60-70 deltaHb 20-30% / nadirHb 60-70 deltaHb 30-40% | 0.761154 | 0.095178 | 0.495927 | 1.168225 | -2.18259 | 0.7097 |
| nadirHb 60-70 deltaHb 20-30% / nadirHb 70-80 deltaHb 30-40% | 1.323898 | 0.31745 | 0.582206 | 3.010456 | 1.170137 | 0.9988 |
| nadirHb 60-70 deltaHb 20-30% / nadirHb 80-90 deltaHb 30-40% | 2.302697 | 0.91513 | 0.590088 | 8.985806 | 2.098758 | 0.7663 |
| nadirHb 70-80 deltaHb 20-30% / nadirHb 80-90 deltaHb 20-30% | 1.450791 | 0.193596 | 0.91845 | 2.29168 | 2.788553 | 0.2754 |
| nadirHb 70-80 deltaHb 20-30% / nadirHb 50-60 deltaHb 30-40% | 0.301637 | 0.084529 | 0.115484 | 0.78786 | -4.27688 | 0.0020 |
| nadirHb 70-80 deltaHb 20-30% / nadirHb 60-70 deltaHb 30-40% | 0.524647 | 0.08315 | 0.304827 | 0.902988 | -4.06992 | 0.0048 |
| nadirHb 70-80 deltaHb 20-30% / nadirHb 70-80 deltaHb 30-40% | 0.912535 | 0.158812 | 0.502693 | 1.656518 | -0.52592 | 1.0000 |
| nadirHb 70-80 deltaHb 20-30% / nadirHb 80-90 deltaHb 30-40% | 1.587201 | 0.48673 | 0.555073 | 4.538517 | 1.506466 | 0.9820 |
| nadirHb 80-90 deltaHb 20-30% / nadirHb 50-60 deltaHb 30-40% | 0.207912 | 0.083247 | 0.052738 | 0.819663 | -3.92271 | 0.0086 |
| nadirHb 80-90 deltaHb 20-30% / nadirHb 60-70 deltaHb 30-40% | 0.361629 | 0.095824 | 0.145881 | 0.896451 | -3.83857 | 0.0119 |
| nadirHb 80-90 deltaHb 20-30% / nadirHb 70-80 deltaHb 30-40% | 0.628992 | 0.123725 | 0.320603 | 1.234019 | -2.35704 | 0.5804 |
| nadirHb 80-90 deltaHb 20-30% / nadirHb 80-90 deltaHb 30-40% | 1.094025 | 0.280535 | 0.454454 | 2.633687 | 0.350448 | 1.0000 |
| nadirHb 50-60 deltaHb 30-40% / nadirHb 60-70 deltaHb 30-40% | 1.739331 | 0.297682 | 0.967677 | 3.126325 | 3.234061 | 0.0896 |
| nadirHb 50-60 deltaHb 30-40% / nadirHb 70-80 deltaHb 30-40% | 3.025272 | 1.035534 | 0.936399 | 9.773906 | 3.234061 | 0.0896 |
| nadirHb 50-60 deltaHb 30-40% / nadirHb 80-90 deltaHb 30-40% | 5.26195 | 2.701705 | 0.906131 | 30.5564 | 3.234061 | 0.0896 |
| nadirHb 60-70 deltaHb 30-40% / nadirHb 70-80 deltaHb 30-40% | 1.739331 | 0.297682 | 0.967677 | 3.126325 | 3.234061 | 0.0896 |
| nadirHb 60-70 deltaHb 30-40% / nadirHb 80-90 deltaHb 30-40% | 3.025272 | 1.035534 | 0.936399 | 9.773906 | 3.234061 | 0.0896 |
| nadirHb 70-80 deltaHb 30-40% / nadirHb 80-90 deltaHb 30-40% | 1.739331 | 0.297682 | 0.967677 | 3.126325 | 3.234061 | 0.0896 |

# **Table S5.** Prediction of the bleeding-related mortality in all participants – subgroup comparisons.

| **Comparison** | **Odds ratio** | **Standard error** | **Lower Confidence Interval** | **Upper Confidence Interval** | **Z ratio** | **P-value** |
| --- | --- | --- | --- | --- | --- | --- |
| nadirHb 50-60 deltaHb 0-10% / nadirHb 60-70 deltaHb 0-10% | 1.331674 | 0.290078 | 0.631374 | 2.808725 | 1.314956 | 0.9955 |
| nadirHb 50-60 deltaHb 0-10% / nadirHb 70-80 deltaHb 0-10% | 1.773355 | 0.772579 | 0.398633 | 7.888934 | 1.314956 | 0.9955 |
| nadirHb 50-60 deltaHb 0-10% / nadirHb 80-90 deltaHb 0-10% | 2.36153 | 1.543235 | 0.251686 | 22.15784 | 1.314956 | 0.9955 |
| nadirHb 50-60 deltaHb 0-10% / nadirHb 50-60 deltaHb 10-20% | 0.754166 | 0.149923 | 0.381663 | 1.490232 | -1.41927 | 0.9900 |
| nadirHb 50-60 deltaHb 0-10% / nadirHb 60-70 deltaHb 10-20% | 1.159158 | 0.360452 | 0.39945 | 3.36375 | 0.474962 | 1.0000 |
| nadirHb 50-60 deltaHb 0-10% / nadirHb 70-80 deltaHb 10-20% | 1.781634 | 0.812875 | 0.373212 | 8.505138 | 1.265815 | 0.9970 |
| nadirHb 50-60 deltaHb 0-10% / nadirHb 80-90 deltaHb 10-20% | 2.738383 | 1.674514 | 0.337009 | 22.2509 | 1.647379 | 0.9596 |
| nadirHb 50-60 deltaHb 0-10% / nadirHb 50-60 deltaHb 20-30% | 0.568767 | 0.226134 | 0.145667 | 2.220793 | -1.41927 | 0.9900 |
| nadirHb 50-60 deltaHb 0-10% / nadirHb 60-70 deltaHb 20-30% | 1.008992 | 0.493491 | 0.188869 | 5.390322 | 0.018303 | 1.0000 |
| nadirHb 50-60 deltaHb 0-10% / nadirHb 70-80 deltaHb 20-30% | 1.789952 | 1.087868 | 0.223122 | 14.35951 | 0.95792 | 0.9999 |
| nadirHb 50-60 deltaHb 0-10% / nadirHb 80-90 deltaHb 20-30% | 3.175375 | 2.351856 | 0.251054 | 40.16271 | 1.560006 | 0.9751 |
| nadirHb 50-60 deltaHb 0-10% / nadirHb 50-60 deltaHb 30-40% | 0.428945 | 0.255814 | 0.055596 | 3.309497 | -1.41927 | 0.9900 |
| nadirHb 50-60 deltaHb 0-10% / nadirHb 60-70 deltaHb 30-40% | 0.87828 | 0.605311 | 0.082825 | 9.31326 | -0.18832 | 1.0000 |
| nadirHb 50-60 deltaHb 0-10% / nadirHb 70-80 deltaHb 30-40% | 1.798309 | 1.475743 | 0.108104 | 29.91479 | 0.715119 | 1.0000 |
| nadirHb 50-60 deltaHb 0-10% / nadirHb 80-90 deltaHb 30-40% | 3.682102 | 3.5907 | 0.130353 | 104.0092 | 1.336664 | 0.9946 |
| nadirHb 60-70 deltaHb 0-10% / nadirHb 70-80 deltaHb 0-10% | 1.331674 | 0.290078 | 0.631374 | 2.808725 | 1.314956 | 0.9955 |
| nadirHb 60-70 deltaHb 0-10% / nadirHb 80-90 deltaHb 0-10% | 1.773355 | 0.772579 | 0.398633 | 7.888934 | 1.314956 | 0.9955 |
| nadirHb 60-70 deltaHb 0-10% / nadirHb 50-60 deltaHb 10-20% | 0.56633 | 0.133079 | 0.253182 | 1.266794 | -2.41964 | 0.5325 |
| nadirHb 60-70 deltaHb 0-10% / nadirHb 60-70 deltaHb 10-20% | 0.870452 | 0.187975 | 0.415365 | 1.82415 | -0.64247 | 1.0000 |
| nadirHb 60-70 deltaHb 0-10% / nadirHb 70-80 deltaHb 10-20% | 1.337891 | 0.406561 | 0.472358 | 3.789394 | 0.95792 | 0.9999 |
| nadirHb 60-70 deltaHb 0-10% / nadirHb 80-90 deltaHb 10-20% | 2.056347 | 0.901858 | 0.457656 | 9.239616 | 1.643811 | 0.9604 |
| nadirHb 60-70 deltaHb 0-10% / nadirHb 50-60 deltaHb 20-30% | 0.427107 | 0.16096 | 0.117436 | 1.553356 | -2.25739 | 0.6556 |
| nadirHb 60-70 deltaHb 0-10% / nadirHb 60-70 deltaHb 20-30% | 0.757687 | 0.327246 | 0.172528 | 3.327523 | -0.64247 | 1.0000 |
| nadirHb 60-70 deltaHb 0-10% / nadirHb 70-80 deltaHb 20-30% | 1.344137 | 0.711355 | 0.21928 | 8.239245 | 0.558837 | 1.0000 |
| nadirHb 60-70 deltaHb 0-10% / nadirHb 80-90 deltaHb 20-30% | 2.384499 | 1.550205 | 0.257087 | 22.1164 | 1.336664 | 0.9946 |
| nadirHb 60-70 deltaHb 0-10% / nadirHb 50-60 deltaHb 30-40% | 0.322109 | 0.178725 | 0.048131 | 2.155671 | -2.04172 | 0.8017 |
| nadirHb 60-70 deltaHb 0-10% / nadirHb 60-70 deltaHb 30-40% | 0.659531 | 0.427278 | 0.071662 | 6.0699 | -0.64247 | 1.0000 |
| nadirHb 60-70 deltaHb 0-10% / nadirHb 70-80 deltaHb 30-40% | 1.350413 | 1.055279 | 0.092841 | 19.64237 | 0.384427 | 1.0000 |
| nadirHb 60-70 deltaHb 0-10% / nadirHb 80-90 deltaHb 30-40% | 2.765018 | 2.594878 | 0.111005 | 68.87362 | 1.083733 | 0.9995 |
| nadirHb 70-80 deltaHb 0-10% / nadirHb 80-90 deltaHb 0-10% | 1.331674 | 0.290078 | 0.631374 | 2.808725 | 1.314956 | 0.9955 |
| nadirHb 70-80 deltaHb 0-10% / nadirHb 50-60 deltaHb 10-20% | 0.425277 | 0.173175 | 0.105387 | 1.71616 | -2.09971 | 0.7657 |
| nadirHb 70-80 deltaHb 0-10% / nadirHb 60-70 deltaHb 10-20% | 0.653653 | 0.197694 | 0.231919 | 1.842291 | -1.40581 | 0.9909 |
| nadirHb 70-80 deltaHb 0-10% / nadirHb 70-80 deltaHb 10-20% | 1.004669 | 0.268496 | 0.402143 | 2.509949 | 0.017429 | 1.0000 |
| nadirHb 70-80 deltaHb 0-10% / nadirHb 80-90 deltaHb 10-20% | 1.544182 | 0.50195 | 0.507037 | 4.702808 | 1.336664 | 0.9946 |
| nadirHb 70-80 deltaHb 0-10% / nadirHb 50-60 deltaHb 20-30% | 0.320729 | 0.150733 | 0.064101 | 1.604768 | -2.41964 | 0.5325 |
| nadirHb 70-80 deltaHb 0-10% / nadirHb 60-70 deltaHb 20-30% | 0.568974 | 0.272137 | 0.110518 | 2.929218 | -1.17903 | 0.9986 |
| nadirHb 70-80 deltaHb 0-10% / nadirHb 70-80 deltaHb 20-30% | 1.009359 | 0.5395 | 0.161719 | 6.299844 | 0.017429 | 1.0000 |
| nadirHb 70-80 deltaHb 0-10% / nadirHb 80-90 deltaHb 20-30% | 1.790603 | 1.120575 | 0.209822 | 15.28083 | 0.93088 | 0.9999 |
| nadirHb 70-80 deltaHb 0-10% / nadirHb 50-60 deltaHb 30-40% | 0.241883 | 0.144111 | 0.031414 | 1.862459 | -2.38222 | 0.5611 |
| nadirHb 70-80 deltaHb 0-10% / nadirHb 60-70 deltaHb 30-40% | 0.495264 | 0.335659 | 0.048576 | 5.049515 | -1.03678 | 0.9997 |
| nadirHb 70-80 deltaHb 0-10% / nadirHb 70-80 deltaHb 30-40% | 1.014072 | 0.813028 | 0.065034 | 15.81229 | 0.017429 | 1.0000 |
| nadirHb 70-80 deltaHb 0-10% / nadirHb 80-90 deltaHb 30-40% | 2.076348 | 1.975664 | 0.079715 | 54.08305 | 0.767844 | 1.0000 |
| nadirHb 80-90 deltaHb 0-10% / nadirHb 50-60 deltaHb 10-20% | 0.319355 | 0.194601 | 0.039592 | 2.575975 | -1.87321 | 0.8883 |
| nadirHb 80-90 deltaHb 0-10% / nadirHb 60-70 deltaHb 10-20% | 0.490851 | 0.236022 | 0.094514 | 2.549189 | -1.47993 | 0.9849 |
| nadirHb 80-90 deltaHb 0-10% / nadirHb 70-80 deltaHb 10-20% | 0.754441 | 0.287677 | 0.204299 | 2.786017 | -0.73897 | 1.0000 |
| nadirHb 80-90 deltaHb 0-10% / nadirHb 80-90 deltaHb 10-20% | 1.15958 | 0.391313 | 0.364913 | 3.68479 | 0.438741 | 1.0000 |
| nadirHb 80-90 deltaHb 0-10% / nadirHb 50-60 deltaHb 20-30% | 0.240847 | 0.151297 | 0.027993 | 2.072182 | -2.26619 | 0.6491 |
| nadirHb 80-90 deltaHb 0-10% / nadirHb 60-70 deltaHb 20-30% | 0.427262 | 0.258446 | 0.053786 | 3.394037 | -1.40581 | 0.9909 |
| nadirHb 80-90 deltaHb 0-10% / nadirHb 70-80 deltaHb 20-30% | 0.757963 | 0.471031 | 0.090156 | 6.372359 | -0.44593 | 1.0000 |
| nadirHb 80-90 deltaHb 0-10% / nadirHb 80-90 deltaHb 20-30% | 1.344626 | 0.907518 | 0.133161 | 13.57768 | 0.438741 | 1.0000 |
| nadirHb 80-90 deltaHb 0-10% / nadirHb 50-60 deltaHb 30-40% | 0.181638 | 0.128047 | 0.016229 | 2.032911 | -2.41964 | 0.5325 |
| nadirHb 80-90 deltaHb 0-10% / nadirHb 60-70 deltaHb 30-40% | 0.371911 | 0.286599 | 0.026536 | 5.212436 | -1.28353 | 0.9965 |
| nadirHb 80-90 deltaHb 0-10% / nadirHb 70-80 deltaHb 30-40% | 0.761502 | 0.66814 | 0.037686 | 15.38727 | -0.31054 | 1.0000 |
| nadirHb 80-90 deltaHb 0-10% / nadirHb 80-90 deltaHb 30-40% | 1.559202 | 1.57851 | 0.048592 | 50.03088 | 0.438741 | 1.0000 |
| nadirHb 50-60 deltaHb 10-20% / nadirHb 60-70 deltaHb 10-20% | 1.537007 | 0.253239 | 0.874028 | 2.702876 | 2.608843 | 0.3921 |
| nadirHb 50-60 deltaHb 10-20% / nadirHb 70-80 deltaHb 10-20% | 2.362389 | 0.778461 | 0.763925 | 7.305536 | 2.608843 | 0.3921 |
| nadirHb 50-60 deltaHb 10-20% / nadirHb 80-90 deltaHb 10-20% | 3.631008 | 1.79475 | 0.667692 | 19.74595 | 2.608843 | 0.3921 |
| nadirHb 50-60 deltaHb 10-20% / nadirHb 50-60 deltaHb 20-30% | 0.754166 | 0.149923 | 0.381663 | 1.490232 | -1.41927 | 0.9900 |
| nadirHb 50-60 deltaHb 10-20% / nadirHb 60-70 deltaHb 20-30% | 1.337891 | 0.406561 | 0.472358 | 3.789394 | 0.95792 | 0.9999 |
| nadirHb 50-60 deltaHb 10-20% / nadirHb 70-80 deltaHb 20-30% | 2.373419 | 1.045914 | 0.524426 | 10.74149 | 1.961365 | 0.8466 |
| nadirHb 50-60 deltaHb 10-20% / nadirHb 80-90 deltaHb 20-30% | 4.210444 | 2.473358 | 0.562702 | 31.50484 | 2.4472 | 0.5114 |
| nadirHb 50-60 deltaHb 10-20% / nadirHb 50-60 deltaHb 30-40% | 0.568767 | 0.226134 | 0.145667 | 2.220793 | -1.41927 | 0.9900 |
| nadirHb 50-60 deltaHb 10-20% / nadirHb 60-70 deltaHb 30-40% | 1.16457 | 0.583229 | 0.209413 | 6.476309 | 0.304211 | 1.0000 |
| nadirHb 50-60 deltaHb 10-20% / nadirHb 70-80 deltaHb 30-40% | 2.384499 | 1.550205 | 0.257087 | 22.1164 | 1.336664 | 0.9946 |
| nadirHb 50-60 deltaHb 10-20% / nadirHb 80-90 deltaHb 30-40% | 4.882348 | 4.007097 | 0.293395 | 81.24638 | 1.931967 | 0.8614 |
| nadirHb 60-70 deltaHb 10-20% / nadirHb 70-80 deltaHb 10-20% | 1.537007 | 0.253239 | 0.874028 | 2.702876 | 2.608843 | 0.3921 |
| nadirHb 60-70 deltaHb 10-20% / nadirHb 80-90 deltaHb 10-20% | 2.362389 | 0.778461 | 0.763925 | 7.305536 | 2.608843 | 0.3921 |
| nadirHb 60-70 deltaHb 10-20% / nadirHb 50-60 deltaHb 20-30% | 0.490672 | 0.093915 | 0.254686 | 0.945319 | -3.71984 | 0.0184 |
| nadirHb 60-70 deltaHb 10-20% / nadirHb 60-70 deltaHb 20-30% | 0.870452 | 0.187975 | 0.415365 | 1.82415 | -0.64247 | 1.0000 |
| nadirHb 60-70 deltaHb 10-20% / nadirHb 70-80 deltaHb 20-30% | 1.544182 | 0.50195 | 0.507037 | 4.702808 | 1.336664 | 0.9946 |
| nadirHb 60-70 deltaHb 10-20% / nadirHb 80-90 deltaHb 20-30% | 2.73938 | 1.266411 | 0.562074 | 13.35091 | 2.179829 | 0.7117 |
| nadirHb 60-70 deltaHb 10-20% / nadirHb 50-60 deltaHb 30-40% | 0.370048 | 0.130915 | 0.11012 | 1.243512 | -2.81001 | 0.2630 |
| nadirHb 60-70 deltaHb 10-20% / nadirHb 60-70 deltaHb 30-40% | 0.757687 | 0.327246 | 0.172528 | 3.327523 | -0.64247 | 1.0000 |
| nadirHb 60-70 deltaHb 10-20% / nadirHb 70-80 deltaHb 30-40% | 1.551392 | 0.887179 | 0.218698 | 11.0052 | 0.767937 | 1.0000 |
| nadirHb 60-70 deltaHb 10-20% / nadirHb 80-90 deltaHb 30-40% | 3.17653 | 2.348515 | 0.252285 | 39.99589 | 1.563286 | 0.9746 |
| nadirHb 70-80 deltaHb 10-20% / nadirHb 80-90 deltaHb 10-20% | 1.537007 | 0.253239 | 0.874028 | 2.702876 | 2.608843 | 0.3921 |
| nadirHb 70-80 deltaHb 10-20% / nadirHb 50-60 deltaHb 20-30% | 0.319239 | 0.094724 | 0.115511 | 0.882281 | -3.84814 | 0.0115 |
| nadirHb 70-80 deltaHb 10-20% / nadirHb 60-70 deltaHb 20-30% | 0.56633 | 0.133079 | 0.253182 | 1.266794 | -2.41964 | 0.5325 |
| nadirHb 70-80 deltaHb 10-20% / nadirHb 70-80 deltaHb 20-30% | 1.004669 | 0.268496 | 0.402143 | 2.509949 | 0.017429 | 1.0000 |
| nadirHb 70-80 deltaHb 10-20% / nadirHb 80-90 deltaHb 20-30% | 1.782282 | 0.658851 | 0.502279 | 6.32423 | 1.563286 | 0.9746 |
| nadirHb 70-80 deltaHb 10-20% / nadirHb 50-60 deltaHb 30-40% | 0.240759 | 0.092163 | 0.064865 | 0.893627 | -3.71984 | 0.0184 |
| nadirHb 70-80 deltaHb 10-20% / nadirHb 60-70 deltaHb 30-40% | 0.492963 | 0.207138 | 0.116845 | 2.079786 | -1.68334 | 0.9515 |
| nadirHb 70-80 deltaHb 10-20% / nadirHb 70-80 deltaHb 30-40% | 1.009359 | 0.5395 | 0.161719 | 6.299844 | 0.017429 | 1.0000 |
| nadirHb 70-80 deltaHb 10-20% / nadirHb 80-90 deltaHb 30-40% | 2.066699 | 1.422755 | 0.195421 | 21.85663 | 1.054522 | 0.9996 |
| nadirHb 80-90 deltaHb 10-20% / nadirHb 50-60 deltaHb 20-30% | 0.207702 | 0.091422 | 0.045975 | 0.938344 | -3.57062 | 0.0311 |
| nadirHb 80-90 deltaHb 10-20% / nadirHb 60-70 deltaHb 20-30% | 0.368463 | 0.126621 | 0.113521 | 1.195944 | -2.90535 | 0.2121 |
| nadirHb 80-90 deltaHb 10-20% / nadirHb 70-80 deltaHb 20-30% | 0.653653 | 0.197694 | 0.231919 | 1.842291 | -1.40581 | 0.9909 |
| nadirHb 80-90 deltaHb 10-20% / nadirHb 80-90 deltaHb 20-30% | 1.15958 | 0.391313 | 0.364913 | 3.68479 | 0.438741 | 1.0000 |
| nadirHb 80-90 deltaHb 10-20% / nadirHb 50-60 deltaHb 30-40% | 0.156642 | 0.073839 | 0.031155 | 0.787571 | -3.93264 | 0.0083 |
| nadirHb 80-90 deltaHb 10-20% / nadirHb 60-70 deltaHb 30-40% | 0.320729 | 0.150733 | 0.064101 | 1.604768 | -2.41964 | 0.5325 |
| nadirHb 80-90 deltaHb 10-20% / nadirHb 70-80 deltaHb 30-40% | 0.656705 | 0.358878 | 0.100984 | 4.270584 | -0.76951 | 1.0000 |
| nadirHb 80-90 deltaHb 10-20% / nadirHb 80-90 deltaHb 30-40% | 1.344626 | 0.907518 | 0.133161 | 13.57768 | 0.438741 | 1.0000 |
| nadirHb 50-60 deltaHb 20-30% / nadirHb 60-70 deltaHb 20-30% | 1.774 | 0.277754 | 1.03751 | 3.033296 | 3.661233 | 0.0227 |
| nadirHb 50-60 deltaHb 20-30% / nadirHb 70-80 deltaHb 20-30% | 3.147076 | 0.985471 | 1.076427 | 9.200887 | 3.661233 | 0.0227 |
| nadirHb 50-60 deltaHb 20-30% / nadirHb 80-90 deltaHb 20-30% | 5.582913 | 2.622339 | 1.116804 | 27.90902 | 3.661233 | 0.0227 |
| nadirHb 50-60 deltaHb 20-30% / nadirHb 50-60 deltaHb 30-40% | 0.754166 | 0.149923 | 0.381663 | 1.490232 | -1.41927 | 0.9900 |
| nadirHb 50-60 deltaHb 20-30% / nadirHb 60-70 deltaHb 30-40% | 1.544182 | 0.50195 | 0.507037 | 4.702808 | 1.336664 | 0.9946 |
| nadirHb 50-60 deltaHb 20-30% / nadirHb 70-80 deltaHb 30-40% | 3.161769 | 1.583769 | 0.568352 | 17.58908 | 2.29807 | 0.6252 |
| nadirHb 50-60 deltaHb 20-30% / nadirHb 80-90 deltaHb 30-40% | 6.473835 | 4.463044 | 0.610099 | 68.6947 | 2.709278 | 0.3242 |
| nadirHb 60-70 deltaHb 20-30% / nadirHb 70-80 deltaHb 20-30% | 1.774 | 0.277754 | 1.03751 | 3.033296 | 3.661233 | 0.0227 |
| nadirHb 60-70 deltaHb 20-30% / nadirHb 80-90 deltaHb 20-30% | 3.147076 | 0.985471 | 1.076427 | 9.200887 | 3.661233 | 0.0227 |
| nadirHb 60-70 deltaHb 20-30% / nadirHb 50-60 deltaHb 30-40% | 0.425122 | 0.080349 | 0.222483 | 0.812326 | -4.52579 | 0.0007 |
| nadirHb 60-70 deltaHb 20-30% / nadirHb 60-70 deltaHb 30-40% | 0.870452 | 0.187975 | 0.415365 | 1.82415 | -0.64247 | 1.0000 |
| nadirHb 60-70 deltaHb 20-30% / nadirHb 70-80 deltaHb 30-40% | 1.782282 | 0.658851 | 0.502279 | 6.32423 | 1.563286 | 0.9746 |
| nadirHb 60-70 deltaHb 20-30% / nadirHb 80-90 deltaHb 30-40% | 3.649287 | 2.017952 | 0.548827 | 24.26502 | 2.341045 | 0.5926 |
| nadirHb 70-80 deltaHb 20-30% / nadirHb 80-90 deltaHb 20-30% | 1.774 | 0.277754 | 1.03751 | 3.033296 | 3.661233 | 0.0227 |
| nadirHb 70-80 deltaHb 20-30% / nadirHb 50-60 deltaHb 30-40% | 0.23964 | 0.068183 | 0.09041 | 0.635186 | -5.02114 | 0.0001 |
| nadirHb 70-80 deltaHb 20-30% / nadirHb 60-70 deltaHb 30-40% | 0.490672 | 0.093915 | 0.254686 | 0.945319 | -3.71984 | 0.0184 |
| nadirHb 70-80 deltaHb 20-30% / nadirHb 70-80 deltaHb 30-40% | 1.004669 | 0.268496 | 0.402143 | 2.509949 | 0.017429 | 1.0000 |
| nadirHb 70-80 deltaHb 20-30% / nadirHb 80-90 deltaHb 30-40% | 2.057095 | 0.885543 | 0.47069 | 8.990285 | 1.675551 | 0.9533 |
| nadirHb 80-90 deltaHb 20-30% / nadirHb 50-60 deltaHb 30-40% | 0.135085 | 0.056544 | 0.032195 | 0.566785 | -4.78247 | 0.0002 |
| nadirHb 80-90 deltaHb 20-30% / nadirHb 60-70 deltaHb 30-40% | 0.276591 | 0.076081 | 0.107786 | 0.70976 | -4.67239 | 0.0003 |
| nadirHb 80-90 deltaHb 20-30% / nadirHb 70-80 deltaHb 30-40% | 0.56633 | 0.133079 | 0.253182 | 1.266794 | -2.41964 | 0.5325 |
| nadirHb 80-90 deltaHb 20-30% / nadirHb 80-90 deltaHb 30-40% | 1.15958 | 0.391313 | 0.364913 | 3.68479 | 0.438741 | 1.0000 |
| nadirHb 50-60 deltaHb 30-40% / nadirHb 60-70 deltaHb 30-40% | 2.047536 | 0.407211 | 1.0359 | 4.047112 | 3.603388 | 0.0278 |
| nadirHb 50-60 deltaHb 30-40% / nadirHb 70-80 deltaHb 30-40% | 4.192403 | 1.667559 | 1.073089 | 16.37912 | 3.603388 | 0.0278 |
| nadirHb 50-60 deltaHb 30-40% / nadirHb 80-90 deltaHb 30-40% | 8.584096 | 5.12158 | 1.111612 | 66.28812 | 3.603388 | 0.0278 |
| nadirHb 60-70 deltaHb 30-40% / nadirHb 70-80 deltaHb 30-40% | 2.047536 | 0.407211 | 1.0359 | 4.047112 | 3.603388 | 0.0278 |
| nadirHb 60-70 deltaHb 30-40% / nadirHb 80-90 deltaHb 30-40% | 4.192403 | 1.667559 | 1.073089 | 16.37912 | 3.603388 | 0.0278 |
| nadirHb 70-80 deltaHb 30-40% / nadirHb 80-90 deltaHb 30-40% | 2.047536 | 0.407211 | 1.0359 | 4.047112 | 3.603388 | 0.0278 |

# **Table S6.** Prediction of the bleeding-related mortality in the red blood cell transfused population – subgroup comparisons.

| **Comparison** | **Odds ratio** | **Standard error** | **Lower Confidece Interval** | **Upper Confidence Interval** | **Z ratio** | **P-value** |
| --- | --- | --- | --- | --- | --- | --- |
| nadirHb 50-60 deltaHb 0-10% / nadirHb 60-70 deltaHb 0-10% | 1.237176 | 0.529082 | 0.285839 | 5.354775 | 0.497672 | 1.0000 |
| nadirHb 50-60 deltaHb 0-10% / nadirHb 70-80 deltaHb 0-10% | 1.530604 | 1.309135 | 0.081704 | 28.67362 | 0.497672 | 1.0000 |
| nadirHb 50-60 deltaHb 0-10% / nadirHb 80-90 deltaHb 0-10% | 1.893626 | 2.429446 | 0.023354 | 153.5408 | 0.497672 | 1.0000 |
| nadirHb 50-60 deltaHb 0-10% / nadirHb 50-60 deltaHb 10-20% | 0.747227 | 0.166026 | 0.349023 | 1.599746 | -1.31143 | 0.9956 |
| nadirHb 50-60 deltaHb 0-10% / nadirHb 60-70 deltaHb 10-20% | 1.132551 | 0.500371 | 0.249279 | 5.145532 | 0.281734 | 1.0000 |
| nadirHb 50-60 deltaHb 0-10% / nadirHb 70-80 deltaHb 10-20% | 1.716576 | 1.256358 | 0.139851 | 21.06986 | 0.738261 | 1.0000 |
| nadirHb 50-60 deltaHb 0-10% / nadirHb 80-90 deltaHb 10-20% | 2.601765 | 2.6923 | 0.07509 | 90.1477 | 0.924036 | 0.9999 |
| nadirHb 50-60 deltaHb 0-10% / nadirHb 50-60 deltaHb 20-30% | 0.558348 | 0.248118 | 0.121817 | 2.559186 | -1.31143 | 0.9956 |
| nadirHb 50-60 deltaHb 0-10% / nadirHb 60-70 deltaHb 20-30% | 1.036774 | 0.618617 | 0.134241 | 8.007243 | 0.060526 | 1.0000 |
| nadirHb 50-60 deltaHb 0-10% / nadirHb 70-80 deltaHb 20-30% | 1.925144 | 1.533047 | 0.125776 | 29.46655 | 0.822525 | 1.0000 |
| nadirHb 50-60 deltaHb 0-10% / nadirHb 80-90 deltaHb 20-30% | 3.57472 | 3.631164 | 0.110113 | 116.0504 | 1.254085 | 0.9973 |
| nadirHb 50-60 deltaHb 0-10% / nadirHb 50-60 deltaHb 30-40% | 0.417213 | 0.278101 | 0.042517 | 4.094047 | -1.31143 | 0.9956 |
| nadirHb 50-60 deltaHb 0-10% / nadirHb 60-70 deltaHb 30-40% | 0.949097 | 0.774177 | 0.058026 | 15.52376 | -0.06405 | 1.0000 |
| nadirHb 50-60 deltaHb 0-10% / nadirHb 70-80 deltaHb 30-40% | 2.159053 | 2.187946 | 0.067056 | 69.51618 | 0.759506 | 1.0000 |
| nadirHb 50-60 deltaHb 0-10% / nadirHb 80-90 deltaHb 30-40% | 4.911521 | 6.073337 | 0.071014 | 339.6964 | 1.287117 | 0.9964 |
| nadirHb 60-70 deltaHb 0-10% / nadirHb 70-80 deltaHb 0-10% | 1.237176 | 0.529082 | 0.285839 | 5.354775 | 0.497672 | 1.0000 |
| nadirHb 60-70 deltaHb 0-10% / nadirHb 80-90 deltaHb 0-10% | 1.530604 | 1.309135 | 0.081704 | 28.67362 | 0.497672 | 1.0000 |
| nadirHb 60-70 deltaHb 0-10% / nadirHb 50-60 deltaHb 10-20% | 0.603978 | 0.259524 | 0.138573 | 2.632474 | -1.17344 | 0.9987 |
| nadirHb 60-70 deltaHb 0-10% / nadirHb 60-70 deltaHb 10-20% | 0.915433 | 0.24946 | 0.359885 | 2.328568 | -0.32425 | 1.0000 |
| nadirHb 60-70 deltaHb 0-10% / nadirHb 70-80 deltaHb 10-20% | 1.387495 | 0.552451 | 0.354649 | 5.42831 | 0.822525 | 1.0000 |
| nadirHb 60-70 deltaHb 0-10% / nadirHb 80-90 deltaHb 10-20% | 2.102988 | 1.390937 | 0.218131 | 20.27477 | 1.123901 | 0.9992 |
| nadirHb 60-70 deltaHb 0-10% / nadirHb 50-60 deltaHb 20-30% | 0.451309 | 0.240983 | 0.07244 | 2.811686 | -1.48999 | 0.9838 |
| nadirHb 60-70 deltaHb 0-10% / nadirHb 60-70 deltaHb 20-30% | 0.838017 | 0.456727 | 0.129517 | 5.422231 | -0.32425 | 1.0000 |
| nadirHb 60-70 deltaHb 0-10% / nadirHb 70-80 deltaHb 20-30% | 1.556079 | 1.018634 | 0.165204 | 14.65689 | 0.675464 | 1.0000 |
| nadirHb 60-70 deltaHb 0-10% / nadirHb 80-90 deltaHb 20-30% | 2.88942 | 2.38194 | 0.171485 | 48.68487 | 1.287117 | 0.9964 |
| nadirHb 60-70 deltaHb 0-10% / nadirHb 50-60 deltaHb 30-40% | 0.33723 | 0.234694 | 0.031076 | 3.659576 | -1.56189 | 0.9749 |
| nadirHb 60-70 deltaHb 0-10% / nadirHb 60-70 deltaHb 30-40% | 0.767148 | 0.627154 | 0.046611 | 12.62604 | -0.32425 | 1.0000 |
| nadirHb 60-70 deltaHb 0-10% / nadirHb 70-80 deltaHb 30-40% | 1.745146 | 1.738895 | 0.057445 | 53.01671 | 0.55884 | 1.0000 |
| nadirHb 60-70 deltaHb 0-10% / nadirHb 80-90 deltaHb 30-40% | 3.969946 | 4.79345 | 0.06342 | 248.5075 | 1.141886 | 0.9991 |
| nadirHb 70-80 deltaHb 0-10% / nadirHb 80-90 deltaHb 0-10% | 1.237176 | 0.529082 | 0.285839 | 5.354775 | 0.497672 | 1.0000 |
| nadirHb 70-80 deltaHb 0-10% / nadirHb 50-60 deltaHb 10-20% | 0.488191 | 0.404249 | 0.02861 | 8.330343 | -0.86594 | 1.0000 |
| nadirHb 70-80 deltaHb 0-10% / nadirHb 60-70 deltaHb 10-20% | 0.739937 | 0.417981 | 0.10683 | 5.125024 | -0.53319 | 1.0000 |
| nadirHb 70-80 deltaHb 0-10% / nadirHb 70-80 deltaHb 10-20% | 1.121502 | 0.430239 | 0.301299 | 4.174487 | 0.298908 | 1.0000 |
| nadirHb 70-80 deltaHb 0-10% / nadirHb 80-90 deltaHb 10-20% | 1.699829 | 0.700641 | 0.414108 | 6.977454 | 1.287117 | 0.9964 |
| nadirHb 70-80 deltaHb 0-10% / nadirHb 50-60 deltaHb 20-30% | 0.36479 | 0.313494 | 0.019202 | 6.929922 | -1.17344 | 0.9987 |
| nadirHb 70-80 deltaHb 0-10% / nadirHb 60-70 deltaHb 20-30% | 0.677363 | 0.526352 | 0.047274 | 9.705526 | -0.50131 | 1.0000 |
| nadirHb 70-80 deltaHb 0-10% / nadirHb 70-80 deltaHb 20-30% | 1.257767 | 0.965027 | 0.090781 | 17.42634 | 0.298908 | 1.0000 |
| nadirHb 70-80 deltaHb 0-10% / nadirHb 80-90 deltaHb 20-30% | 2.335497 | 1.944369 | 0.134788 | 40.46769 | 1.018853 | 0.9998 |
| nadirHb 70-80 deltaHb 0-10% / nadirHb 50-60 deltaHb 30-40% | 0.272581 | 0.257172 | 0.010757 | 6.90713 | -1.3777 | 0.9926 |
| nadirHb 70-80 deltaHb 0-10% / nadirHb 60-70 deltaHb 30-40% | 0.62008 | 0.631468 | 0.018933 | 20.30884 | -0.46929 | 1.0000 |
| nadirHb 70-80 deltaHb 0-10% / nadirHb 70-80 deltaHb 30-40% | 1.410589 | 1.62342 | 0.027352 | 72.74602 | 0.298908 | 1.0000 |
| nadirHb 70-80 deltaHb 0-10% / nadirHb 80-90 deltaHb 30-40% | 3.208878 | 4.247998 | 0.034405 | 299.2879 | 0.880721 | 1.0000 |
| nadirHb 80-90 deltaHb 0-10% / nadirHb 50-60 deltaHb 10-20% | 0.394601 | 0.491669 | 0.005524 | 28.18909 | -0.7463 | 1.0000 |
| nadirHb 80-90 deltaHb 0-10% / nadirHb 60-70 deltaHb 10-20% | 0.598086 | 0.576683 | 0.021984 | 16.27154 | -0.5331 | 1.0000 |
| nadirHb 80-90 deltaHb 0-10% / nadirHb 70-80 deltaHb 10-20% | 0.906502 | 0.642005 | 0.080093 | 10.25984 | -0.1386 | 1.0000 |
| nadirHb 80-90 deltaHb 0-10% / nadirHb 80-90 deltaHb 10-20% | 1.373959 | 0.711415 | 0.233108 | 8.098257 | 0.613569 | 1.0000 |
| nadirHb 80-90 deltaHb 0-10% / nadirHb 50-60 deltaHb 20-30% | 0.294857 | 0.368009 | 0.004098 | 21.2159 | -0.97851 | 0.9999 |
| nadirHb 80-90 deltaHb 0-10% / nadirHb 60-70 deltaHb 20-30% | 0.547507 | 0.618559 | 0.011413 | 26.26587 | -0.53319 | 1.0000 |
| nadirHb 80-90 deltaHb 0-10% / nadirHb 70-80 deltaHb 20-30% | 1.016644 | 1.073323 | 0.027309 | 37.8471 | 0.015635 | 1.0000 |
| nadirHb 80-90 deltaHb 0-10% / nadirHb 80-90 deltaHb 20-30% | 1.887765 | 1.954912 | 0.054339 | 65.58177 | 0.613569 | 1.0000 |
| nadirHb 80-90 deltaHb 0-10% / nadirHb 50-60 deltaHb 30-40% | 0.220325 | 0.284015 | 0.002661 | 18.24284 | -1.17344 | 0.9987 |
| nadirHb 80-90 deltaHb 0-10% / nadirHb 60-70 deltaHb 30-40% | 0.501206 | 0.667109 | 0.005243 | 47.9113 | -0.51896 | 1.0000 |
| nadirHb 80-90 deltaHb 0-10% / nadirHb 70-80 deltaHb 30-40% | 1.140169 | 1.621279 | 0.008734 | 148.8417 | 0.09225 | 1.0000 |
| nadirHb 80-90 deltaHb 0-10% / nadirHb 80-90 deltaHb 30-40% | 2.593712 | 4.028954 | 0.012667 | 531.0981 | 0.613569 | 1.0000 |
| nadirHb 50-60 deltaHb 10-20% / nadirHb 60-70 deltaHb 10-20% | 1.515672 | 0.472846 | 0.520499 | 4.413577 | 1.333003 | 0.9947 |
| nadirHb 50-60 deltaHb 10-20% / nadirHb 70-80 deltaHb 10-20% | 2.297261 | 1.433359 | 0.270919 | 19.47966 | 1.333003 | 0.9947 |
| nadirHb 50-60 deltaHb 10-20% / nadirHb 80-90 deltaHb 10-20% | 3.481894 | 3.258753 | 0.141013 | 85.975 | 1.333003 | 0.9947 |
| nadirHb 50-60 deltaHb 10-20% / nadirHb 50-60 deltaHb 20-30% | 0.747227 | 0.166026 | 0.349023 | 1.599746 | -1.31143 | 0.9956 |
| nadirHb 50-60 deltaHb 10-20% / nadirHb 60-70 deltaHb 20-30% | 1.387495 | 0.552451 | 0.354649 | 5.42831 | 0.822525 | 1.0000 |
| nadirHb 50-60 deltaHb 10-20% / nadirHb 70-80 deltaHb 20-30% | 2.576383 | 1.603314 | 0.305538 | 21.72478 | 1.520759 | 0.9804 |
| nadirHb 50-60 deltaHb 10-20% / nadirHb 80-90 deltaHb 20-30% | 4.783981 | 4.103028 | 0.253319 | 90.34641 | 1.825051 | 0.9078 |
| nadirHb 50-60 deltaHb 10-20% / nadirHb 50-60 deltaHb 30-40% | 0.558348 | 0.248118 | 0.121817 | 2.559186 | -1.31143 | 0.9956 |
| nadirHb 50-60 deltaHb 10-20% / nadirHb 60-70 deltaHb 30-40% | 1.270159 | 0.77079 | 0.158828 | 10.15756 | 0.394074 | 1.0000 |
| nadirHb 50-60 deltaHb 10-20% / nadirHb 70-80 deltaHb 30-40% | 2.88942 | 2.38194 | 0.171485 | 48.68487 | 1.287117 | 0.9964 |
| nadirHb 50-60 deltaHb 10-20% / nadirHb 80-90 deltaHb 30-40% | 6.572996 | 6.991604 | 0.171828 | 251.4395 | 1.770231 | 0.9270 |
| nadirHb 60-70 deltaHb 10-20% / nadirHb 70-80 deltaHb 10-20% | 1.515672 | 0.472846 | 0.520499 | 4.413577 | 1.333003 | 0.9947 |
| nadirHb 60-70 deltaHb 10-20% / nadirHb 80-90 deltaHb 10-20% | 2.297261 | 1.433359 | 0.270919 | 19.47966 | 1.333003 | 0.9947 |
| nadirHb 60-70 deltaHb 10-20% / nadirHb 50-60 deltaHb 20-30% | 0.493001 | 0.154486 | 0.168499 | 1.442437 | -2.25698 | 0.6559 |
| nadirHb 60-70 deltaHb 10-20% / nadirHb 60-70 deltaHb 20-30% | 0.915433 | 0.24946 | 0.359885 | 2.328568 | -0.32425 | 1.0000 |
| nadirHb 60-70 deltaHb 10-20% / nadirHb 70-80 deltaHb 20-30% | 1.699829 | 0.700641 | 0.414108 | 6.977454 | 1.287117 | 0.9964 |
| nadirHb 60-70 deltaHb 10-20% / nadirHb 80-90 deltaHb 20-30% | 3.156343 | 1.958718 | 0.376563 | 26.4564 | 1.852204 | 0.8971 |
| nadirHb 60-70 deltaHb 10-20% / nadirHb 50-60 deltaHb 30-40% | 0.368383 | 0.163836 | 0.080271 | 1.690595 | -2.24541 | 0.6645 |
| nadirHb 60-70 deltaHb 10-20% / nadirHb 60-70 deltaHb 30-40% | 0.838017 | 0.456727 | 0.129517 | 5.422231 | -0.32425 | 1.0000 |
| nadirHb 60-70 deltaHb 10-20% / nadirHb 70-80 deltaHb 30-40% | 1.906362 | 1.396732 | 0.154903 | 23.46124 | 0.880612 | 1.0000 |
| nadirHb 60-70 deltaHb 10-20% / nadirHb 80-90 deltaHb 30-40% | 4.336688 | 4.153047 | 0.163025 | 115.3618 | 1.531984 | 0.9790 |
| nadirHb 70-80 deltaHb 10-20% / nadirHb 80-90 deltaHb 10-20% | 1.515672 | 0.472846 | 0.520499 | 4.413577 | 1.333003 | 0.9947 |
| nadirHb 70-80 deltaHb 10-20% / nadirHb 50-60 deltaHb 20-30% | 0.325269 | 0.190128 | 0.043906 | 2.409713 | -1.92139 | 0.8665 |
| nadirHb 70-80 deltaHb 10-20% / nadirHb 60-70 deltaHb 20-30% | 0.603978 | 0.259524 | 0.138573 | 2.632474 | -1.17344 | 0.9987 |
| nadirHb 70-80 deltaHb 10-20% / nadirHb 70-80 deltaHb 20-30% | 1.121502 | 0.430239 | 0.301299 | 4.174487 | 0.298908 | 1.0000 |
| nadirHb 70-80 deltaHb 10-20% / nadirHb 80-90 deltaHb 20-30% | 2.082472 | 0.997144 | 0.403764 | 10.74066 | 1.531984 | 0.9790 |
| nadirHb 70-80 deltaHb 10-20% / nadirHb 50-60 deltaHb 30-40% | 0.24305 | 0.152323 | 0.028392 | 2.080624 | -2.25698 | 0.6559 |
| nadirHb 70-80 deltaHb 10-20% / nadirHb 60-70 deltaHb 30-40% | 0.552901 | 0.358518 | 0.059957 | 5.09863 | -0.91386 | 0.9999 |
| nadirHb 70-80 deltaHb 10-20% / nadirHb 70-80 deltaHb 30-40% | 1.257767 | 0.965027 | 0.090781 | 17.42634 | 0.298908 | 1.0000 |
| nadirHb 70-80 deltaHb 10-20% / nadirHb 80-90 deltaHb 30-40% | 2.861232 | 2.710519 | 0.111434 | 73.46658 | 1.109705 | 0.9993 |
| nadirHb 80-90 deltaHb 10-20% / nadirHb 50-60 deltaHb 20-30% | 0.214604 | 0.189509 | 0.010416 | 4.421367 | -1.74275 | 0.9355 |
| nadirHb 80-90 deltaHb 10-20% / nadirHb 60-70 deltaHb 20-30% | 0.398489 | 0.278846 | 0.036244 | 4.381228 | -1.31485 | 0.9955 |
| nadirHb 80-90 deltaHb 10-20% / nadirHb 70-80 deltaHb 20-30% | 0.739937 | 0.417981 | 0.10683 | 5.125024 | -0.53319 | 1.0000 |
| nadirHb 80-90 deltaHb 10-20% / nadirHb 80-90 deltaHb 20-30% | 1.373959 | 0.711415 | 0.233108 | 8.098257 | 0.613569 | 1.0000 |
| nadirHb 80-90 deltaHb 10-20% / nadirHb 50-60 deltaHb 30-40% | 0.160358 | 0.141842 | 0.007744 | 3.320469 | -2.06928 | 0.7850 |
| nadirHb 80-90 deltaHb 10-20% / nadirHb 60-70 deltaHb 30-40% | 0.36479 | 0.313494 | 0.019202 | 6.929922 | -1.17344 | 0.9987 |
| nadirHb 80-90 deltaHb 10-20% / nadirHb 70-80 deltaHb 30-40% | 0.829841 | 0.758389 | 0.036241 | 19.00168 | -0.20409 | 1.0000 |
| nadirHb 80-90 deltaHb 10-20% / nadirHb 80-90 deltaHb 30-40% | 1.887765 | 1.954912 | 0.054339 | 65.58177 | 0.613569 | 1.0000 |
| nadirHb 50-60 deltaHb 20-30% / nadirHb 60-70 deltaHb 20-30% | 1.856859 | 0.454022 | 0.803469 | 4.291299 | 2.531124 | 0.4483 |
| nadirHb 50-60 deltaHb 20-30% / nadirHb 70-80 deltaHb 20-30% | 3.447925 | 1.686108 | 0.645562 | 18.41525 | 2.531124 | 0.4483 |
| nadirHb 50-60 deltaHb 20-30% / nadirHb 80-90 deltaHb 20-30% | 6.402311 | 4.696297 | 0.518689 | 79.02534 | 2.531124 | 0.4483 |
| nadirHb 50-60 deltaHb 20-30% / nadirHb 50-60 deltaHb 30-40% | 0.747227 | 0.166026 | 0.349023 | 1.599746 | -1.31143 | 0.9956 |
| nadirHb 50-60 deltaHb 20-30% / nadirHb 60-70 deltaHb 30-40% | 1.699829 | 0.700641 | 0.414108 | 6.977454 | 1.287117 | 0.9964 |
| nadirHb 50-60 deltaHb 20-30% / nadirHb 70-80 deltaHb 30-40% | 3.866856 | 2.538472 | 0.407935 | 36.65435 | 2.060176 | 0.7906 |
| nadirHb 50-60 deltaHb 20-30% / nadirHb 80-90 deltaHb 30-40% | 8.796516 | 8.026254 | 0.386089 | 200.4165 | 2.383024 | 0.5605 |
| nadirHb 60-70 deltaHb 20-30% / nadirHb 70-80 deltaHb 20-30% | 1.856859 | 0.454022 | 0.803469 | 4.291299 | 2.531124 | 0.4483 |
| nadirHb 60-70 deltaHb 20-30% / nadirHb 80-90 deltaHb 20-30% | 3.447925 | 1.686108 | 0.645562 | 18.41525 | 2.531124 | 0.4483 |
| nadirHb 60-70 deltaHb 20-30% / nadirHb 50-60 deltaHb 30-40% | 0.402415 | 0.098384 | 0.174142 | 0.929916 | -3.72324 | 0.0182 |
| nadirHb 60-70 deltaHb 20-30% / nadirHb 60-70 deltaHb 30-40% | 0.915433 | 0.24946 | 0.359885 | 2.328568 | -0.32425 | 1.0000 |
| nadirHb 60-70 deltaHb 20-30% / nadirHb 70-80 deltaHb 30-40% | 2.082472 | 0.997144 | 0.403764 | 10.74066 | 1.531984 | 0.9790 |
| nadirHb 60-70 deltaHb 20-30% / nadirHb 80-90 deltaHb 30-40% | 4.73731 | 3.431925 | 0.395932 | 56.6817 | 2.147116 | 0.7343 |
| nadirHb 70-80 deltaHb 20-30% / nadirHb 80-90 deltaHb 20-30% | 1.856859 | 0.454022 | 0.803469 | 4.291299 | 2.531124 | 0.4483 |
| nadirHb 70-80 deltaHb 20-30% / nadirHb 50-60 deltaHb 30-40% | 0.216718 | 0.094402 | 0.048726 | 0.963893 | -3.51047 | 0.0380 |
| nadirHb 70-80 deltaHb 20-30% / nadirHb 60-70 deltaHb 30-40% | 0.493001 | 0.154486 | 0.168499 | 1.442437 | -2.25698 | 0.6559 |
| nadirHb 70-80 deltaHb 20-30% / nadirHb 70-80 deltaHb 30-40% | 1.121502 | 0.430239 | 0.301299 | 4.174487 | 0.298908 | 1.0000 |
| nadirHb 70-80 deltaHb 20-30% / nadirHb 80-90 deltaHb 30-40% | 2.551249 | 1.480332 | 0.34947 | 18.62496 | 1.614136 | 0.9662 |
| nadirHb 80-90 deltaHb 20-30% / nadirHb 50-60 deltaHb 30-40% | 0.116712 | 0.077356 | 0.012049 | 1.130553 | -3.2409 | 0.0879 |
| nadirHb 80-90 deltaHb 20-30% / nadirHb 60-70 deltaHb 30-40% | 0.265502 | 0.130529 | 0.049268 | 1.430765 | -2.69742 | 0.3319 |
| nadirHb 80-90 deltaHb 20-30% / nadirHb 70-80 deltaHb 30-40% | 0.603978 | 0.259524 | 0.138573 | 2.632474 | -1.17344 | 0.9987 |
| nadirHb 80-90 deltaHb 20-30% / nadirHb 80-90 deltaHb 30-40% | 1.373959 | 0.711415 | 0.233108 | 8.098257 | 0.613569 | 1.0000 |
| nadirHb 50-60 deltaHb 30-40% / nadirHb 60-70 deltaHb 30-40% | 2.27485 | 0.603007 | 0.917369 | 5.641071 | 3.100678 | 0.1299 |
| nadirHb 50-60 deltaHb 30-40% / nadirHb 70-80 deltaHb 30-40% | 5.174941 | 2.743501 | 0.841565 | 31.82168 | 3.100678 | 0.1299 |
| nadirHb 50-60 deltaHb 30-40% / nadirHb 80-90 deltaHb 30-40% | 11.77221 | 9.361578 | 0.772025 | 179.5083 | 3.100678 | 0.1299 |
| nadirHb 60-70 deltaHb 30-40% / nadirHb 70-80 deltaHb 30-40% | 2.27485 | 0.603007 | 0.917369 | 5.641071 | 3.100678 | 0.1299 |
| nadirHb 60-70 deltaHb 30-40% / nadirHb 80-90 deltaHb 30-40% | 5.174941 | 2.743501 | 0.841565 | 31.82168 | 3.100678 | 0.1299 |
| nadirHb 70-80 deltaHb 30-40% / nadirHb 80-90 deltaHb 30-40% | 2.27485 | 0.603007 | 0.917369 | 5.641071 | 3.100678 | 0.1299 |

# **Table S7.** Prediction of the need for urgent intervention in all participants – subgroup comparisons.

| **Comparison** | **Odds ratio** | **Standard error** | **Lower Confidence Interval** | **Upper Confidence Interval** | **Z ratio** | **P-value** |
| --- | --- | --- | --- | --- | --- | --- |
| nadirHb 50-60 deltaHb 0-10% / nadirHb 60-70 deltaHb 0-10% | 1.439252 | 0.176893 | 0.944634 | 2.192855 | 2.962613 | 0.1849 |
| nadirHb 50-60 deltaHb 0-10% / nadirHb 70-80 deltaHb 0-10% | 2.071445 | 0.509186 | 0.892333 | 4.808615 | 2.962613 | 0.1849 |
| nadirHb 50-60 deltaHb 0-10% / nadirHb 80-90 deltaHb 0-10% | 2.98133 | 1.099271 | 0.842927 | 10.5446 | 2.962613 | 0.1849 |
| nadirHb 50-60 deltaHb 0-10% / nadirHb 50-60 deltaHb 10-20% | 0.820929 | 0.10392 | 0.532051 | 1.266655 | -1.55874 | 0.9753 |
| nadirHb 50-60 deltaHb 0-10% / nadirHb 60-70 deltaHb 10-20% | 1.17333 | 0.212455 | 0.630962 | 2.181909 | 0.88278 | 1.0000 |
| nadirHb 50-60 deltaHb 0-10% / nadirHb 70-80 deltaHb 10-20% | 1.677005 | 0.427997 | 0.699515 | 4.020423 | 2.025779 | 0.8111 |
| nadirHb 50-60 deltaHb 0-10% / nadirHb 80-90 deltaHb 10-20% | 2.396894 | 0.805975 | 0.757403 | 7.585264 | 2.599711 | 0.3985 |
| nadirHb 50-60 deltaHb 0-10% / nadirHb 50-60 deltaHb 20-30% | 0.673924 | 0.170622 | 0.283078 | 1.604414 | -1.55874 | 0.9753 |
| nadirHb 50-60 deltaHb 0-10% / nadirHb 60-70 deltaHb 20-30% | 0.95654 | 0.265346 | 0.369789 | 2.474299 | -0.16017 | 1.0000 |
| nadirHb 50-60 deltaHb 0-10% / nadirHb 70-80 deltaHb 20-30% | 1.357674 | 0.43575 | 0.452111 | 4.077046 | 0.952701 | 0.9999 |
| nadirHb 50-60 deltaHb 0-10% / nadirHb 80-90 deltaHb 20-30% | 1.927025 | 0.726867 | 0.52924 | 7.016527 | 1.739087 | 0.9366 |
| nadirHb 50-60 deltaHb 0-10% / nadirHb 50-60 deltaHb 30-40% | 0.553244 | 0.210103 | 0.150612 | 2.032239 | -1.55874 | 0.9753 |
| nadirHb 50-60 deltaHb 0-10% / nadirHb 60-70 deltaHb 30-40% | 0.779806 | 0.299593 | 0.209097 | 2.908211 | -0.64736 | 1.0000 |
| nadirHb 50-60 deltaHb 0-10% / nadirHb 70-80 deltaHb 30-40% | 1.099148 | 0.458561 | 0.263212 | 4.589944 | 0.226598 | 1.0000 |
| nadirHb 50-60 deltaHb 0-10% / nadirHb 80-90 deltaHb 30-40% | 1.549266 | 0.732553 | 0.306608 | 7.828309 | 0.925858 | 0.9999 |
| nadirHb 60-70 deltaHb 0-10% / nadirHb 70-80 deltaHb 0-10% | 1.439252 | 0.176893 | 0.944634 | 2.192855 | 2.962613 | 0.1849 |
| nadirHb 60-70 deltaHb 0-10% / nadirHb 80-90 deltaHb 0-10% | 2.071445 | 0.509186 | 0.892333 | 4.808615 | 2.962613 | 0.1849 |
| nadirHb 60-70 deltaHb 0-10% / nadirHb 50-60 deltaHb 10-20% | 0.570386 | 0.072863 | 0.368213 | 0.883565 | -4.3951 | 0.0012 |
| nadirHb 60-70 deltaHb 0-10% / nadirHb 60-70 deltaHb 10-20% | 0.815236 | 0.093813 | 0.549619 | 1.209218 | -1.77517 | 0.9254 |
| nadirHb 60-70 deltaHb 0-10% / nadirHb 70-80 deltaHb 10-20% | 1.165192 | 0.186986 | 0.672392 | 2.01917 | 0.952701 | 0.9999 |
| nadirHb 60-70 deltaHb 0-10% / nadirHb 80-90 deltaHb 10-20% | 1.665375 | 0.386459 | 0.752028 | 3.687991 | 2.197968 | 0.6988 |
| nadirHb 60-70 deltaHb 0-10% / nadirHb 50-60 deltaHb 20-30% | 0.468246 | 0.104262 | 0.218358 | 1.004109 | -3.40763 | 0.0530 |
| nadirHb 60-70 deltaHb 0-10% / nadirHb 60-70 deltaHb 20-30% | 0.66461 | 0.15296 | 0.302081 | 1.462209 | -1.77517 | 0.9254 |
| nadirHb 60-70 deltaHb 0-10% / nadirHb 70-80 deltaHb 20-30% | 0.943319 | 0.248802 | 0.382138 | 2.328615 | -0.22123 | 1.0000 |
| nadirHb 60-70 deltaHb 0-10% / nadirHb 80-90 deltaHb 20-30% | 1.338908 | 0.422058 | 0.454698 | 3.942563 | 0.925858 | 0.9999 |
| nadirHb 60-70 deltaHb 0-10% / nadirHb 50-60 deltaHb 30-40% | 0.384397 | 0.130294 | 0.12035 | 1.227767 | -2.82066 | 0.2569 |
| nadirHb 60-70 deltaHb 0-10% / nadirHb 60-70 deltaHb 30-40% | 0.541814 | 0.187048 | 0.16603 | 1.768131 | -1.77517 | 0.9254 |
| nadirHb 60-70 deltaHb 0-10% / nadirHb 70-80 deltaHb 30-40% | 0.763694 | 0.292349 | 0.205748 | 2.834683 | -0.70424 | 1.0000 |
| nadirHb 60-70 deltaHb 0-10% / nadirHb 80-90 deltaHb 30-40% | 1.076439 | 0.477745 | 0.235301 | 4.924411 | 0.165964 | 1.0000 |
| nadirHb 70-80 deltaHb 0-10% / nadirHb 80-90 deltaHb 0-10% | 1.439252 | 0.176893 | 0.944634 | 2.192855 | 2.962613 | 0.1849 |
| nadirHb 70-80 deltaHb 0-10% / nadirHb 50-60 deltaHb 10-20% | 0.396307 | 0.085756 | 0.188828 | 0.831759 | -4.27735 | 0.0020 |
| nadirHb 70-80 deltaHb 0-10% / nadirHb 60-70 deltaHb 10-20% | 0.56643 | 0.087586 | 0.333483 | 0.962098 | -3.67592 | 0.0216 |
| nadirHb 70-80 deltaHb 0-10% / nadirHb 70-80 deltaHb 10-20% | 0.809582 | 0.104253 | 0.520784 | 1.258533 | -1.64037 | 0.9611 |
| nadirHb 70-80 deltaHb 0-10% / nadirHb 80-90 deltaHb 10-20% | 1.157112 | 0.182376 | 0.674313 | 1.985589 | 0.925858 | 0.9999 |
| nadirHb 70-80 deltaHb 0-10% / nadirHb 50-60 deltaHb 20-30% | 0.32534 | 0.08312 | 0.135581 | 0.780687 | -4.3951 | 0.0012 |
| nadirHb 70-80 deltaHb 0-10% / nadirHb 60-70 deltaHb 20-30% | 0.461774 | 0.112353 | 0.200637 | 1.062794 | -3.17573 | 0.1058 |
| nadirHb 70-80 deltaHb 0-10% / nadirHb 70-80 deltaHb 20-30% | 0.655423 | 0.168802 | 0.271216 | 1.583905 | -1.64037 | 0.9611 |
| nadirHb 70-80 deltaHb 0-10% / nadirHb 80-90 deltaHb 20-30% | 0.930281 | 0.273871 | 0.339296 | 2.550643 | -0.24548 | 1.0000 |
| nadirHb 70-80 deltaHb 0-10% / nadirHb 50-60 deltaHb 30-40% | 0.267081 | 0.090875 | 0.083249 | 0.856856 | -3.88007 | 0.0102 |
| nadirHb 70-80 deltaHb 0-10% / nadirHb 60-70 deltaHb 30-40% | 0.376455 | 0.130935 | 0.114341 | 1.239432 | -2.80888 | 0.2636 |
| nadirHb 70-80 deltaHb 0-10% / nadirHb 70-80 deltaHb 30-40% | 0.530619 | 0.204989 | 0.141245 | 1.993396 | -1.64037 | 0.9611 |
| nadirHb 70-80 deltaHb 0-10% / nadirHb 80-90 deltaHb 30-40% | 0.747916 | 0.33497 | 0.161236 | 3.469319 | -0.64855 | 1.0000 |
| nadirHb 80-90 deltaHb 0-10% / nadirHb 50-60 deltaHb 10-20% | 0.275357 | 0.090299 | 0.089528 | 0.846899 | -3.93277 | 0.0083 |
| nadirHb 80-90 deltaHb 0-10% / nadirHb 60-70 deltaHb 10-20% | 0.393559 | 0.100175 | 0.164544 | 0.941323 | -3.66361 | 0.0225 |
| nadirHb 80-90 deltaHb 0-10% / nadirHb 70-80 deltaHb 10-20% | 0.562502 | 0.109108 | 0.289411 | 1.093287 | -2.96624 | 0.1833 |
| nadirHb 80-90 deltaHb 0-10% / nadirHb 80-90 deltaHb 10-20% | 0.803968 | 0.12975 | 0.462497 | 1.397552 | -1.35201 | 0.9939 |
| nadirHb 80-90 deltaHb 0-10% / nadirHb 50-60 deltaHb 20-30% | 0.226048 | 0.075372 | 0.072124 | 0.708467 | -4.45969 | 0.0009 |
| nadirHb 80-90 deltaHb 0-10% / nadirHb 60-70 deltaHb 20-30% | 0.320843 | 0.099223 | 0.111211 | 0.925633 | -3.67592 | 0.0216 |
| nadirHb 80-90 deltaHb 0-10% / nadirHb 70-80 deltaHb 20-30% | 0.455392 | 0.139106 | 0.159913 | 1.296844 | -2.57509 | 0.4161 |
| nadirHb 80-90 deltaHb 0-10% / nadirHb 80-90 deltaHb 20-30% | 0.646364 | 0.208629 | 0.213904 | 1.953152 | -1.35201 | 0.9939 |
| nadirHb 80-90 deltaHb 0-10% / nadirHb 50-60 deltaHb 30-40% | 0.18557 | 0.071115 | 0.049923 | 0.689788 | -4.3951 | 0.0012 |
| nadirHb 80-90 deltaHb 0-10% / nadirHb 60-70 deltaHb 30-40% | 0.261563 | 0.102302 | 0.06849 | 0.998911 | -3.42883 | 0.0496 |
| nadirHb 80-90 deltaHb 0-10% / nadirHb 70-80 deltaHb 30-40% | 0.368677 | 0.157351 | 0.08543 | 1.591051 | -2.33795 | 0.5950 |
| nadirHb 80-90 deltaHb 0-10% / nadirHb 80-90 deltaHb 30-40% | 0.519656 | 0.251597 | 0.09893 | 2.729632 | -1.35201 | 0.9939 |
| nadirHb 50-60 deltaHb 10-20% / nadirHb 60-70 deltaHb 10-20% | 1.42927 | 0.126175 | 1.056241 | 1.934042 | 4.045847 | 0.0053 |
| nadirHb 50-60 deltaHb 10-20% / nadirHb 70-80 deltaHb 10-20% | 2.042814 | 0.360676 | 1.115644 | 3.740519 | 4.045847 | 0.0053 |
| nadirHb 50-60 deltaHb 10-20% / nadirHb 80-90 deltaHb 10-20% | 2.919733 | 0.773255 | 1.178389 | 7.234321 | 4.045847 | 0.0053 |
| nadirHb 50-60 deltaHb 10-20% / nadirHb 50-60 deltaHb 20-30% | 0.820929 | 0.10392 | 0.532051 | 1.266655 | -1.55874 | 0.9753 |
| nadirHb 50-60 deltaHb 10-20% / nadirHb 60-70 deltaHb 20-30% | 1.165192 | 0.186986 | 0.672392 | 2.01917 | 0.952701 | 0.9999 |
| nadirHb 50-60 deltaHb 10-20% / nadirHb 70-80 deltaHb 20-30% | 1.653826 | 0.364921 | 0.776567 | 3.522093 | 2.280017 | 0.6388 |
| nadirHb 50-60 deltaHb 10-20% / nadirHb 80-90 deltaHb 20-30% | 2.347371 | 0.68368 | 0.865411 | 6.367094 | 2.929735 | 0.2002 |
| nadirHb 50-60 deltaHb 10-20% / nadirHb 50-60 deltaHb 30-40% | 0.673924 | 0.170622 | 0.283078 | 1.604414 | -1.55874 | 0.9753 |
| nadirHb 50-60 deltaHb 10-20% / nadirHb 60-70 deltaHb 30-40% | 0.949907 | 0.251675 | 0.383233 | 2.354499 | -0.19397 | 1.0000 |
| nadirHb 50-60 deltaHb 10-20% / nadirHb 70-80 deltaHb 30-40% | 1.338908 | 0.422058 | 0.454698 | 3.942563 | 0.925858 | 0.9999 |
| nadirHb 50-60 deltaHb 10-20% / nadirHb 80-90 deltaHb 30-40% | 1.887211 | 0.734826 | 0.497131 | 7.16424 | 1.631091 | 0.9630 |
| nadirHb 60-70 deltaHb 10-20% / nadirHb 70-80 deltaHb 10-20% | 1.42927 | 0.126175 | 1.056241 | 1.934042 | 4.045847 | 0.0053 |
| nadirHb 60-70 deltaHb 10-20% / nadirHb 80-90 deltaHb 10-20% | 2.042814 | 0.360676 | 1.115644 | 3.740519 | 4.045847 | 0.0053 |
| nadirHb 60-70 deltaHb 10-20% / nadirHb 50-60 deltaHb 20-30% | 0.574369 | 0.069991 | 0.378336 | 0.871976 | -4.55025 | 0.0006 |
| nadirHb 60-70 deltaHb 10-20% / nadirHb 60-70 deltaHb 20-30% | 0.815236 | 0.093813 | 0.549619 | 1.209218 | -1.77517 | 0.9254 |
| nadirHb 60-70 deltaHb 10-20% / nadirHb 70-80 deltaHb 20-30% | 1.157112 | 0.182376 | 0.674313 | 1.985589 | 0.925858 | 0.9999 |
| nadirHb 60-70 deltaHb 10-20% / nadirHb 80-90 deltaHb 20-30% | 1.642356 | 0.365947 | 0.76548 | 3.523717 | 2.226623 | 0.6782 |
| nadirHb 60-70 deltaHb 10-20% / nadirHb 50-60 deltaHb 30-40% | 0.471516 | 0.109525 | 0.212756 | 1.044988 | -3.23659 | 0.0890 |
| nadirHb 60-70 deltaHb 10-20% / nadirHb 60-70 deltaHb 30-40% | 0.66461 | 0.15296 | 0.302081 | 1.462209 | -1.77517 | 0.9254 |
| nadirHb 60-70 deltaHb 10-20% / nadirHb 70-80 deltaHb 30-40% | 0.936777 | 0.256642 | 0.366441 | 2.394798 | -0.23839 | 1.0000 |
| nadirHb 60-70 deltaHb 10-20% / nadirHb 80-90 deltaHb 30-40% | 1.320402 | 0.457809 | 0.402548 | 4.331058 | 0.801616 | 1.0000 |
| nadirHb 70-80 deltaHb 10-20% / nadirHb 80-90 deltaHb 10-20% | 1.42927 | 0.126175 | 1.056241 | 1.934042 | 4.045847 | 0.0053 |
| nadirHb 70-80 deltaHb 10-20% / nadirHb 50-60 deltaHb 20-30% | 0.401862 | 0.068741 | 0.223645 | 0.722094 | -5.32951 | 0.0000 |
| nadirHb 70-80 deltaHb 10-20% / nadirHb 60-70 deltaHb 20-30% | 0.570386 | 0.072863 | 0.368213 | 0.883565 | -4.3951 | 0.0012 |
| nadirHb 70-80 deltaHb 10-20% / nadirHb 70-80 deltaHb 20-30% | 0.809582 | 0.104253 | 0.520784 | 1.258533 | -1.64037 | 0.9611 |
| nadirHb 70-80 deltaHb 10-20% / nadirHb 80-90 deltaHb 20-30% | 1.149087 | 0.199206 | 0.634467 | 2.081119 | 0.801616 | 1.0000 |
| nadirHb 70-80 deltaHb 10-20% / nadirHb 50-60 deltaHb 30-40% | 0.3299 | 0.080402 | 0.143138 | 0.760342 | -4.55025 | 0.0006 |
| nadirHb 70-80 deltaHb 10-20% / nadirHb 60-70 deltaHb 30-40% | 0.464999 | 0.105348 | 0.213972 | 1.010526 | -3.37982 | 0.0578 |
| nadirHb 70-80 deltaHb 10-20% / nadirHb 70-80 deltaHb 30-40% | 0.655423 | 0.168802 | 0.271216 | 1.583905 | -1.64037 | 0.9611 |
| nadirHb 70-80 deltaHb 10-20% / nadirHb 80-90 deltaHb 30-40% | 0.923829 | 0.298506 | 0.305366 | 2.79488 | -0.2452 | 1.0000 |
| nadirHb 80-90 deltaHb 10-20% / nadirHb 50-60 deltaHb 20-30% | 0.281166 | 0.068444 | 0.122113 | 0.647386 | -5.21224 | 0.0000 |
| nadirHb 80-90 deltaHb 10-20% / nadirHb 60-70 deltaHb 20-30% | 0.399075 | 0.074639 | 0.210266 | 0.757425 | -4.91152 | 0.0001 |
| nadirHb 80-90 deltaHb 10-20% / nadirHb 70-80 deltaHb 20-30% | 0.56643 | 0.087586 | 0.333483 | 0.962098 | -3.67592 | 0.0216 |
| nadirHb 80-90 deltaHb 10-20% / nadirHb 80-90 deltaHb 20-30% | 0.803968 | 0.12975 | 0.462497 | 1.397552 | -1.35201 | 0.9939 |
| nadirHb 80-90 deltaHb 10-20% / nadirHb 50-60 deltaHb 30-40% | 0.230817 | 0.065459 | 0.087358 | 0.609862 | -5.1698 | 0.0000 |
| nadirHb 80-90 deltaHb 10-20% / nadirHb 60-70 deltaHb 30-40% | 0.32534 | 0.08312 | 0.135581 | 0.780687 | -4.3951 | 0.0012 |
| nadirHb 80-90 deltaHb 10-20% / nadirHb 70-80 deltaHb 30-40% | 0.458572 | 0.124062 | 0.181497 | 1.158635 | -2.88179 | 0.2240 |
| nadirHb 80-90 deltaHb 10-20% / nadirHb 80-90 deltaHb 30-40% | 0.646364 | 0.208629 | 0.213904 | 1.953152 | -1.35201 | 0.9939 |
| nadirHb 50-60 deltaHb 20-30% / nadirHb 60-70 deltaHb 20-30% | 1.419358 | 0.115338 | 1.074443 | 1.874997 | 4.309653 | 0.0018 |
| nadirHb 50-60 deltaHb 20-30% / nadirHb 70-80 deltaHb 20-30% | 2.014578 | 0.327412 | 1.154429 | 3.515613 | 4.309653 | 0.0018 |
| nadirHb 50-60 deltaHb 20-30% / nadirHb 80-90 deltaHb 20-30% | 2.859408 | 0.697072 | 1.240368 | 6.591764 | 4.309653 | 0.0018 |
| nadirHb 50-60 deltaHb 20-30% / nadirHb 50-60 deltaHb 30-40% | 0.820929 | 0.10392 | 0.532051 | 1.266655 | -1.55874 | 0.9753 |
| nadirHb 50-60 deltaHb 20-30% / nadirHb 60-70 deltaHb 30-40% | 1.157112 | 0.182376 | 0.674313 | 1.985589 | 0.925858 | 0.9999 |
| nadirHb 50-60 deltaHb 20-30% / nadirHb 70-80 deltaHb 30-40% | 1.630967 | 0.388469 | 0.72119 | 3.68842 | 2.053768 | 0.7945 |
| nadirHb 50-60 deltaHb 20-30% / nadirHb 80-90 deltaHb 30-40% | 2.298872 | 0.768294 | 0.731557 | 7.224065 | 2.490745 | 0.4784 |
| nadirHb 60-70 deltaHb 20-30% / nadirHb 70-80 deltaHb 20-30% | 1.419358 | 0.115338 | 1.074443 | 1.874997 | 4.309653 | 0.0018 |
| nadirHb 60-70 deltaHb 20-30% / nadirHb 80-90 deltaHb 20-30% | 2.014578 | 0.327412 | 1.154429 | 3.515613 | 4.309653 | 0.0018 |
| nadirHb 60-70 deltaHb 20-30% / nadirHb 50-60 deltaHb 30-40% | 0.57838 | 0.080773 | 0.358444 | 0.933267 | -3.92058 | 0.0087 |
| nadirHb 60-70 deltaHb 20-30% / nadirHb 60-70 deltaHb 30-40% | 0.815236 | 0.093813 | 0.549619 | 1.209218 | -1.77517 | 0.9254 |
| nadirHb 60-70 deltaHb 20-30% / nadirHb 70-80 deltaHb 30-40% | 1.149087 | 0.199206 | 0.634467 | 2.081119 | 0.801616 | 1.0000 |
| nadirHb 60-70 deltaHb 20-30% / nadirHb 80-90 deltaHb 30-40% | 1.619656 | 0.428328 | 0.654538 | 4.00784 | 1.823415 | 0.9084 |
| nadirHb 70-80 deltaHb 20-30% / nadirHb 80-90 deltaHb 20-30% | 1.419358 | 0.115338 | 1.074443 | 1.874997 | 4.309653 | 0.0018 |
| nadirHb 70-80 deltaHb 20-30% / nadirHb 50-60 deltaHb 30-40% | 0.407494 | 0.077518 | 0.21236 | 0.781933 | -4.71913 | 0.0003 |
| nadirHb 70-80 deltaHb 20-30% / nadirHb 60-70 deltaHb 30-40% | 0.574369 | 0.069991 | 0.378336 | 0.871976 | -4.55025 | 0.0006 |
| nadirHb 70-80 deltaHb 20-30% / nadirHb 70-80 deltaHb 30-40% | 0.809582 | 0.104253 | 0.520784 | 1.258533 | -1.64037 | 0.9611 |
| nadirHb 70-80 deltaHb 20-30% / nadirHb 80-90 deltaHb 30-40% | 1.141118 | 0.23215 | 0.568367 | 2.29104 | 0.648881 | 1.0000 |
| nadirHb 80-90 deltaHb 20-30% / nadirHb 50-60 deltaHb 30-40% | 0.287098 | 0.073801 | 0.119 | 0.692646 | -4.85464 | 0.0001 |
| nadirHb 80-90 deltaHb 20-30% / nadirHb 60-70 deltaHb 30-40% | 0.404668 | 0.069696 | 0.224304 | 0.730064 | -5.25282 | 0.0000 |
| nadirHb 80-90 deltaHb 20-30% / nadirHb 70-80 deltaHb 30-40% | 0.570386 | 0.072863 | 0.368213 | 0.883565 | -4.3951 | 0.0012 |
| nadirHb 80-90 deltaHb 20-30% / nadirHb 80-90 deltaHb 30-40% | 0.803968 | 0.12975 | 0.462497 | 1.397552 | -1.35201 | 0.9939 |
| nadirHb 50-60 deltaHb 30-40% / nadirHb 60-70 deltaHb 30-40% | 1.409515 | 0.151391 | 0.975571 | 2.036483 | 3.195769 | 0.1000 |
| nadirHb 50-60 deltaHb 30-40% / nadirHb 70-80 deltaHb 30-40% | 1.986733 | 0.426775 | 0.951738 | 4.147262 | 3.195769 | 0.1000 |
| nadirHb 50-60 deltaHb 30-40% / nadirHb 80-90 deltaHb 30-40% | 2.80033 | 0.902319 | 0.928488 | 8.445828 | 3.195769 | 0.1000 |
| nadirHb 60-70 deltaHb 30-40% / nadirHb 70-80 deltaHb 30-40% | 1.409515 | 0.151391 | 0.975571 | 2.036483 | 3.195769 | 0.1000 |
| nadirHb 60-70 deltaHb 30-40% / nadirHb 80-90 deltaHb 30-40% | 1.986733 | 0.426775 | 0.951738 | 4.147262 | 3.195769 | 0.1000 |
| nadirHb 70-80 deltaHb 30-40% / nadirHb 80-90 deltaHb 30-40% | 1.409515 | 0.151391 | 0.975571 | 2.036483 | 3.195769 | 0.1000 |

# **Table S8.** Prediction of the need for urgent intervention in the red blood cell transfused population – subgroup comparisons.

| **Comparison** | **Odds ratio** | **Standard error** | **Lower Confidece Interval** | **Upper Confidence Interval** | **Z ratio** | **P-value** |
| --- | --- | --- | --- | --- | --- | --- |
| nadirHb 50-60 deltaHb 0-10% / nadirHb 60-70 deltaHb 0-10% | 1.346358 | 0.304071 | 0.621041 | 2.918779 | 1.316833 | 0.9954 |
| nadirHb 50-60 deltaHb 0-10% / nadirHb 70-80 deltaHb 0-10% | 1.81268 | 0.818778 | 0.385691 | 8.519269 | 1.316833 | 0.9954 |
| nadirHb 50-60 deltaHb 0-10% / nadirHb 80-90 deltaHb 0-10% | 2.440516 | 1.653552 | 0.23953 | 24.86586 | 1.316833 | 0.9954 |
| nadirHb 50-60 deltaHb 0-10% / nadirHb 50-60 deltaHb 10-20% | 0.809417 | 0.104977 | 0.519036 | 1.262254 | -1.6303 | 0.9631 |
| nadirHb 50-60 deltaHb 0-10% / nadirHb 60-70 deltaHb 10-20% | 1.076918 | 0.26555 | 0.462688 | 2.506552 | 0.30052 | 1.0000 |
| nadirHb 50-60 deltaHb 0-10% / nadirHb 70-80 deltaHb 10-20% | 1.432824 | 0.572241 | 0.364713 | 5.629036 | 0.900516 | 0.9999 |
| nadirHb 50-60 deltaHb 0-10% / nadirHb 80-90 deltaHb 10-20% | 1.906353 | 1.066404 | 0.280462 | 12.95785 | 1.153375 | 0.9989 |
| nadirHb 50-60 deltaHb 0-10% / nadirHb 50-60 deltaHb 20-30% | 0.655156 | 0.169941 | 0.269399 | 1.593286 | -1.6303 | 0.9631 |
| nadirHb 50-60 deltaHb 0-10% / nadirHb 60-70 deltaHb 20-30% | 0.861399 | 0.276396 | 0.286933 | 2.586004 | -0.46498 | 1.0000 |
| nadirHb 50-60 deltaHb 0-10% / nadirHb 70-80 deltaHb 20-30% | 1.132569 | 0.470402 | 0.272944 | 4.699537 | 0.299726 | 1.0000 |
| nadirHb 50-60 deltaHb 0-10% / nadirHb 80-90 deltaHb 20-30% | 1.489103 | 0.782221 | 0.246224 | 9.005743 | 0.757998 | 1.0000 |
| nadirHb 50-60 deltaHb 0-10% / nadirHb 50-60 deltaHb 30-40% | 0.530294 | 0.206329 | 0.139828 | 2.011132 | -1.6303 | 0.9631 |
| nadirHb 50-60 deltaHb 0-10% / nadirHb 60-70 deltaHb 30-40% | 0.689012 | 0.290259 | 0.162711 | 2.917679 | -0.88423 | 1.0000 |
| nadirHb 50-60 deltaHb 0-10% / nadirHb 70-80 deltaHb 30-40% | 0.895234 | 0.441342 | 0.165352 | 4.846894 | -0.22449 | 1.0000 |
| nadirHb 50-60 deltaHb 0-10% / nadirHb 80-90 deltaHb 30-40% | 1.163178 | 0.68628 | 0.154089 | 8.780523 | 0.256194 | 1.0000 |
| nadirHb 60-70 deltaHb 0-10% / nadirHb 70-80 deltaHb 0-10% | 1.346358 | 0.304071 | 0.621041 | 2.918779 | 1.316833 | 0.9954 |
| nadirHb 60-70 deltaHb 0-10% / nadirHb 80-90 deltaHb 0-10% | 1.81268 | 0.818778 | 0.385691 | 8.519269 | 1.316833 | 0.9954 |
| nadirHb 60-70 deltaHb 0-10% / nadirHb 50-60 deltaHb 10-20% | 0.60119 | 0.126032 | 0.293149 | 1.232918 | -2.42725 | 0.5267 |
| nadirHb 60-70 deltaHb 0-10% / nadirHb 60-70 deltaHb 10-20% | 0.799875 | 0.10174 | 0.517329 | 1.236736 | -1.75557 | 0.9316 |
| nadirHb 60-70 deltaHb 0-10% / nadirHb 70-80 deltaHb 10-20% | 1.064222 | 0.221008 | 0.522441 | 2.167842 | 0.299726 | 1.0000 |
| nadirHb 60-70 deltaHb 0-10% / nadirHb 80-90 deltaHb 10-20% | 1.415933 | 0.500194 | 0.422106 | 4.749675 | 0.984508 | 0.9998 |
| nadirHb 60-70 deltaHb 0-10% / nadirHb 50-60 deltaHb 20-30% | 0.486613 | 0.129233 | 0.195898 | 1.208754 | -2.71216 | 0.3224 |
| nadirHb 60-70 deltaHb 0-10% / nadirHb 60-70 deltaHb 20-30% | 0.6398 | 0.162759 | 0.26763 | 1.529515 | -1.75557 | 0.9316 |
| nadirHb 60-70 deltaHb 0-10% / nadirHb 70-80 deltaHb 20-30% | 0.841209 | 0.256224 | 0.296279 | 2.3884 | -0.5677 | 1.0000 |
| nadirHb 60-70 deltaHb 0-10% / nadirHb 80-90 deltaHb 20-30% | 1.106023 | 0.435039 | 0.287418 | 4.256117 | 0.256194 | 1.0000 |
| nadirHb 60-70 deltaHb 0-10% / nadirHb 50-60 deltaHb 30-40% | 0.393873 | 0.142425 | 0.114111 | 1.35952 | -2.57667 | 0.4150 |
| nadirHb 60-70 deltaHb 0-10% / nadirHb 60-70 deltaHb 30-40% | 0.51176 | 0.19528 | 0.138453 | 1.891605 | -1.75557 | 0.9316 |
| nadirHb 60-70 deltaHb 0-10% / nadirHb 70-80 deltaHb 30-40% | 0.66493 | 0.29733 | 0.143695 | 3.076869 | -0.91259 | 0.9999 |
| nadirHb 60-70 deltaHb 0-10% / nadirHb 80-90 deltaHb 30-40% | 0.863944 | 0.468277 | 0.134898 | 5.533071 | -0.26982 | 1.0000 |
| nadirHb 70-80 deltaHb 0-10% / nadirHb 80-90 deltaHb 0-10% | 1.346358 | 0.304071 | 0.621041 | 2.918779 | 1.316833 | 0.9954 |
| nadirHb 70-80 deltaHb 0-10% / nadirHb 50-60 deltaHb 10-20% | 0.44653 | 0.185775 | 0.107355 | 1.857298 | -1.93791 | 0.8585 |
| nadirHb 70-80 deltaHb 0-10% / nadirHb 60-70 deltaHb 10-20% | 0.594103 | 0.161141 | 0.234579 | 1.504641 | -1.91976 | 0.8673 |
| nadirHb 70-80 deltaHb 0-10% / nadirHb 70-80 deltaHb 10-20% | 0.790445 | 0.134059 | 0.442102 | 1.413256 | -1.38655 | 0.9921 |
| nadirHb 70-80 deltaHb 0-10% / nadirHb 80-90 deltaHb 10-20% | 1.051676 | 0.206831 | 0.536114 | 2.063036 | 0.256194 | 1.0000 |
| nadirHb 70-80 deltaHb 0-10% / nadirHb 50-60 deltaHb 20-30% | 0.361429 | 0.151539 | 0.085937 | 1.520088 | -2.42725 | 0.5267 |
| nadirHb 70-80 deltaHb 0-10% / nadirHb 60-70 deltaHb 20-30% | 0.475208 | 0.170338 | 0.139169 | 1.622644 | -2.07562 | 0.7810 |
| nadirHb 70-80 deltaHb 0-10% / nadirHb 70-80 deltaHb 20-30% | 0.624804 | 0.211933 | 0.195454 | 1.997294 | -1.38655 | 0.9921 |
| nadirHb 70-80 deltaHb 0-10% / nadirHb 80-90 deltaHb 20-30% | 0.821492 | 0.302399 | 0.23275 | 2.899459 | -0.53417 | 1.0000 |
| nadirHb 70-80 deltaHb 0-10% / nadirHb 50-60 deltaHb 30-40% | 0.292547 | 0.134742 | 0.060379 | 1.417433 | -2.66864 | 0.3509 |
| nadirHb 70-80 deltaHb 0-10% / nadirHb 60-70 deltaHb 30-40% | 0.380107 | 0.17656 | 0.077405 | 1.866554 | -2.08246 | 0.7767 |
| nadirHb 70-80 deltaHb 0-10% / nadirHb 70-80 deltaHb 30-40% | 0.493873 | 0.251282 | 0.086411 | 2.822688 | -1.38655 | 0.9921 |
| nadirHb 70-80 deltaHb 0-10% / nadirHb 80-90 deltaHb 30-40% | 0.64169 | 0.374982 | 0.086664 | 4.751281 | -0.7592 | 1.0000 |
| nadirHb 80-90 deltaHb 0-10% / nadirHb 50-60 deltaHb 10-20% | 0.331658 | 0.210869 | 0.037555 | 2.928921 | -1.73584 | 0.9375 |
| nadirHb 80-90 deltaHb 0-10% / nadirHb 60-70 deltaHb 10-20% | 0.441266 | 0.212986 | 0.084436 | 2.306065 | -1.69496 | 0.9486 |
| nadirHb 80-90 deltaHb 0-10% / nadirHb 70-80 deltaHb 10-20% | 0.587099 | 0.200316 | 0.182407 | 1.889649 | -1.56087 | 0.9750 |
| nadirHb 80-90 deltaHb 0-10% / nadirHb 80-90 deltaHb 10-20% | 0.781127 | 0.182485 | 0.350848 | 1.739097 | -1.05736 | 0.9996 |
| nadirHb 80-90 deltaHb 0-10% / nadirHb 50-60 deltaHb 20-30% | 0.26845 | 0.16615 | 0.032208 | 2.237524 | -2.1248 | 0.7493 |
| nadirHb 80-90 deltaHb 0-10% / nadirHb 60-70 deltaHb 20-30% | 0.352958 | 0.191468 | 0.055027 | 2.263945 | -1.91976 | 0.8673 |
| nadirHb 80-90 deltaHb 0-10% / nadirHb 70-80 deltaHb 20-30% | 0.464069 | 0.227039 | 0.086825 | 2.480398 | -1.56923 | 0.9738 |
| nadirHb 80-90 deltaHb 0-10% / nadirHb 80-90 deltaHb 20-30% | 0.610159 | 0.285088 | 0.123094 | 3.024458 | -1.05736 | 0.9996 |
| nadirHb 80-90 deltaHb 0-10% / nadirHb 50-60 deltaHb 30-40% | 0.217288 | 0.136655 | 0.025192 | 1.874144 | -2.42725 | 0.5267 |
| nadirHb 80-90 deltaHb 0-10% / nadirHb 60-70 deltaHb 30-40% | 0.282322 | 0.175841 | 0.033421 | 2.384917 | -2.03055 | 0.8083 |
| nadirHb 80-90 deltaHb 0-10% / nadirHb 70-80 deltaHb 30-40% | 0.366821 | 0.237675 | 0.039846 | 3.376908 | -1.54782 | 0.9769 |
| nadirHb 80-90 deltaHb 0-10% / nadirHb 80-90 deltaHb 30-40% | 0.476611 | 0.334035 | 0.043187 | 5.259826 | -1.05736 | 0.9996 |
| nadirHb 50-60 deltaHb 10-20% / nadirHb 60-70 deltaHb 10-20% | 1.330486 | 0.22007 | 0.754921 | 2.344871 | 1.726329 | 0.9402 |
| nadirHb 50-60 deltaHb 10-20% / nadirHb 70-80 deltaHb 10-20% | 1.770193 | 0.585599 | 0.569906 | 5.498421 | 1.726329 | 0.9402 |
| nadirHb 50-60 deltaHb 10-20% / nadirHb 80-90 deltaHb 10-20% | 2.355217 | 1.168698 | 0.430234 | 12.89309 | 1.726329 | 0.9402 |
| nadirHb 50-60 deltaHb 10-20% / nadirHb 50-60 deltaHb 20-30% | 0.809417 | 0.104977 | 0.519036 | 1.262254 | -1.6303 | 0.9631 |
| nadirHb 50-60 deltaHb 10-20% / nadirHb 60-70 deltaHb 20-30% | 1.064222 | 0.221008 | 0.522441 | 2.167842 | 0.299726 | 1.0000 |
| nadirHb 50-60 deltaHb 10-20% / nadirHb 70-80 deltaHb 20-30% | 1.399241 | 0.449744 | 0.465209 | 4.208588 | 1.045143 | 0.9997 |
| nadirHb 50-60 deltaHb 10-20% / nadirHb 80-90 deltaHb 20-30% | 1.839723 | 0.817325 | 0.401537 | 8.429071 | 1.372187 | 0.9929 |
| nadirHb 50-60 deltaHb 10-20% / nadirHb 50-60 deltaHb 30-40% | 0.655156 | 0.169941 | 0.269399 | 1.593286 | -1.6303 | 0.9631 |
| nadirHb 50-60 deltaHb 10-20% / nadirHb 60-70 deltaHb 30-40% | 0.851245 | 0.257144 | 0.302403 | 2.396195 | -0.53316 | 1.0000 |
| nadirHb 50-60 deltaHb 10-20% / nadirHb 70-80 deltaHb 30-40% | 1.106023 | 0.435039 | 0.287418 | 4.256117 | 0.256194 | 1.0000 |
| nadirHb 50-60 deltaHb 10-20% / nadirHb 80-90 deltaHb 30-40% | 1.437057 | 0.729463 | 0.252462 | 8.179956 | 0.714323 | 1.0000 |
| nadirHb 60-70 deltaHb 10-20% / nadirHb 70-80 deltaHb 10-20% | 1.330486 | 0.22007 | 0.754921 | 2.344871 | 1.726329 | 0.9402 |
| nadirHb 60-70 deltaHb 10-20% / nadirHb 80-90 deltaHb 10-20% | 1.770193 | 0.585599 | 0.569906 | 5.498421 | 1.726329 | 0.9402 |
| nadirHb 60-70 deltaHb 10-20% / nadirHb 50-60 deltaHb 20-30% | 0.608362 | 0.100988 | 0.344483 | 1.074375 | -2.99388 | 0.1712 |
| nadirHb 60-70 deltaHb 10-20% / nadirHb 60-70 deltaHb 20-30% | 0.799875 | 0.10174 | 0.517329 | 1.236736 | -1.75557 | 0.9316 |
| nadirHb 60-70 deltaHb 10-20% / nadirHb 70-80 deltaHb 20-30% | 1.051676 | 0.206831 | 0.536114 | 2.063036 | 0.256194 | 1.0000 |
| nadirHb 60-70 deltaHb 10-20% / nadirHb 80-90 deltaHb 20-30% | 1.382745 | 0.426315 | 0.480843 | 3.976319 | 1.051117 | 0.9996 |
| nadirHb 60-70 deltaHb 10-20% / nadirHb 50-60 deltaHb 30-40% | 0.492418 | 0.122011 | 0.210698 | 1.150823 | -2.8591 | 0.2359 |
| nadirHb 60-70 deltaHb 10-20% / nadirHb 60-70 deltaHb 30-40% | 0.6398 | 0.162759 | 0.26763 | 1.529515 | -1.75557 | 0.9316 |
| nadirHb 60-70 deltaHb 10-20% / nadirHb 70-80 deltaHb 30-40% | 0.831292 | 0.27259 | 0.270303 | 2.556564 | -0.56349 | 1.0000 |
| nadirHb 60-70 deltaHb 10-20% / nadirHb 80-90 deltaHb 30-40% | 1.080099 | 0.470593 | 0.242767 | 4.805495 | 0.17685 | 1.0000 |
| nadirHb 70-80 deltaHb 10-20% / nadirHb 80-90 deltaHb 10-20% | 1.330486 | 0.22007 | 0.754921 | 2.344871 | 1.726329 | 0.9402 |
| nadirHb 70-80 deltaHb 10-20% / nadirHb 50-60 deltaHb 20-30% | 0.457248 | 0.139449 | 0.160834 | 1.29995 | -2.56588 | 0.4228 |
| nadirHb 70-80 deltaHb 10-20% / nadirHb 60-70 deltaHb 20-30% | 0.60119 | 0.126032 | 0.293149 | 1.232918 | -2.42725 | 0.5267 |
| nadirHb 70-80 deltaHb 10-20% / nadirHb 70-80 deltaHb 20-30% | 0.790445 | 0.134059 | 0.442102 | 1.413256 | -1.38655 | 0.9921 |
| nadirHb 70-80 deltaHb 10-20% / nadirHb 80-90 deltaHb 20-30% | 1.039278 | 0.226404 | 0.492714 | 2.192144 | 0.17685 | 1.0000 |
| nadirHb 70-80 deltaHb 10-20% / nadirHb 50-60 deltaHb 30-40% | 0.370104 | 0.122875 | 0.118669 | 1.154282 | -2.99388 | 0.1712 |
| nadirHb 70-80 deltaHb 10-20% / nadirHb 60-70 deltaHb 30-40% | 0.480877 | 0.146564 | 0.169254 | 1.366242 | -2.40216 | 0.5459 |
| nadirHb 70-80 deltaHb 10-20% / nadirHb 70-80 deltaHb 30-40% | 0.624804 | 0.211933 | 0.195454 | 1.997294 | -1.38655 | 0.9921 |
| nadirHb 70-80 deltaHb 10-20% / nadirHb 80-90 deltaHb 30-40% | 0.811808 | 0.341259 | 0.192301 | 3.427088 | -0.49597 | 1.0000 |
| nadirHb 80-90 deltaHb 10-20% / nadirHb 50-60 deltaHb 20-30% | 0.34367 | 0.158678 | 0.070656 | 1.6716 | -2.31327 | 0.6138 |
| nadirHb 80-90 deltaHb 10-20% / nadirHb 60-70 deltaHb 20-30% | 0.451857 | 0.16067 | 0.133639 | 1.527811 | -2.23408 | 0.6728 |
| nadirHb 80-90 deltaHb 10-20% / nadirHb 70-80 deltaHb 20-30% | 0.594103 | 0.161141 | 0.234579 | 1.504641 | -1.91976 | 0.8673 |
| nadirHb 80-90 deltaHb 10-20% / nadirHb 80-90 deltaHb 20-30% | 0.781127 | 0.182485 | 0.350848 | 1.739097 | -1.05736 | 0.9996 |
| nadirHb 80-90 deltaHb 10-20% / nadirHb 50-60 deltaHb 30-40% | 0.278172 | 0.128614 | 0.057065 | 1.355992 | -2.76739 | 0.2880 |
| nadirHb 80-90 deltaHb 10-20% / nadirHb 60-70 deltaHb 30-40% | 0.361429 | 0.151539 | 0.085937 | 1.520088 | -2.42725 | 0.5267 |
| nadirHb 80-90 deltaHb 10-20% / nadirHb 70-80 deltaHb 30-40% | 0.469606 | 0.197739 | 0.110971 | 1.987266 | -1.79508 | 0.9187 |
| nadirHb 80-90 deltaHb 10-20% / nadirHb 80-90 deltaHb 30-40% | 0.610159 | 0.285088 | 0.123094 | 3.024458 | -1.05736 | 0.9996 |
| nadirHb 50-60 deltaHb 20-30% / nadirHb 60-70 deltaHb 20-30% | 1.314801 | 0.17112 | 0.841803 | 2.053571 | 2.102866 | 0.7637 |
| nadirHb 50-60 deltaHb 20-30% / nadirHb 70-80 deltaHb 20-30% | 1.728702 | 0.449977 | 0.708632 | 4.217155 | 2.102866 | 0.7637 |
| nadirHb 50-60 deltaHb 20-30% / nadirHb 80-90 deltaHb 20-30% | 2.272899 | 0.887445 | 0.596528 | 8.660229 | 2.102866 | 0.7637 |
| nadirHb 50-60 deltaHb 20-30% / nadirHb 50-60 deltaHb 30-40% | 0.809417 | 0.104977 | 0.519036 | 1.262254 | -1.6303 | 0.9631 |
| nadirHb 50-60 deltaHb 20-30% / nadirHb 60-70 deltaHb 30-40% | 1.051676 | 0.206831 | 0.536114 | 2.063036 | 0.256194 | 1.0000 |
| nadirHb 50-60 deltaHb 20-30% / nadirHb 70-80 deltaHb 30-40% | 1.366444 | 0.432168 | 0.462388 | 4.038098 | 0.987161 | 0.9998 |
| nadirHb 50-60 deltaHb 20-30% / nadirHb 80-90 deltaHb 30-40% | 1.775422 | 0.795718 | 0.382333 | 8.244448 | 1.280805 | 0.9966 |
| nadirHb 60-70 deltaHb 20-30% / nadirHb 70-80 deltaHb 20-30% | 1.314801 | 0.17112 | 0.841803 | 2.053571 | 2.102866 | 0.7637 |
| nadirHb 60-70 deltaHb 20-30% / nadirHb 80-90 deltaHb 20-30% | 1.728702 | 0.449977 | 0.708632 | 4.217155 | 2.102866 | 0.7637 |
| nadirHb 60-70 deltaHb 20-30% / nadirHb 50-60 deltaHb 30-40% | 0.615619 | 0.096147 | 0.360522 | 1.051218 | -3.10622 | 0.1280 |
| nadirHb 60-70 deltaHb 20-30% / nadirHb 60-70 deltaHb 30-40% | 0.799875 | 0.10174 | 0.517329 | 1.236736 | -1.75557 | 0.9316 |
| nadirHb 60-70 deltaHb 20-30% / nadirHb 70-80 deltaHb 30-40% | 1.039278 | 0.226404 | 0.492714 | 2.192144 | 0.17685 | 1.0000 |
| nadirHb 60-70 deltaHb 20-30% / nadirHb 80-90 deltaHb 30-40% | 1.350335 | 0.464304 | 0.415751 | 4.385815 | 0.873516 | 1.0000 |
| nadirHb 70-80 deltaHb 20-30% / nadirHb 80-90 deltaHb 20-30% | 1.314801 | 0.17112 | 0.841803 | 2.053571 | 2.102866 | 0.7637 |
| nadirHb 70-80 deltaHb 20-30% / nadirHb 50-60 deltaHb 30-40% | 0.468222 | 0.120143 | 0.194385 | 1.127824 | -2.95725 | 0.1874 |
| nadirHb 70-80 deltaHb 20-30% / nadirHb 60-70 deltaHb 30-40% | 0.608362 | 0.100988 | 0.344483 | 1.074375 | -2.99388 | 0.1712 |
| nadirHb 70-80 deltaHb 20-30% / nadirHb 70-80 deltaHb 30-40% | 0.790445 | 0.134059 | 0.442102 | 1.413256 | -1.38655 | 0.9921 |
| nadirHb 70-80 deltaHb 20-30% / nadirHb 80-90 deltaHb 30-40% | 1.027026 | 0.270684 | 0.41632 | 2.533589 | 0.101182 | 1.0000 |
| nadirHb 80-90 deltaHb 20-30% / nadirHb 50-60 deltaHb 30-40% | 0.356117 | 0.133801 | 0.0983 | 1.290125 | -2.74804 | 0.2998 |
| nadirHb 80-90 deltaHb 20-30% / nadirHb 60-70 deltaHb 30-40% | 0.462703 | 0.124854 | 0.183574 | 1.166253 | -2.85608 | 0.2375 |
| nadirHb 80-90 deltaHb 20-30% / nadirHb 70-80 deltaHb 30-40% | 0.60119 | 0.126032 | 0.293149 | 1.232918 | -2.42725 | 0.5267 |
| nadirHb 80-90 deltaHb 20-30% / nadirHb 80-90 deltaHb 30-40% | 0.781127 | 0.182485 | 0.350848 | 1.739097 | -1.05736 | 0.9996 |
| nadirHb 50-60 deltaHb 30-40% / nadirHb 60-70 deltaHb 30-40% | 1.299301 | 0.182579 | 0.802837 | 2.102772 | 1.863257 | 0.8925 |
| nadirHb 50-60 deltaHb 30-40% / nadirHb 70-80 deltaHb 30-40% | 1.688183 | 0.47445 | 0.644547 | 4.421651 | 1.863257 | 0.8925 |
| nadirHb 50-60 deltaHb 30-40% / nadirHb 80-90 deltaHb 30-40% | 2.193458 | 0.92468 | 0.517466 | 9.297726 | 1.863257 | 0.8925 |
| nadirHb 60-70 deltaHb 30-40% / nadirHb 70-80 deltaHb 30-40% | 1.299301 | 0.182579 | 0.802837 | 2.102772 | 1.863257 | 0.8925 |
| nadirHb 60-70 deltaHb 30-40% / nadirHb 80-90 deltaHb 30-40% | 1.688183 | 0.47445 | 0.644547 | 4.421651 | 1.863257 | 0.8925 |
| nadirHb 70-80 deltaHb 30-40% / nadirHb 80-90 deltaHb 30-40% | 1.299301 | 0.182579 | 0.802837 | 2.102772 | 1.863257 | 0.8925 |
